# Diagnostic Accuracy of Deep Learning Models in Predicting Glioma Molecular Markers: A Systematic Review and Meta-Analysis

**DOI:** 10.3390/diagnostics15070797

**Published:** 2025-03-21

**Authors:** Somayeh Farahani, Marjaneh Hejazi, Sahar Moradizeyveh, Antonio Di Ieva, Emad Fatemizadeh, Sidong Liu

**Affiliations:** 1 Department of Medical Physics and Biomedical Engineering, School of Medicine, Tehran University of Medical Sciences, Tehran 14618-84513, Iran; mhejazi@sina.tums.ac.ir; 2 Centre for Health Informatics, Australian Institute of Health Innovation, Macquarie University, Sydney, NSW 2109, Australia; 3 Computational NeuroSurgery (CNS) Lab, Faculty of Medicine, Health and Human Sciences, Macquarie Medical School, Macquarie University, Sydney, NSW 2109, Australia; sahar.moradizeyveh@hdr.mq.edu.au (S.M.); antonio.diieva@mq.edu.au (A.D.I.); 4 Department of Electrical Engineering, Sharif University of Technology, Tehran 14588-89694, Iran; fatemizadeh@sharif.edu

**Keywords:** glioma, deep learning, MRI, molecular markers, radiomics

## Abstract

**Background/Objectives:** Integrating deep learning (DL) into radiomics offers a noninvasive approach to predicting molecular markers in gliomas, a crucial step toward personalized medicine. This study aimed to assess the diagnostic accuracy of DL models in predicting various glioma molecular markers using MRI. **Methods:** Following PRISMA guidelines, we systematically searched PubMed, Scopus, Ovid, and Web of Science until 27 February 2024 for studies employing DL algorithms to predict gliomas’ molecular markers from MRI sequences. The publications were assessed for the risk of bias, applicability concerns, and quality using the QUADAS-2 tool and the radiomics quality score (RQS). A bivariate random-effects model estimated pooled sensitivity and specificity, accounting for inter-study heterogeneity. **Results:** Of 728 articles, 43 were qualified for qualitative analysis, and 30 were included in the meta-analysis. In the validation cohorts, MGMT methylation had a pooled sensitivity of 0.74 (95% CI: 0.66–0.80) and a pooled specificity of 0.75 (95% CI: 0.65–0.82), both with significant heterogeneity (*p* = 0.00, I^2^ = 80.90–84.50%). ATRX and TERT mutations had a pooled sensitivity of 0.79 (95% CI: 0.67–0.87) and 0.81 (95% CI: 0.72–0.87) and a pooled specificity of 0.85 (95% CI: 0.78–0.91) and 0.70 (95% CI: 0.61–0.77), respectively. Meta-regression analyses revealed that significant heterogeneity was influenced by data sources, MRI sequences, feature extraction methods, and validation techniques. **Conclusions:** While the DL models show promising prediction accuracy for glioma molecular markers, variability in the study settings complicates clinical translation. To bridge this gap, future efforts should focus on harmonizing multi-center MRI datasets, incorporating external validation, and promoting open-source studies and data sharing.

## 1. Introduction

Gliomas are the most common primary brain tumors, characterized by significant histological and molecular variability. The 2021 WHO Classification of Tumors of the Central Nervous System emphasizes the need for accurate identification of molecular markers to ensure precise glioma classification [1]. Currently, genetic profiling of tumor tissue, obtained through a biopsy or surgical resection, is the standard approach. However, these invasive procedures carry risks such as bleeding and infection and may not fully capture the heterogeneity of the tumor [2]. As a result, noninvasive methods for obtaining genetic and histologic information are critically important. Magnetic resonance imaging (MRI) offers a noninvasive alternative and is particularly important due to its widespread clinical use and its ability to capture diverse tumor characteristics across various imaging sequences [3]. Despite its advantages, MRI interpretation remains challenging due to human limitations and the presence of radiological mimics, such as inflammation, stroke, and infections [4].

Radiomics aims to bridge the gap by linking high-throughput quantitative image features with molecular phenotypes [5]. The radiomics pipeline involves the following critical steps: image processing and segmentation, feature extraction and selection, and model classification, each significantly influencing outcomes [6,7]. Concerns over the reliability of manually segmented, handcrafted features have driven the integration of deep learning (DL) into the radiomics framework. DL methods can either automate various tasks within this framework, reducing human bias and error, or fully replace the conventional radiomics pipeline by enabling direct classification without relying on predefined features [6]. In this article, we refer to both approaches as DL-based radiomics. Since the integration of DL into radiomics, numerous studies have explored the molecular markers associated with various gliomas [8,9,10,11,12]. Given the extensive research in this field, a systematic review and meta-analysis are needed to critically evaluate and quantify the existing data. Such an analysis would help determine whether these advanced approaches can be effectively translated into clinical practice.

In our previous systematic review [8], we thoroughly examined two key genetic markers—isocitrate dehydrogenase (IDH) mutations and 1p/19q codeletion—which are fundamental to glioma diagnosis and classification [9]. Building upon this work, the present study shifts focus to other molecular abnormalities that are often neglected and have been the subject of fewer investigations. Existing systematic reviews predominantly focus on O6-methylguanineDNA methyltransferase (MGMT) gene promoter methylation [10,11] and present contradictory outcomes [12,13]. Additionally, reviews assessing other genetic and epigenetic abnormalities [14,15,16] are either based on conventional radiomics or need to be updated. This study addresses these gaps by comprehensively reviewing MRI-based DL models for predicting a broad spectrum of glioma molecular markers. These markers include mutations in phosphatase and tensin homolog (PTEN), alpha-thalassemia/mental retardation syndrome X-linked (ATRX), and telomerase reverse transcriptase (TERT), as well as CDKN2A/B homozygous deletions and epidermal growth factor receptor (EGFR) amplification. Additionally, we reviewed the high expression of Ki-67, p53 proteins, the Synaptophysin (SYP) gene combined aneuploidy of chromosomes 7 and 10 and MGMT methylation. More importantly, we performed an extensive meta-regression analysis to account for inter-study heterogeneity while quantitatively examining the relationship between study settings and diagnostic accuracy.

## 2. Materials and Methods

This study involves a systematic literature review and meta-analysis following PRISMA guidelines, and no ethical approval was required [17]. This study is registered on PROSPERO (CRD42024542505).

### 2.1. Search Strategy and Study Selection

We conducted a comprehensive search of PubMed, Scopus, Ovid, and Web of Science up to 27 February 2024 and reviewed article bibliographies (Supplementary Section S1). Studies were included if they involved any glioma grade, predicted at least one of the aforementioned molecular markers using MRI, and applied DL algorithms within a radiomics framework. For studies assessing multiple biomarkers, each biomarker was analyzed separately. To be included in the meta-analysis, studies needed to report sufficient data to construct a 2 × 2 diagnostic table (true positives, false positives, false negatives, and true negatives). Studies lacking adequate validation metrics were included in the qualitative synthesis only. We excluded non-original research and non-human studies. Two reviewers (S.F. and S.M.) independently screened the abstracts and full texts, resolving disagreements through discussion. Initial duplicate removal and screening were conducted using Zotero (version 6.0.36).

### 2.2. Data Extraction

Data on the study design, patient characteristics, utilized datasets, MRI sequences, data augmentation techniques, and computational methodologies were independently collected by two reviewers (S.F. and S.M.) using a standardized form (Supplementary Section S2). Performance metrics, including the diagnostic confusion matrix, were obtained from training (internal validation) and unseen validation datasets. Missing data prompted contacting the corresponding authors; since there was no response, the metrics were computed using other provided metrics and patient counts for altered and intact biomarkers. For studies presenting only receiver operating characteristic curves, the top-left method was used to determine sensitivity and specificity. Values were rounded to the nearest whole number, potentially causing slight deviations from the true sensitivity and specificity. When multiple DL models were evaluated in one study, the best-performing one was chosen.

### 2.3. Quality Assessment

The risk of bias and applicability concerns were assessed using a modified QUADAS-2 tool [18], adapted for DL-based radiomics studies by incorporating elements from the Checklist for Artificial Intelligence in Medical Imaging (CLAIM) and the radiomics quality score (RQS) (Supplementary Section S3). QUADAS-2 evaluates four domains: patient selection, index test, reference standard, and flow/timing. The key considerations included standardized imaging protocols, appropriate data selection, handling of missing data, reliable reference standards, and minimizing genotype imbalances. The index test assessment emphasized segmentation consistency and model robustness. To ensure clinical generalizability, we addressed applicability concerns by validating models on unseen datasets. For each article, methodology, quality, translatability, strengths, and limitations were further evaluated using the RQS, a 16-component score ranging from −8 to 36 [19] (Supplementary Section S4). Two reviewers (S.F. and S.M.) independently conducted the QUADAS-2 and RQS assessments. When data were insufficient, the authors were contacted for clarification. The RQS scores were averaged for discrepancies of ≤2 points, while larger discrepancies (>2 points) were resolved through a discussion.

### 2.4. Statistical Analysis

The meta-analysis assessed a DL model’s ability to detect molecular markers, with significance at *p* < 0.05. We calculated pooled sensitivity and specificity and derived the summary receiver operating characteristic (SROC) curve using a bivariate random-effects model when at least five studies were available. Heterogeneity was assessed through Cochran’s Q test, I^2^ statistic, prediction intervals, and the Spearman correlation coefficient (SCC) between sensitivity and the false positive rate (FPR), indicating a threshold effect when the SCC > 0.6 [20,21]. Subgroup analysis was conducted only for MGMT methylation due to the insufficient number of included studies for other biomarkers. It investigated sources of heterogeneity based on the glioma grade, data source (single or multi-center), the inclusion of clinical information, data augmentation, use of pretrained models, image segmentation, feature extraction methods, level of DL integration in the radiomics pipeline, MRI sequences, and validation methods when sufficient studies were available [22].

We conducted a leave-one-out meta-analysis to assess each study’s impact on the overall effect size. Publication bias was evaluated using funnel plots and Egger’s test. If potential bias was detected, the Trim and Fill method by Duval and Tweedie was applied to adjust the pooled sensitivity and specificity estimates. We calculated the statistical power across various effect sizes to ensure the robustness of our findings [23]. Analyses were conducted using the R packages “mada”, “dmetar”, “metameta”, and “metafor” in R Stats v4.4.1, along with the MetaBayesDTA web application (v1.5.2) [24].

## 3. Results

### 3.1. Study Characteristics

**Figure 1:**
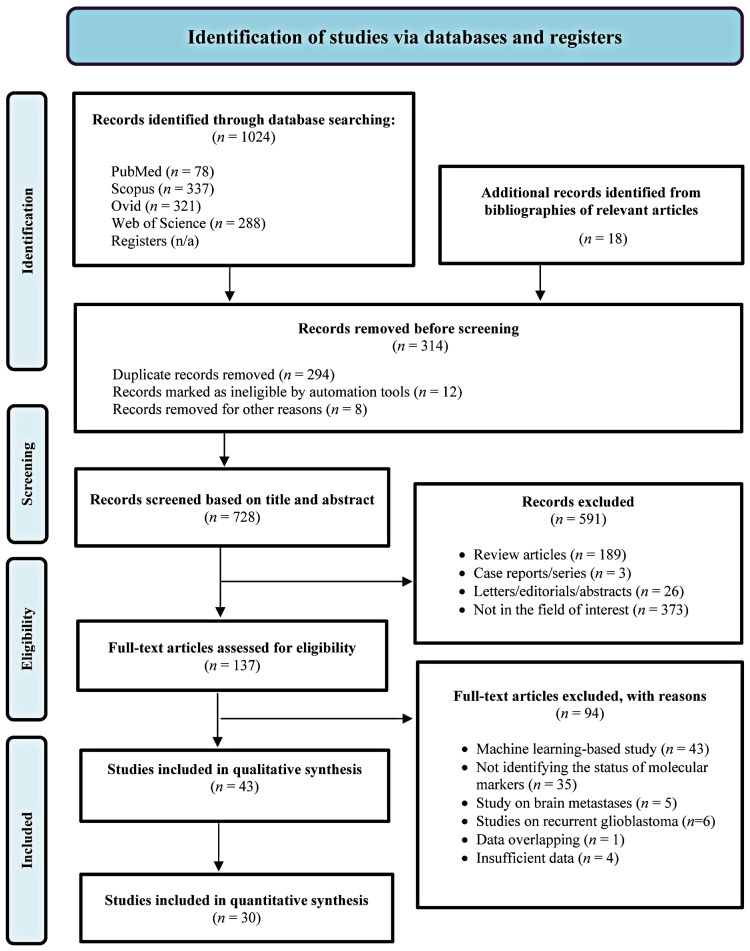
Flow diagram of the study selection process.

Seven hundred and twenty-two unique articles were initially identified through primary searches and relevant study bibliographies. Following screening and full-text reviews, 43 studies were eligible for qualitative analysis, of which 30 were included in the meta-analysis (Figure 1). One non-English study was included in our review [25].

**Figure 2:**
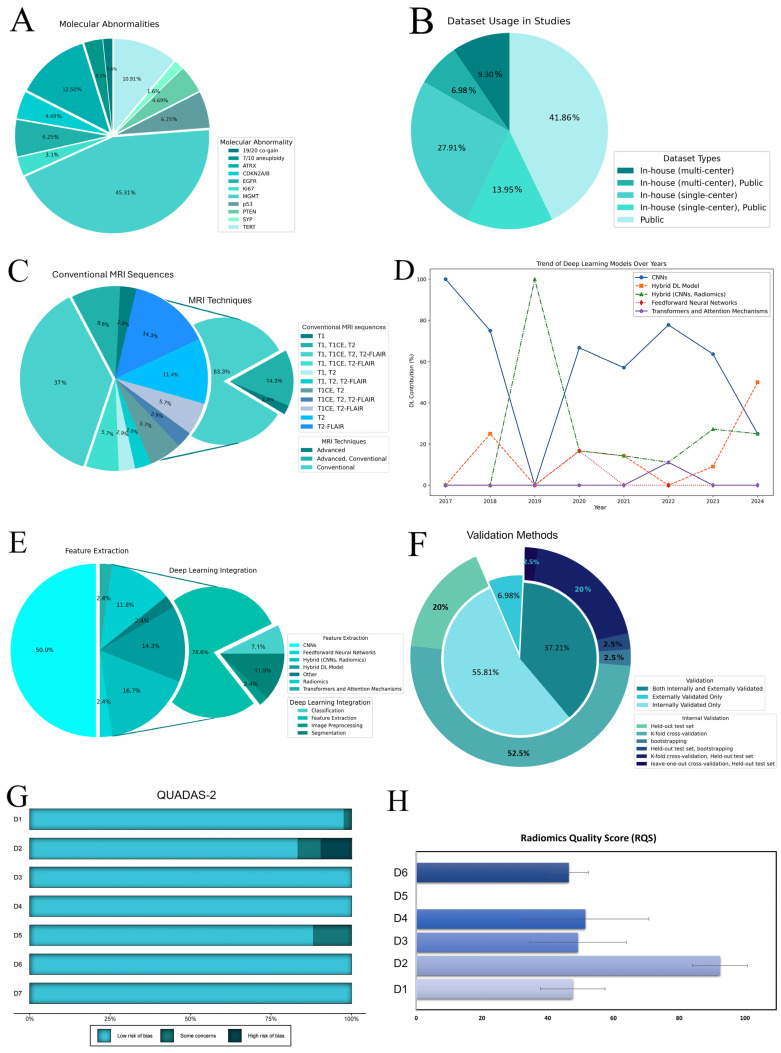
(**A**) Distribution of molecular abnormalities among studies. (**B**) Dataset usage in studies. (**C**) MRI sequence usage in studies. (**D**) The trend of deep learning models over the years. (**E**) DL integration in included studies. The charts display the use of different DL models for feature extraction, with CNNs being the most common. Integration mainly involves classification and feature extraction, with less focus on image preprocessing and segmentation. (**F**) The validation methods used in the studies depict types of validation and distribution of internal validation techniques. The main pie chart shows the overall validation approaches, while the outer ring details specific internal methods. (**G**) The QUADAS-2 results. The chart shows the risk of bias across different domains (D1–D7). The domains are categorized as follows: D1–D4 for risk of bias, with D1 (patient selection), D2 (index test), D3 (reference standard), and D4 (flow and timing); and D5–D7 for applicability concerns, with D5 (patient selection), D6 (index test), and D7 (reference standard). (**H**) RQS across six domains (D1 (protocol quality), D2 (feature selection and validation), D3 (biologic/clinical validation and utility), D4 (model performance index), D5 (level of evidence), and D6 (open science and data)) for included articles are depicted as average percentage scores with standard errors. Abbreviations: DL, deep learning; CNNs, convolutional neural networks; D1–D7, Domain 1 to Domain 7; QUADAS-2, quality assessment of diagnostic accuracy studies-2; RQS, radiomics quality scores; MGMT, O6-methylguanine-DNA methyltransferase; ATRX, alpha thalassemia/mental retardation syndrome X-linked; TERT, telomerase reverse transcriptase; 19/20 co-gain, 19/20 chromosomes co-gain; CDKN2A/B, CDKN2A/B homozygous deletion; SYP, Synaptophysin gene expression; EGFR, epidermal growth factor receptor; PTEN, phosphatase, and tensin homolog.

Table 1 summarizes the main study characteristics. China and the USA dominate the publication volume, conducting about 63% of experiments in this field. Additionally, the studies varied widely in sample size, ranging from 42 to 985 patients. Seven studies focused on multiple molecular markers [26,27,28,29,30,31,32], with 45.31% targeting MGMT methylation, followed by ATRX (12.50%) and TERT mutations (10.94%). Other markers were studied less frequently, accounting for about 31% in total (Figure 2A). Over half of the studies focused on high-grade gliomas (HGG), with 80% emphasizing MGMT prediction. Furthermore, 9.30% studied low-grade gliomas (LGG) in grades 2 and 3. Approximately one-third of the studies explored a broader spectrum, including both LGG and HGG, primarily focusing on MGMT classification.

Private (single and multi-center) and public datasets (mainly The Cancer Imaging Archive (TCIA)) [67,68] were employed, with over 20% utilizing a hybrid of both (Figure 2B). Conventional data augmentation was applied in 48.84% of the studies to address overfitting and the genotype class imbalance. Pretrained models, primarily ImageNet, were used in 53.49% of the studies, with 20 models incorporating non-radiomics data such as age and sex. The widespread use of public datasets influenced the MRI sequence selection (Figure 2C). Conventional sequences were predominantly used, with only one study exclusively utilizing the advanced technique, specifically dynamic susceptibility contrast scans [39]. The contrast-enhanced T1 modality was the most frequently employed, appearing in 71.43% of the studies alongside other MR scans (Supplementary Section S11).

Our review highlights a rise in the use of DL for radiomics since 2017 [33]. In the early stages, convolutional neural networks (CNNs) such as AlexNet, DenseNet, and EfficientNet dominated the field—accounting for 100% of the methods in 2017 and 75% in 2018. By 2020, although CNNs remained the most common approach at 66%, hybrid models began to emerge as alternatives [42].

Many studies utilize CNNs to directly classify molecular markers from MRI [35,36,51,52,58]. These models typically consist of multiple convolutional layers for feature extraction, followed by fully connected layers for prediction. For example, Korfiatis et al. [33] compared three residual network architectures (ResNet50, ResNet34, and ResNet18) for predicting the MGMT methylation status without requiring explicit tumor segmentation. In another approach, Han and Kamdar [36] employed a bi-directional convolutional recurrent neural network (CRNN) that first extracts slice-level features through convolutional, pooling, and fully connected layers and then uses a bi-directional GRU to capture inter-slice spatial and sequential information in 3D MRI scans.

In parallel, a number of studies have integrated DL-based tumor segmentation into their pipelines, either as preprocessing within the conventional radiomics framework or as part of a fully DL-based approach. Segmentation-focused architectures, such as U-Net variants, are commonly used to delineate tumor regions so that a subsequent classification can focus on tumor-specific pixels. Some studies extract radiomics features from these segmented subregions [27,29,34,38,45], while others derive deep features directly from the segmented areas [30,38,45]. Several works have explored hybrid models that combine radiomics and deep features [30,38]. For instance, Calabrese et al. [30] used two parallel processing limbs—a CNN limb employing a 3D multiscale convolutional autoencoder with residual bottleneck blocks and max-pooling layers, and a radiomics limb based on a random forest classifier trained on a curated set of handcrafted features. Averaging the outputs of these limbs resulted in improved prediction performance over the models relying solely on one feature type.

In 2022, transformers and attention mechanisms introduced a new paradigm in the field (Figure 2D). Xu et al. [31] developed a multitask Vision Transformer (ViT) framework to predict multiple molecular expressions (IDH, MGMT, Ki67, and P53) from MR images. Their approach reshapes the input image into a sequence of flattened 2D patches, which are projected into a latent embedding space. Positional and classification embeddings are then added to retain spatial and task-specific information before processing the sequence through multiple transformer blocks—each composed of multi-head self-attention and multilayer perceptron layers. Finally, task-specific fully connected layers output predictions for each molecular marker. Compared to CNN-based models, the ViT architecture employs a global self-attention mechanism to capture long-range dependencies and salient features across the entire image, potentially enhancing classification performance [31,58].

Overall, most DL methods have been applied primarily for feature extraction in radiomic workflows, with fewer studies focusing on image processing [52], tumor segmentation [27,28,29,34,54], and classification [39,48,49] (Figure 2E). Notably, CNNs have also been the primary method for tumor segmentation, used in 32.56% of the studies, while manual and semi-automatic methods were employed in 27.91% and 13.95% of the studies, respectively. Regarding software, Python was utilized in 41 out of 43 studies, with Matlab used in only 2. Among the DL frameworks, PyTorch was most popular at 30.00%, followed by Keras at 21.00%, and TensorFlow at 12.00%; however, 10 studies did not specify the framework or share code (Table 1).

In model development and evaluation, 46.51% of the studies conducted external validation, while 23 experiments did not. Additionally, 54.76% relied solely on internal validation, and 16 studies utilized both internal and external validation approaches (Figure 2F). Exclusive external validation was rare, observed in only three studies [34,50,53].

### 3.2. Quality Assessment

According to the QUADAS-2, four studies had a high risk of bias, primarily due to limited segmentation methods [26,38,39] or the absence of resampling techniques [34] (Figure 2G; Supplementary Section S3). The median RQS score was 16, ranging from 11 (25.00%) to 22 (50.00%) out of 36. In Domain 1 (average score: 2.4 ± 0.49), studies described imaging protocols, but none included multiple time points or phantom studies; however, 17 studies performed multiple segmentations. Domain 2 achieved the highest score, with 72% of the studies validating findings on unseen datasets. Domain 3 (average score: 2.93 ± 0.88) saw approximately 40% of the studies utilizing multivariable analysis with non-radiomics features and 48.84% discussing biological correlates, but only one study employing decision curve analysis to assess clinical utility [64]. In Domain 4, around half of the studies conducted cut-off analyses, with five studies reporting calibration statistics [26,32,59,62,64]. All studies were retrospective, lacking prospective validation or cost-effectiveness analysis. While 65.12% of the experiments utilized open-source data, only 13 studies made their code accessible (Figure 2H; Supplementary Section S4).

### 3.3. Publication Bias and Statistical Power

The funnel plot asymmetry and Egger’s test suggested potential publication bias for the MGMT studies in validation cohorts (*p* = 0.00) but not in training sets or for the ATRX and TERT studies (*p* > 0.05). However, except for MGMT in the validation datasets, the number of studies in other groups was too small (<10) to test for small study effects. Given the potential presence of publication bias for MGMT prediction, we performed the Trim and Fill method by Duval and Tweedie to further explore and adjust for bias in the pooled sensitivity and specificity estimates (Supplementary Section S6).

The power analysis of the included studies revealed variability in detecting changes in sensitivity and specificity, with most studies demonstrating high power for clinically meaningful effect sizes (Supplementary Section S9).

### 3.4. Sensitivity Analysis

The sensitivity analysis identified significant variability in the pooled estimates for MGMT methylation in the validation cohorts. The sensitivity was stabilized at 0.71 [95% CI: 0.66–0.76] with a prediction interval of 0.54–0.84 (I^2^ = 54.3%, *p* = 0.00) when five studies [27,29,43,48,59] showing influential effects were removed. When eight outliers [13,27,29,43,48,49,51,59] were excluded, the specificity was increased to a point estimate of 0.75 [95% CI: 0.68–0.81], with a prediction interval of 0.49–0.90 (I^2^ = 63.7%, *p* = 0.00). No outliers were detected for other molecular abnormalities (Supplementary Section S5).

### 3.5. Prediction of Molecular Marker Status

**Figure 3:**
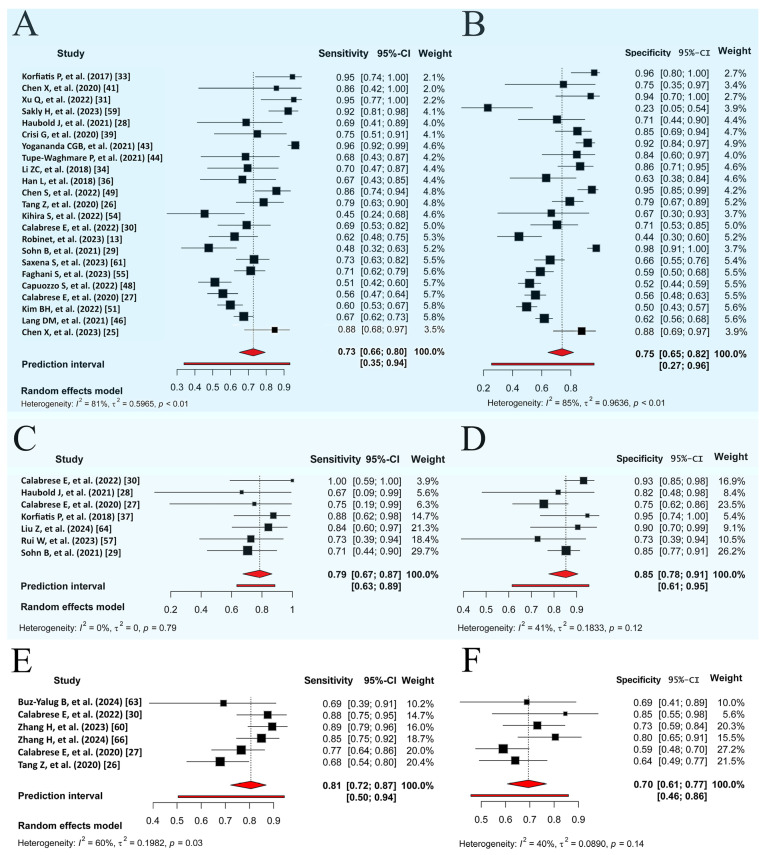
Random forest visualization of validation cohorts for molecular marker prediction. (**A**) Sensitivity for MGMT methylation prediction. (**B**) Specificity for MGMT methylation prediction. (**C**) Sensitivity for ATRX mutation prediction. (**D**) Specificity for ATRX mutation prediction. (**E**) Sensitivity for TERT mutation prediction. (**F**) Specificity for TERT mutation prediction. Each plot shows sensitivity and specificity with a 95% CI and study weights. Pooled estimates and prediction intervals under a random effects model are at the bottom. Numbers represent pooled estimates with a 95% CI, depicted by the horizontal lines. Abbreviations: MGMT, O6-methylguanine-DNA methyltransferase; ATRX, alpha thalassemia/mental retardation syndrome X-linked; TERT, telomerase reverse transcriptase; CI, confidence interval.

**Figure 4:**
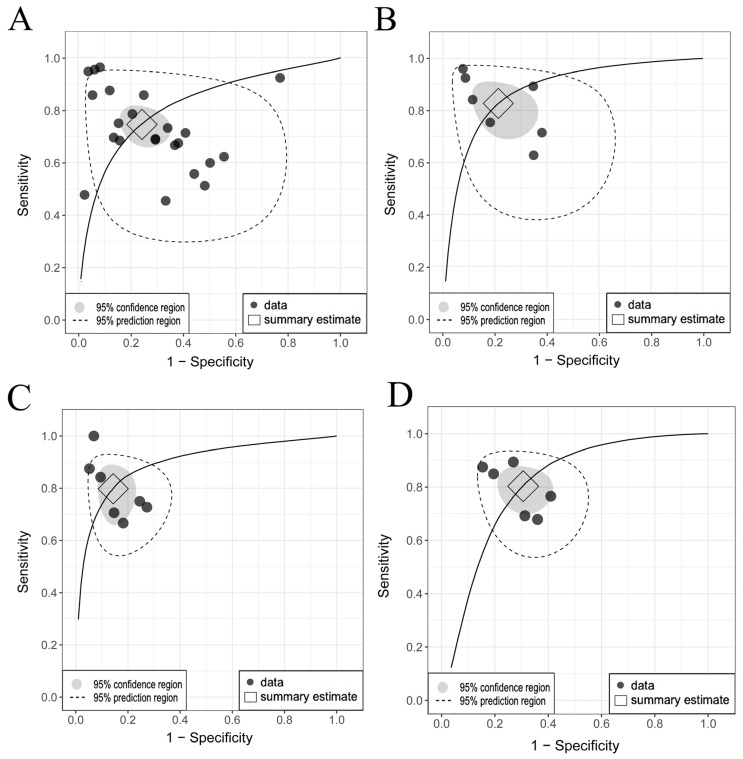
Comparison of SROC curves for genetic abnormalities in training and validation cohorts. (**A**) MGMT validation: pooled sensitivity 0.74 [95% CI, 0.66–0.80], specificity 0.75 [95% CI, 0.65–0.82]. (**B**) MGMT training: pooled sensitivity 0.83 [95% CI, 0.72–0.90], specificity 0.79 [95% CI, 0.68–0.88]. (**C**) ATRX validation: pooled sensitivity 0.79 [95% CI, 0.67–0.87], specificity 0.85 [95% CI, 0.78–0.91]. (**D**) TERT validation: pooled sensitivity 0.81 [95% CI, 0.72–0.87], specificity 0.70 [95% CI, 0.61–0.77]. Considerable differences between the 95% confidence and prediction regions, particularly for MGMT methylation, highlight significant between-study heterogeneity. Abbreviations: SROC, summary receiver operating characteristic; MGMT, O6-methylguanine-DNA methyltransferase; ATRX, alpha thalassemia/mental retardation syndrome X-linked; TERT, telomerase reverse transcriptase; CI, confidence interval.

MGMT methylation prediction had consistent sensitivity and specificity across both the training and validation cohorts, with SCC values of −0.67 (95% CI: −0.95 to 0.16) and −0.31 (95% CI: −0.64 to 0.11), respectively (Figure 3A,B, Figures S7 and S8). The validation cohort showed significant heterogeneity (*p* = 0.00, I^2^ = 80.90–84.50%), with a pooled sensitivity and specificity of 0.74 and 0.75, respectively. The prediction intervals showed a sensitivity range of 0.35 to 0.94 and a specificity range of 0.27 to 0.96, implying that true effect sizes would be within these intervals in 95% of similar populations. To address potential publication bias in the validation cohort of MGMT prediction, the Trim and Fill method was applied, resulting in an adjusted pooled sensitivity and specificity of 0.89 (95% CI: 0.78–1.00, I^2^ = 94.3%, *p* = 0.0001) and 0.91 (95% CI: 0.79–1.00, I^2^ = 95.9%, *p* = 0.03), respectively. This adjustment accounted for 11 imputed studies to achieve funnel plot symmetry (Supplementary Section S6). Training diagnostic performance for MGMT prediction also demonstrated significant heterogeneity in sensitivity and specificity (Table 2), as displayed in the SROC curves (Figure 4A,B), highlighting significant differences between the confidence and prediction regions.

The prediction performance of the ATRX and TERT statuses in the validation sets (Figure 3C–F) showed no significant threshold between sensitivity and specificity, with SCCs of −0.78 (95% CI: −0.96 to −0.06) and −0.70 (95% CI: −0.96 to 0.25), respectively. The pooled sensitivities were 0.79 and 0.81, and the specificities were 0.85 and 0.70 for ATRX and TERT prediction, respectively. Significant between-study heterogeneity was observed only across the TERT studies for sensitivity (PI = 0.51–0.94, I^2^ = 60.20%, *p* = 0.03), as shown in the SROC curves (Figure 4C,D) and detailed in Table 2.

Due to the limited number of included studies, a meta-analysis was infeasible for other molecular markers; however, their sensitivity and specificity ranges are reported in Table 2. EGFR mutation and p53 protein expression were assessed in three studies each, followed by PTEN loss, CDKN2A/B deletion, and chromosome 7/10 aneuploidy in two studies each. In contrast, Ki67 and SYP expression were mentioned in only one study each. The sensitivity ranged from 0.57 to 0.98, and the specificity ranged from 0.59 to 0.95 for these markers.

### 3.6. Meta-Regression and Subgroup Analysis

Given the heterogeneity of the MGMT studies and sufficient validation cohort data, we investigated the impact of various covariates (Table 3). Although studies that included both HGG and LGG showed higher sensitivity than those that focused solely on HGG, the difference was statistically insignificant. Single-center datasets had higher sensitivity and specificity than those that combined public datasets. While the CNN-derived features had higher sensitivity than the conventional radiomics, no significant difference existed between the DL-based and manual segmentation methods. DL for feature extraction achieved significantly higher sensitivity than DL solely for segmentation. Pretrained DL models reduced specificity without affecting sensitivity, whereas incorporating non-radionics data and implementing data augmentation had no significant effect. Furthermore, the k-fold cross-validation models had higher sensitivity than those using a held-out test approach (Supplementary Section S10).

## 4. Discussion

Our systematic review provides a comprehensive and rigorous assessment of the current state of MRI-based DL in predicting genetic abnormalities in gliomas. Consistent with previous studies [10,16], the findings indicate that DL-based radiomics demonstrates moderate to high diagnostic accuracy for key molecular markers, highlighting its potential as a noninvasive tool for glioma characterization. However, the substantial variability in outputs and study designs underscores the necessity for standardized approaches to enhance reproducibility and clinical applicability.

Conducting a statistical power analysis for meta-analysis studies is crucial for assessing data reliability and generalizability [23]. Our statistical power analysis revealed that the majority of studies possessed sufficient power to detect clinically meaningful effect sizes, particularly in the prediction of MGMT methylation [40,46,48] and TERT mutation [60,66]. High-powered studies typically employed larger sample sizes and robust validation cohorts, thereby enhancing the reliability of their findings. In contrast, studies with smaller sample sizes or limited validation datasets exhibited reduced statistical power [28,54]. Overall, the studies were sufficiently powered to detect the estimated effect sizes across the molecular markers analyzed.

The availability of public datasets has fueled growth in glioma DL-based radiomics, with nearly half of all studies using public datasets, most notably TCIA. These large-scale datasets enable the training and validation of complex DL models, fostering innovation and facilitating comparative analyses across studies. However, the integration of public datasets with institutional data remains limited due to the discrepancies in imaging protocols and patient demographics. Our subgroup analysis showed that models trained and validated on single-center datasets outperformed multi-center datasets. This highlights the critical need for data harmonization efforts to maximize the benefits of open data, ensuring consistency and reliability in radiomics analyses. Several harmonization techniques have been proposed [69]. Feature-level methods like ComBat standardize radiomic feature distributions by correcting for scanner-induced “batch effects”, thereby improving consistency and classification accuracy [70]. On the image side, DL approaches such as cycle-consistent GANs and style transfer methods can transform images into a common domain, though careful validation is necessary to avoid artifacts [69,71]. In addition, physics-based corrections—such as bias field inhomogeneity correction, noise filtering, and intensity normalization techniques (e.g., z-score scaling or histogram matching)—serve as fundamental steps to reduce variability at the source [72].

The shift from classical radiomics to DL-based approaches has transformed analysis workflows. Our meta-regression shows DL models outperform traditional radiomics. Understanding this variability requires comparing traditional radiomics and DL models, considering their respective strengths and limitations. In traditional radiomics, machine learning (ML) models—such as support vector machines, random forests, and k-nearest neighbors—serve solely as classifiers, correlating predefined radiomic features extracted from the region of interest (ROI) with clinical endpoints [7]. ML methods generally require less training data and offer greater interpretability [73], making it easier to understand how input variables influence predictions. However, they are limited in capturing complex patterns [73,74,75]. Additionally, conventional radiomics faces challenges with the stability of radiomic feature computation, which is compromised by the lack of standardization and high variability in image acquisition protocols, ROI definition, and image processing techniques. These issues are particularly pronounced in MRI, where image intensities are influenced by factors such as scanner manufacturer, magnetic field strength, sequence protocol, and image reconstruction [6]. Although initiatives like the Image Biomarker Standardization Initiative (IBSI) [76] have sought to mitigate variability, challenges related to the variability of image quality persist.

In contrast, DL methods can operate in an end-to-end manner or be integrated into specific stages of the radiomics pipeline, including data augmentation, image preprocessing, ROI segmentation, feature extraction, and classification [6]. In terms of deep features, these models are capable of extracting intricate, non-linear representations that traditional ML struggles to capture. However, this advantage comes with challenges, including the “black-box” nature of DL models, where internal decision-making is not transparent [74,75,77,78]. This limited interpretability can compromise clinician trust and accountability. Additionally, the implicit DL-based feature representations highlight the need for new guidelines to ensure robustness and clinical utility [79]. To address this issue, integrating explainable artificial intelligence techniques—such as saliency mapping [80] and Shapley Additive exPlanations methods [81]—can provide much-needed transparency and facilitate a better understanding of the decision-making processes.

Another challenge of DL models is their higher computational costs. Conventional radiomics models are computationally lightweight—training a ML classifier on a few hundred features from a few hundred patients can be completed in seconds to minutes on a standard CPU without the need for specialized hardware. DL techniques, however, require extensive processing power and memory in training and inference (prediction), especially when dealing with large 3D medical images. For instance, training a 3D CNN, such as a 3D U-Net on MRI volumes, can take from several hours to days depending on the dataset size and hardware capabilities, often necessitating GPUs with at least 16–24 GB of VRAM to handle the large memory requirements during training [82]. Moreover, in clinical practice, the inference speed is critical for the workflow. While inference times on GPUs are typically within seconds to a minute, processing on CPUs can significantly slow down the prediction process, making them unsuitable for real-time applications. To mitigate these issues, strategies such as mixed-precision training [83], quantization [84], model pruning [85], development of more efficient architectures (e.g., using MobileNet [66] or EfficientNet [61]), and pipeline optimization [86] have been implemented. Using these methods, researchers have managed to deploy DL algorithms within the constraints of hospital infrastructure, achieving near-real-time inference while maintaining acceptable levels of accuracy [86].

Our quality assessment highlights several areas for improvement. The median RQS score of 16 reflects moderate methodological quality across studies. Although most studies detailed imaging protocols, none incorporated multiple time points or phantom studies to assess inter-scanner variability—a critical factor for ensuring reproducibility. Additionally, less than half of the studies performed multiple segmentations, with the remainder relying on a single expert despite the well-documented impact of inter- and intra-observer variability on extracted features [6,87]. While 72% of studies validated their models on unseen datasets, the absence of independent test sets in half raises concerns about generalizability. In the model performance domain, only five studies reported calibration statistics that are vital for assessing the agreement between predicted and observed outcomes. Notably, all studies were retrospective, lacking both prospective validation and cost-effectiveness evaluations that are crucial for demonstrating real-world clinical utility. By adapting the QUADAS-2 tool to include questions on multiple segmentation, resampling techniques, and validation with unseen datasets, we identified significant bias in some studies—highlighting challenges to the generalizability and clinical application of these models.

These methodological limitations, consistent with previous reviews [12,88,89], indicate that while DL-based radiomics demonstrates promising accuracy in predicting glioma molecular markers, several challenges must be addressed to fully realize its clinical potential. One major challenge is the need for extensive external validation to ensure the robustness and generalizability of the model in real-world conditions. Performance often declines when models are tested on different settings due to dataset shift [90]. Our meta-regression analysis confirmed this issue, showing significant variability across different validation methods. To address this, external test cohorts should always be included, and when performance drops, techniques like domain adaptation can help improve generalizability. Additionally, to ensure robustness and fairness across diverse patient populations, models should be stress-tested on different subgroups during development and validation. This is particularly important given that fewer than 4% of FDA-approved AI devices report race or ethnicity data [91]. Furthermore, prospective validation through real-time studies or clinical trials is crucial to show that DL models not only achieve high diagnostic accuracy but also improve patient outcomes compared to standard care. Unlike retrospective studies, prospective validation captures the full clinical workflow—data acquisition, model inference, and clinician decision-making—without hindsight bias, providing a more realistic assessment of the model [92].

Given the limitations in data availability and generalizability, researchers are turning to foundation DL models. These large-scale models are pretrained on vast, unlabeled datasets using self-supervised learning, helping to overcome the scarcity of expert annotations in medical applications while enhancing performance in downstream tasks [93]. In radiology, RadImageNet—a CNN pretrained on 1.35 million medical images across multiple modalities—provides robust, general features [94]. More specifically, BrainSegFounder, based on the SWIN-UNETR architecture and pretrained on over 42,000 brain MRIs, has demonstrated superior segmentation accuracy compared to baseline models [95]. Additionally, a recently developed ViT-based foundation model for brain tumor imaging biomarkers was trained on 57,000 MRI volumes and fine-tuned for tasks such as IDH mutation and 1p/19q codeletion classification [96]. Overall, these foundation models offer a promising starting point, requiring fewer glioma-specific cases for fine-tuning while potentially outperforming traditional DL models in accuracy, robustness, and generalizability.

Moreover, regulatory challenges remain a significant barrier to deploying DL-based radiomics in healthcare. For instance, traditional approval pathways like the FDA’s 510 (k) clearance are designed for static devices and struggle with continuously evolving AI algorithms that adapt post-deployment [97]. In Europe, the Medical Device Regulation (MDR) classifies many AI-based tools as high-risk, demanding conformity assessments that are hard to apply to adaptive models [97]. Additionally, data privacy laws add complexity, and the limited transparency regarding clinical benefit and patient safety in many ML or DL tools further complicates regulatory approval [98]. These challenges highlight the need for adaptive regulatory frameworks and robust post-market surveillance systems.

Finally, integrating DL into clinical workflows requires seamless compatibility with electronic health records, proper training for clinicians, and a strong IT infrastructure to support ongoing model updates and real-time data flow. To ensure clinical effectiveness and cost-efficiency, pilot programs should be followed by multi-center prospective studies [99,100]. Implementing DL into healthcare comes with various costs, including hardware, software, staffing, and maintenance. Hospitals need to carefully assess these costs against potential benefits. However, most studies focus on technical performance, with few conducting formal cost-benefit analyses. Our quality analysis found that only one study used decision curve analysis to measure clinical net benefit. Addressing these challenges will help transition DL models from research to clinical practice, ultimately enabling personalized treatments and improving patient outcomes in oncology.

This systematic review has several limitations. We focused on top-performing DL models and broadly categorized them due to the limited number of experiments. However, variations within a single study, such as DL methods or MRI sequences, were treated as separate experiments for detailed subgroup analysis. While our meta-regression addressed some heterogeneity, it could not explain all the discrepancies. These findings are observational rather than causal due to a lack of randomization between studies, a common problem in meta-analyses [20]. Other confounding variables may influence the findings, especially given the small number of studies on some subgroups. Lastly, our review excluded gray literature, although we thoroughly searched major databases with no date or language restrictions.

## 5. Conclusions

In conclusion, while DL-based radiomics shows significant promise in predicting glioma molecular markers, its clinical translation is challenged by data heterogeneity, reduced external performance, computational limitations, and regulatory barriers. Multi-center studies must begin by standardizing MRI acquisition and preprocessing, followed by applying transfer learning or domain adaptation alongside calibration analyses. Prospective validations are needed to provide a realistic assessment of the model. Additionally, integrating phantom and multi-time point tests will enhance model robustness. Regular inter-center communication, improved open-source code practices, and innovative approaches such as federated learning, shared platforms, and foundation DL models are essential for developing clinically translatable DL models while ensuring patient privacy and regulatory compliance.

## Figures and Tables

**Table 1:** Characteristics of the included studies. All studies had a retrospective design. Abbreviations: Total no. pts, total number of patients; MGMT, O6-methylguanine-DNA methyltransferase; ATRX, alpha thalassemia/mental retardation syndrome X-linked; TERT, telomerase reverse transcriptase; 19/20 co-gain, 19/20 chromosomes co-gain; CDKN2A/B, CDKN2A/B homozygous deletion; SYP, Synaptophysin gene expression; EGFR, epidermal growth factor receptor; PTEN, phosphatase, and tensin homolog; DL, deep learning; CNNs, convolutional neural networks; T1, T1-weighted imaging; T2, T2-weighted imaging; T1CE, T1-weighted contrast-enhanced imaging with gadolinium; T2-FLAIR, T2-weighted fluid-attenuated inversion recovery imaging; DSC, dynamic susceptibility contrast MR perfusion; SWI, susceptibility-weighted imaging; DWI, diffusion-weighted imaging; ASL, Arterial Spin Labeling; 2D 55-direction HARDI, 2D 55-direction high angular resolution diffusion imaging; RQS, radiomics quality score; AUC, area under the curve.

Study	Total no. pts	Genes	Grade	Dataset	MRI	Segmentation	Feature Extraction	Software (Framework)	Internal Validation	External Validation	RQS (%)
Korfiatis P, et al. (2017) [33]	155	MGMT	4	In-house (single-center)	T2	Not undertaken	CNNs	Python (Keras)	K-fold cross-validation, held-out test set	No	25.00
Li ZC, et al. (2018) [34]	193	MGMT	4	In-house (multi-center), public	T1, T1CE, T2, T2-FLAIR	CNNs	Radiomics	Python (Keras)	Not mentioned	Yes	38.64
Chang P, et al. (2018) [35]	259	MGMT	2, 3, 4	Public	T1, T1CE, T2, T2-FLAIR	CNNs	CNNs	Python (TensorFlow)	K-fold cross-validation	No	34.09
Han L, et al. (2018) [36]	262	MGMT	4	Public	T1, T2, T2-FLAIR	Not reported	Hybrid DL Model	Python (Not reported)	Held-out test set	Yes	38.64
Korfiatis P, et al. (2018) [37]	135	ATRX	2, 3, 4	Public	T2-FLAIR	Semi-automatic	CNNs	Python (TensorFlow)	K-fold cross-validation, held-out test set	No	36.36
Fukuma R, et al. (2019) [38]	164	TERT	2, 3	In-house (multi-center)	T1, T1CE, T2, T2-FLAIR	Manually	Hybrid (CNNs, Radiomics)	Python (Keras)	K-fold cross-validation	No	31.82
Tang Z, et al. (2020) [26]	120	MGMT, TERT	4	In-house (single-center)	T1CE, DWI	Manually	CNNs	Chainer, Python (PyRadiomics)	K-fold cross-validation	No	36.36
Crisi G, et al. (2020) [39]	59	MGMT	4	In-house (single-center)	DSC	Manually	Radiomics	Python (Not reported)	K-fold cross-validation	No	31.82
Hedyehzadeh M, et al. (2020) [40]	198	EGFR	4	Public	T1, T1CE, T2-FLAIR	CNNs	CNNs	WEKA, Python (LIFEx)	K-fold cross-validation, held-out test set	No	34.09
Calabrese E, et al. (2020) [27]	199	7/10 aneuploidy, MGMT, ATRX, EGFR, TERT, PTEN, CDKN2A/B, p53	4	In-house (single-center)	T1, T1CE, T2, T2-FLAIR, SWI, DWI, ASL, 2D 55-direction HARDI	CNNs	Radiomics	Python (PyTorch)	K-fold cross-validation	Yes	40.91
Chen X, et al. (2020) [41]	106	MGMT	4	Public	T1CE, T2-FLAIR	CNNs	CNNs	Python (TensorFlow, PyRadiomics)	K-fold cross-validation	No	34.09
Jonnalagedda P, et al. (2020) [42]	190	19/20 co-gain	4	In-house (single-center), public	T2-FLAIR	Manually	Hybrid DL Model	Python (Keras, scikit-learn)	K-fold cross-validation	Yes	36.36
Haubold J, et al. (2021) [28]	217	MGMT, ATRX	2, 3, 4	In-house (single-center)	T1, T1CE, T2-FLAIR	CNNs	Radiomics	Python (Not reported)	Held-out test set	No	34.09
Yogananda CGB, et al. (2021) [43]	247	MGMT	2, 3, 4	Public	T2	CNNs	CNNs	Python, DeepMedic (PyRadiomics)	K-fold cross-validation	No	38.64
Tupe-Waghmare P, et al. (2021) [44]	307	MGMT	4	In-house (multi-center), public	T1CE, T2, T2-FLAIR	CNNs	Hybrid DL Model	Python (Keras)	Held-out test set	Yes	40.91
Chen H, et al. (2021) [45]	244	PTEN	2, 3, 4	In-house (single-center), public	T1, T1CE, T2, T2-FLAIR	CNNs	Hybrid (CNNs, Radiomics)	Python (Not reported)	bootstrapping	Yes	47.73
Lang DM, et al. (2021) [46]	585	MGMT	4	Public	T1, T1CE, T2, T2-FLAIR	Not reported	C3D Network	Python (PyTorch, scikit-learn, PyRadiomics)	Held-out test set, bootstrapping	Yes	38.64
Xiao Z, et al. (2021) [47]	108	SYP	2, 3	Public	T1, T1CE, T2	Manually	CNNs	Python (Not reported)	K-fold cross-validation	No	43.18
Sohn B, et al. (2021) [29]	418	MGMT, ATRX, EGFR	4	In-house (single-center)	T1, T1CE, T2, T2-FLAIR	CNNs	Radiomics	Python (PyTorch)	Held-out test set	No	31.82
Capuozzo S, et al. (2022) [48]	864	MGMT	4	In-house (single-center), public	T1, T1CE, T2, T2-FLAIR	Knowledge-based filtering	CNNs	Python (Scikit-Learn, PyRadiomics)	K-fold cross-validation	Yes	43.18
Chen S, et al. (2022) [49]	111	MGMT	2, 3, 4	In-house (single-center)	T1CE, ADC	Manually	CNNs	Python (PyTorch)	K-fold cross-validation	No	31.82
Calabrese E, et al. (2022) [30]	400	7/10 aneuploidy, MGMT, ATRX, EGFR, TERT, PTEN, CDKN2A/B, p53	4	In-house (single-center), public	T1, T1CE, T2, T2-FLAIR, SWI, ASL	CNNs	Hybrid (CNNs, Radiomics)	Python (PyTorch)	K-fold cross-validation	No	38.64
Farzana W, et al. (2022) [50]	672	MGMT	4	Public	T1, T1CE, T2, T2-FLAIR	Not undertaken	CNNs	Python (TensorFlow, scikit-learn)	Not mentioned	Yes	34.09
Kim BH, et al. (2022) [51]	985	MGMT	2, 3, 4	In-house (single-center), public	T1, T1CE, T2, T2-FLAIR	Not reported	CNNs	Python (Not reported)	Held-out test set	Yes	45.45
Nalawade SS, et al. (2022) [52]	829	MGMT	2, 3, 4	Public	T2	Not reported	CNNs	Python (PyTorch, MONAI, scikit-learn)	K-fold cross-validation	No	31.82
Xu Q, et al. (2022) [31]	188	MGMT, p53, Ki67	2, 3, 4	In-house (single-center)	T1CE, T2	Not reported	Transformers and Attention Mechanisms	Python (Keras)	K-fold cross-validation, held-out test set	No	31.82
Spoorthy KR, et al. (2022) [53]	585	MGMT	4	Public	T1, T1CE, T2, T2-FLAIR	Not reported	CNNs	Python (Keras)	Not mentioned	Yes	36.36
Kihira S, et al. (2022) [54]	239	MGMT	2, 3, 4	In-house (multi-center)	T2-FLAIR	CNNs	Radiomics	Python (Keras)	K-fold cross-validation	Yes	31.82
Chaddad A, et al. (2023) [32]	83	ATRX, p53	2, 3	Public	T1, T2	Semi-automatic	Hybrid (CNNs, Radiomics)	Python (Not reported)	leave-one-out cross-validation, held-out test set	No	50.00
Faghani S, et al. (2023) [55]	576	MGMT	4	Public	T2	Not undertaken	CNNs	MATLAB	K-fold cross-validation	No	38.64
Chu W, et al. (2023) [56]	200	Ki67	2, 3, 4	In-house (single-center)	T1, T1CE, T2, T2-FLAIR	U-Net	CNNs	Python (PyTorch, MONAI, scikit-learn)	Held-out test set	No	27.27
Rui W, et al. (2023) [57]	42	ATRX	2, 3, 4	In-house (single-center)	T1CE, T2-FLAIR, QSM	Semi-automatic	CNNs	Python (PyTorch)	K-fold cross-validation, held-out test set	No	34.09
Saeed N, et al. (2023) [58]	585	MGMT	3, 4	Public	T1, T1CE, T2, T2-FLAIR	Manually	CNNs	Python (Not reported)	K-fold cross-validation	Yes	38.64
Sakly H, et al. (2023) [59]	985	MGMT	4	Public	T2-FLAIR	Not reported	CNNs	Python (PyTorch, MONAI)	K-fold cross-validation	Yes	43.18
Zhang H, et al. (2023) [60]	274	TERT	4	In-house (multi-center), public	T1, T1CE, T2	Manually	Hybrid (CNNs, Radiomics)	MATLAB	K-fold cross-validation	Yes	47.73
Saxena S, et al. (2023) [61]	585	MGMT	4	Public	T1	Manually	Hybrid DL Model	Python (Not reported, PyRadiomics)	K-fold cross-validation	Yes	40.91
Saxena S, et al. (2023) [62]	555	MGMT	4	Public	T1, T1CE, T2, T2-FLAIR	Manually	Hybrid (CNNs, Radiomics)	Python (PyTorch)	K-fold cross-validation	Yes	50.00
Robinet, et al. (2023) [13]	574	MGMT	4	In-house (single-center), public	T1CE, T2-FLAIR	CNNs	CNNs	Python (Not reported)	Held-out test set	Yes	43.18
Buz-Yalug B, et al. (2024) [63]	162	TERT	2, 3, 4	In-house (single-center)	T1, T1CE, DSC	Semi-automatic	Hybrid DL Model	Python (PyTorch)	K-fold cross-validation, held-out test set	No	29.55
Liu Z, et al. (2024) [64]	234	ATRX	3, 4	In-house (multi-center)	T1CE, T2-FLAIR	Semi-automatic	Hybrid (CNNs, Radiomics)	Python (TensorFlow, Keras)	K-fold cross-validation, held-out test set	Yes	45.45
Zhang L, et al. (2024) [65]	234	CDKN2A/B	2, 3	Public	T1CE, T2	Semi-automatic	Hybrid DL model	Python (PyTorch)	K-fold cross-validation, held-out test set	No	38.64
Zhang H, et al. (2024) [66]	229	TERT	4	In-house (multi-center)	T1, T1CE, T2	Manually	CNNs	Python (PyTorch)	K-fold cross-validation	Yes	34.09
Chen X, et al. (2023) [25]	161	MGMT	2, 3, 4	In-house (single-center)	T1CE, T2, T2-FLAIR	Manually	CNNs	Python (scikit-learn, PyRadiomics)	Held-out test set	No	25

**Table 2:** Sensitivity and specificity for deep learning models in the prediction of gliomas’ molecular markers. Each entry details the number of studies, sensitivity, and specificity with a 95% CI, PI with 95% CI, and *p*-value for both training (if available) and validation datasets. Abbreviations: No. of Studies, number of studies; MGMT, O6-methylguanine-DNA methyltransferase; ATRX, alpha thalassemia/mental retardation syndrome X-linked; TERT, telomerase reverse transcriptase; CDKN2A/B, CDKN2A/B homozygous deletion; SYP, Synaptophysin gene expression; EGFR, epidermal growth factor receptor; PTEN, phosphatase and tensin homolog; CI, confidence interval; AUC, area under the curve; PI, prediction internal. * Sensitivity and specificity ranges were reported for molecular markers with an inadequate number of studies (<5).

Gene	Dataset	No. of Studies	Sensitivity (95% CI)	PI (95% CI)	I^2^	*p*-Value	Specificity (95% CI)	PI (95% CI)	I^2^	*p*-Value	AUC
MGMT	Training	7	0.83 [0.72; 0.90]	[0.39; 0.97]	85.30%	0.00	0.79 [0.68; 0.88]	[0.33; 0.97]	83.6%	0.00	0.88
	Validation	23	0.74 [0.66; 0.80]	[0.35; 0.94]	80.90%	0.00	0.75 [0.65; 0.82]	[0.27; 0.96]	84.5%	0.00	0.81
ATRX	Validation	7	0.79 [0.67; 0.87]	[0.64; 0.89]	0.00%	0.79	0.85 [0.78; 0.91]	[0.62; 0.95]	40.7%	0.12	0.87
TERT	Validation	6	0.81 [0.72; 0.87]	[0.51; 0.94]	60.20%	0.03	0.70 [0.61; 0.77]	[0.46; 0.86]	40.0%	0.14	0.81
7/10 aneuploidy *	Validation	2	0.82–0.89	-	-	-	0.71–0.88	-	-	-	-
CDKN2A/B *	Validation	2	0.76–0.83	-	-	-	0.81–0.86	-	-	-	-
EGFR *	Validation	3	0.66–0.81	-	-	-	0.59–0.70	-	-	-	-
Ki67 *	Validation	1	0.98	-	-	-	0.81	-	-	-	-
p53 *	Validation	3	0.57–0.98	-	-	-	0.59–0.86	-	-	-	-
PTEN *	Validation	2	0.63–0.76	-	-	-	0.66–0.68	-	-	-	-
SYP *	Validation	1	0.90	-	-	-	0.95	-	-	-	-

**Table 3:** Subgroup analysis of heterogeneity via meta-regression for predicting MGMT methylation in validation cohorts. The table shows sensitivity and specificity with 95% confidence intervals (CI) across various covariates and subgroups. Abbreviations: No. of Studies, number of studies; CI, confidence interval; DL, deep learning; CNNs, convolutional neural networks; HGG, high-grade glioma; LGG, low-grade glioma.

Covariates	Subgroup	No. of Studies	Sensitivity (95% CI)	*p*-Value (Between Study)	Specificity (95% CI)	*p*-Value (Between Study)
Tumor grade	HGG	20	0.68 [0.63; 0.73]	0.05	0.71 [0.62; 0.78]	0.13
	LGG & HGG	9	0.85 [0.68; 0.94]		0.83 [0.68; 0.92]	
Clinical information	Included	9	0.71 [0.56; 0.83]	0.69	0.80 [0.67; 0.89]	0.25
	Not included	20	0.74 [0.67; 0.80]		0.71 [0.62; 0.79]	
Data augmentation	Included	18	0.77 [0.68; 0.85]	0.12	0.75 [0.64; 0.84]	0.81
	Not included	11	0.68 [0.61; 0.75]		0.73 [0.62; 0.82]	
Dataset	In-house (single center)	11	0.80 [0.67; 0.89]	0.01	0.87 [0.76; 0.93]	0.00
	In-house (single center), Public	6	0.61 [0.55; 0.67]		0.59 [0.49; 0.68]	
Segmentation method	DL	13	0.69 [0.58; 0.79]	0.26	0.77 [0.66; 0.85]	0.76
	Manually	6	0.76 [0.70; 0.81]		0.79 [0.66; 0.88]	
Feature extraction	CNNs	12	0.81 [0.71; 0.89]	0.00	0.74 [0.57; 0.86]	0.70
	Radiomics	6	0.56 [0.50; 0.62]		0.78 [0.59; 0.90]	
Pretrained model	Employed	11	0.73 [0.60; 0.84]	0.94	0.64 [0.51; 0.75]	0.03
	Not employed	18	0.73 [0.65; 0.80]		0.80 [0.71; 0.87]	
DL integration	Feature extraction	20	0.79 [0.71; 0.85]	0.00	0.72 [0.63; 0.79]	0.68
	Tumor Segmentation	6	0.56 [0.50; 0.62]		0.78 [0.59; 0.90]	
	Classification	3	0.72 [0.47; 0.88]		0.81 [0.47; 0.95]	
No. of MRI sequences	One Sequence	15	0.83 [0.71; 0.91]	0.03	0.75 [0.61; 0.85]	0.54
	Four Sequences	7	0.66 [0.53; 0.76]		0.82 [0.60; 0.93]	
MRI technique	Conventional	10	0.70 [0.60; 0.79]	0.44	0.75 [0.62; 0.85]	0.90
	Advanced, Conventional	21	0.75 [0.66; 0.83]		0.74 [0.64; 0.82]	
Validation method	Internally Validated Only	18	0.66 [0.58; 0.73]	0.03	0.82 [0.74; 0.88]	0.00
	Both Internally and Externally Validated	10	0.79 [0.70; 0.86]		0.58 [0.50; 0.65]	
Internal validation	Held-out test set	7	0.60 [0.55; 0.65]	0.01	0.73 [0.48; 0.89]	0.91
	K-fold cross-validation	16	0.74 [0.65; 0.82]		0.71 [0.62; 0.79]	

## Data Availability

Not applicable.

## Supplementary Materials

The following supporting information can be downloaded at: https://www.mdpi.com/article/10.3390/diagnostics15070797/s1.

## Abbreviations

The following abbreviations are used in this manuscript:

MGMT	O-6-methylguanine-DNA methyltransferase
PTEN	Phosphatase and tensin homolog
ATRX	alpha-thalassemia/mental retardation syndrome X-linked
TERT	telomerase reverse transcriptase
CDKN2A/B	cyclin-dependent kinase inhibitor 2A/B
EGFR	epidermal growth factor receptor
SYP	Synaptophysin
QUADAS-2	Quality assessment of diagnostic accuracy studies-2
RQS	Radiomics quality score
