# Supplementary material for: Diagnostic Accuracy of Deep Learning Models in Predicting Glioma Molecular Markers: A Systematic Review and Meta-Analysis
Source: Diagnostics (Basel). 2025 Mar 21;15(7):797. doi: 10.3390/diagnostics15070797 (PMC11988998; doi:10.3390/diagnostics15070797)
Supplement: Supplementary file 1 [file diagnostics-15-00797-s001.zip › diagnostics-3506815-supplementary.pdf]

# Supplementary Materials

Diagnostic Accuracy of Deep Learning Models in Predicting Glioma Molecular Markers: A Systematic Review and Meta-Analysis

## Contents


1. Search Strategy ..... 4
2. Data Extraction Form ..... 6
3. QUADAS-2 ..... 7
    3.1. QUADAS-2 Questions ..... 7
    3.2. QUADAS-2 Results ..... 8
4. Radiomics Quality Score (RQS) ..... 10
    4.1. RQS Domains ..... 10
    4.2. RQS Results ..... 11
5. Sensitivity analysis ..... 13
    5.1. MGMT mutation Prediction in Validation Cohorts ..... 13
        5.1.1. Pooled sensitivity estimate ..... 13
        5.1.2. Pooled specificity estimate ..... 13
    5.2. MGMT mutation Prediction in Training Cohorts ..... 14
        5.2.1. Pooled sensitivity estimate ..... 14
        5.2.2. Pooled specificity estimate ..... 14
    5.3. ATRX Prediction in Validation Cohorts ..... 14
        5.3.1. Pooled sensitivity estimate ..... 14
        5.3.2. Pooled specificity estimate ..... 14
    5.4. TERT Prediction in Validation Cohorts ..... 14
        5.4.1. Pooled sensitivity estimate ..... 14
        5.4.2. Pooled specificity estimate ..... 14
6. Publication Bias ..... 15
    6.1. MGMT mutation Prediction in Validation Cohorts ..... 15
    6.1.1. MGMT mutation Prediction in Validation Cohorts (Trim and Fill method) ..... 16
    6.2. MGMT mutation Prediction in Training Cohorts ..... 17
    6.3. ATRX Prediction in Validation Cohorts ..... 18
    6.4. TERT Prediction in Validation Cohorts ..... 19
7. Forest plot ..... 20
    7.1. MGM Prediction in Training Cohorts: Pooled sensitivity estimate ..... 20
    7.2. MGM Prediction in Training Cohorts: Pooled specificity estimate ..... 21
8. Cross-Hair Plot ..... 22
    8.1. MGMT Prediction in Validation Cohorts ..... 22

8.2. MGMT Prediction in Training Cohorts .......... 23
8.3. ATRX Prediction in Validation Cohorts .......... 24
8.4. TERT Prediction in Validation Cohorts .......... 25
9. Statistical Power .......... 25
9.1. MGMT Prediction in Validation Cohorts .......... 26
9.1.1. Pooled sensitivity estimate .......... 26
9.1.2. Pooled specificity estimate .......... 27
9.2. MGMT Prediction in Training Cohorts .......... 28
9.2.1. Pooled sensitivity estimate .......... 28
9.2.2. Pooled specificity estimate .......... 28
9.3. ATRX Prediction in Validation Cohorts .......... 30
9.3.1. Pooled sensitivity estimate .......... 30
9.3.2. Pooled specificity estimate .......... 31
9.4. TERT Prediction in Validation Cohorts .......... 32
9.4.1. Pooled sensitivity estimate .......... 32
9.4.2. Pooled specificity estimate .......... 33
10. Meta-Regression Analysis of MGMT Prediction in Validation Cohorts .......... 34
10.1. Tumor Grade .......... 34
10.1.1. SROC curves .......... 34
10.1.2. Accuracy vs. Covariate Plot .......... 35
10.2. Clinical Information .......... 36
10.2.1. SROC curves .......... 36
10.2.2. Accuracy vs. Covariate Plot .......... 37
10.3. Data Augmentation .......... 38
10.3.1. SROC curves .......... 38
10.3.2. Accuracy vs. Covariate Plot .......... 39
10.4. Dataset .......... 40
10.4.1. SROC curves .......... 40
10.4.2. Accuracy vs. Covariate Plot .......... 41
10.5. Segmentation Method .......... 42
10.5.1. SROC curves .......... 42
10.5.2. Accuracy vs. Covariate Plot .......... 43
10.6. Feature Extraction .......... 44
10.6.1. SROC curves .......... 44
10.6.2. Accuracy vs. Covariate Plot .......... 45

10.7. Pretrained Model ........ 46
10.7.1. SROC curves ........ 46
10.7.2. Accuracy vs. Covariate Plot ........ 47
10.8. Deep Learning Integration Level ........ 48
10.8.1. SROC curves ........ 48
10.8.2. Accuracy vs. Covariate Plot ........ 49
10.9. MRI ........ 50
10.9.1. SROC curves ........ 50
10.9.2. Accuracy vs. Covariate Plot ........ 51
10.10. MRI Sequences ........ 52
10.10.1. SROC curves ........ 52
10.10.2. Accuracy vs. Covariate Plot ........ 53
10.11. Validation Method ........ 54
10.11.1. SROC curves ........ 54
10.11.2. Accuracy vs. Covariate Plot ........ 55
10.12. Internal Validation ........ 56
10.12.1. SROC curves ........ 56
10.12.2. Accuracy vs. Covariate Plot ........ 57
10.13. RQS ........ 58
10.13.1. SROC curves ........ 58
10.13.2. Accuracy vs. Covariate Plot ........ 59
11. MRI Sequences ........ 60
13. References ........ 61

# 1. Search Strategy

(27-2-2024)

## 1.1.PubMed: [78 results]

("Genetic Markers"[Mesh] OR "Biomarkers, Tumor"[Mesh] OR "Biomarkers"[Mesh] OR "Mutation/genetics"[Mesh] OR "Imaging Genomics"[Mesh] OR “Radiogenomics” OR “Radio-genomics” OR "TERT protein, human" [Supplementary Concept] OR "telomerase reverse transcriptase (540-548)" [Supplementary Concept] OR "telomerase reverse transcriptase p572Y, human" [Supplementary Concept] OR "O(6)-Methylguanine-DNA Methyltransferase"[Mesh] OR "Genes, p53"[Mesh] OR "Tumor Suppressor Protein p53"[Mesh] OR "Genes, p53"[Mesh] OR "Tumor Suppressor Protein p53"[Mesh] OR ("EGFR protein, human" [Supplementary Concept] OR "ERBB2 protein, human" [Supplementary Concept] OR "PTEN protein, human" [Supplementary Concept] OR "ATRX protein, human" [Supplementary Concept] OR "ATR-X syndrome" [Supplementary Concept] OR "Chromosome 7, trisomy 7q" [Supplementary Concept] OR "Chromosome 7, trisomy 7p" [Supplementary Concept] OR ("Chromosome 10, monosomy 10q" [Supplementary Concept]) OR "Distal Trisomy 10q Syndrome" [Supplementary Concept] OR "CIC protein, human" [Supplementary Concept] OR "Receptor, Platelet-Derived Growth Factor alpha"[Mesh] OR "CDKN2A protein, human" [Supplementary Concept] OR "Genes, p16"[Mesh] OR "Cyclin-Dependent Kinase Inhibitor p16"[Mesh]) AND ("Artificial Intelligence"[Mesh] OR "Deep Learning"[Title/Abstract] OR "Machine Learning"[Title/Abstract] OR "Unsupervised Machine Learning"[Title/Abstract] OR "Supervised Machine Learning"[Title/Abstract]) AND ("Glioblastoma"[Title/Abstract] OR "Glioma"[Title/Abstract] OR "Glioma, Subependymal"[Title/Abstract] OR "Astrocytoma"[Title/Abstract] OR "Retinoblastoma"[Title/Abstract] OR "Brain Neoplasms"[Title/Abstract]) AND ("Magnetic Resonance Imaging"[Mesh] OR "Diffusion Tensor Imaging"[Mesh] OR "Diffusion Magnetic Resonance Imaging"[Mesh] OR "Multiparametric Magnetic Resonance Imaging"[Mesh] OR "Magnetic Resonance Imaging"[Mesh])

## 1.2.Scopus: [337 results]

TITLE-ABS-KEY (("Genetic Markers" OR "Biomarkers, Tumor" OR "Biomarkers" OR "Mutation/genetics" OR "Imaging Genomics" OR "Radiogenomics" OR "Radio-genomics" OR "TERT protein, human" OR "telomerase reverse transcriptase" OR "telomerase reverse transcriptase p572Y, human" OR "O(6)-Methylguanine-DNA Methyltransferase" OR "Genes, p53" OR "Tumor Suppressor Protein p53" OR "TP53 protein" OR "Genes, p53" OR "Tumor Suppressor Protein p53" OR "TP53 protein, human" OR "EGFR protein, human" OR "ERBB2 protein, human" OR "PTEN protein, human" OR "ATRX protein, human" OR "ATR-X syndrome" OR "Chromosome 7, trisomy 7q" OR "Chromosome 7, trisomy 7p" OR "Chromosome 10, monosomy 10q" OR "Distal Trisomy 10q Syndrome" OR "CIC protein, human" OR "Receptor, Platelet-Derived Growth Factor alpha" OR "CDKN2A protein, human" OR "Genes, p16" OR "Cyclin-Dependent Kinase Inhibitor p16")) AND (TITLE-ABS-KEY (("Artificial Intelligence" OR "Deep Learning" OR "Machine Learning" OR "Unsupervised Machine Learning" OR "Supervised Machine Learning") AND ("Glioblastoma" OR "Glioma" OR "Glioma, Subependymal" OR "Astrocytoma" OR "Retinoblastoma" OR "Brain Neoplasms") AND ("Magnetic Resonance Imaging" OR "Diffusion Tensor Imaging" OR "Diffusion Magnetic Resonance Imaging" OR "Multiparametric Magnetic Resonance Imaging" OR "Magnetic Resonance Imaging")))

## 1.3.Web of Science: [288 results]

TS=(("Genetic Markers" OR "Biomarkers, Tumor" OR "Biomarkers" OR "Mutation/genetics" OR "Imaging Genomics" OR "Radiogenomics" OR "Radio-genomics" OR "TERT protein, human" OR "telomerase reverse

transcriptase" OR "telomerase reverse transcriptase p572Y, human" OR "O(6)-Methylguanine-DNA Methyltransferase" OR "Genes, p53" OR "Tumor Suppressor Protein p53" OR "TP53 protein" OR "Genes, p53" OR "Tumor Suppressor Protein p53" OR "TP53 protein, human" OR "EGFR protein, human" OR "ERBB2 protein, human" OR "PTEN protein, human" OR "ATRX protein, human" OR "ATR-X syndrome" OR "Chromosome 7, trisomy 7q" OR "Chromosome 7, trisomy 7p" OR "Chromosome 10, monosomy 10q" OR "Distal Trisomy 10q Syndrome" OR "CIC protein, human" OR "Receptor, Platelet-Derived Growth Factor alpha" OR "CDKN2A protein, human" OR "Genes, p16" OR "Cyclin-Dependent Kinase Inhibitor p16") AND ("Artificial Intelligence" OR "Deep Learning" OR "Machine Learning" OR "Unsupervised Machine Learning" OR "Supervised Machine Learning") AND ("Glioblastoma" OR "Glioma" OR "Glioma, Subependymal" OR "Astrocytoma" OR "Retinoblastoma" OR "Brain Neoplasms") AND ("Magnetic Resonance Imaging" OR "Diffusion Tensor Imaging" OR "Diffusion Magnetic Resonance Imaging" OR "Multiparametric Magnetic Resonance Imaging" OR "Magnetic Resonance Imaging"))

### 1.4. Ovid: [321 results]

("Genetic Markers.ti,ab,kf. or Biomarkers, Tumor.ti,ab,kf. or Biomarkers.ti,ab,kf. or Mutation/genetics.ti,ab,kf. or Imaging Genomics.ti,ab,kf. or transcriptase.ti,ab,kf. or telomerase reverse transcriptase p572Y, human.ti,ab,kf. or O(6)-Methylguanine-DNA Methyltransferase.ti,ab,kf. or Genes, p53.ti,ab,kf. or Tumor Suppressor Protein p53.ti,ab,kf. or TP53 protein.ti,ab,kf. or Genes, p53.ti,ab,kf. or Tumor Suppressor Protein p53.ti,ab,kf. or TP53 protein, human.ti,ab,kf. or EGFR protein, human.ti,ab,kf. or ERBB2 protein, human.ti,ab,kf. or PTEN protein, human.ti,ab,kf. or ATRX protein, human.ti,ab,kf. or ATR-X syndrome.ti,ab,kf. or Chromosome 7, trisomy 7q.ti,ab,kf. or Chromosome 7, trisomy 7p.ti,ab,kf. or Chromosome 10, monosomy 10q.ti,ab,kf. or Distal Trisomy 10q Syndrome.ti,ab,kf. or CIC protein, human.ti,ab,kf. or Receptor, Platelet-Derived Growth Factor alpha.ti,ab,kf. or CDKN2A protein, human.ti,ab,kf. or Genes, p16.ti,ab,kf. or Cyclin-Dependent Kinase Inhibitor p16") and ("Artificial Intelligence.ti,ab,kf. or Deep Learning.ti,ab,kf. or Machine Learning.ti,ab,kf. or Unsupervised Machine Learning.ti,ab,kf. or Supervised Machine Learning") and ("Glioblastoma.ti,ab,kf. or Glioma.ti,ab,kf. or Glioma, Subependymal.ti,ab,kf. or Astrocytoma.ti,ab,kf. or Retinoblastoma.ti,ab,kf. or Brain Neoplasms") and ("Magnetic Resonance Imaging.ti,ab,kf. or Diffusion Tensor Imaging.ti,ab,kf. or Diffusion Magnetic Resonance Imaging.ti,ab,kf. or Multiparametric Magnetic Resonance Imaging.ti,ab,kf. or Magnetic Resonance Imaging")

## 2. Data Extraction Form

**Table S1.** Template data collection form. Abbreviations: AUC for Area Under the Curve, ACC for Accuracy.

| | **Source** | | | | | **Eligibility** | | **Methods** | | | **Patients** | | | | | | | | | | | **Index test** | | | | | | | | | | | | | | | | | | |
|---|---|---|---|---|---|---|---|---|---|---|---|---|---|---|---|---|---|---|---|---|---|---|---|---|---|---|---|---|---|---|---|---|---|---|---|---|---|---|---|---|
| Number | Study ID | Report ID | Sources of data (Journal articles, Conference articles, …) | Year | Country | Eligibility for review | Reason for exclusion | Study design | Dataset (single- or multi-center) | Objective | No. of patients (train/test/external validation) | Clinical data inclusion (age, sex, overall survival, …) | Genes | Reference standard | Glioma Grade | Phantom study on all scanners | Imaging at multiple time points | Image protocol quality | MRI | MRI Techniques (Conventional vs. Advanced) | Number of MRI Sequences | Pre-trained Model | Data Augmentation | Segmentation Methods | Manual or Semi-automatic Segmentation: Expert Count | Feature Extraction Method | Cut-off analyses | Calibration statistics | Model Classification | Level of DL Integration | Internal Validation | External Validation | External Validation: Number of Institutions | Performance Metrics (AUC, ACC, Sensitivity, Specificity, | Comparison to " gold standard" | Detect and discuss biological correlates | Potential clinical utility | Cost-effectiveness analysis | Data availability | Code availability |

# 3. QUADAS-2

## 3.1.QUADAS-2 Questions

### Risk of bias:

**Domain 1:** Patient Selection
1. Were imaging acquisition protocols clearly documented for each dataset? (Yes/No/Unclear)
2. If a portion of the dataset was selected, was it done randomly? (Yes/No/Unclear)
3. Did the study avoid inappropriate exclusions? (Yes/No/Unclear)
4. Did the study avoid introducing bias by employing appropriate methods to handle missing data? (Yes/No/Unclear)

**Domain 2:** Index Test
1. Were multiple segmentations performed for manual or semi-automatic methods, or was deep learning-based segmentation utilized? (Yes/No/Unclear)
2. Was a predetermined threshold applied for model predictions? (Yes/No/Unclear)
3. Are discrimination statistics and their significance reported? (Yes/No/Unclear)

**Domain 3:** Reference Standard
1. Is the reference standard reliable for classifying the target condition? (Yes/No/Unclear)
2. Were severe genotype class imbalances avoided? (Yes/No/Unclear)

**Domain 4:** Flow & Timing
1. Did all patient groups (train, test, validation) undergo the reference standard? (Yes/No/Unclear)
2. Was the same reference standard applied to all patients? (Yes/No/Unclear)

### Applicability Concerns

**Domain 1:** Patient Selection
Is there concern regarding patient inclusion matching the review question? (Low/High/Unclear)

**Domain 2**: Index Test
Was validation carried out using "unseen" data from an external cohort or a held-out test set? (Yes/No/Unclear)

**Domain 3:** Reference Standard
Does the target condition as defined by the reference standard align with the review question? (Yes/No/Unclear)

### Overall Assessment:

- The study is considered to have a "High" risk of bias if any single domain is rated as high.
- The study is considered to have an "Unclear" risk if two or more domains are rated as unclear.

(**Yes**: Low risk of bias, **No**: High risk of bias, **Unclear**: Some concerns)

## 3.2.QUADAS-2 Results

**Table S2**. QUADAS-2 assessment results for each study.

| | Risk of Bias | | | | | Applicability Concerns | | | |
|---|---|---|---|---|---|---|---|---|---|
| **Study** | D1 | D2 | D3 | D4 | **Overall** | D1 | D2 | D3 | **Overall** |
| Fukuma R, et al. (2019) | Low Risk | High Risk | Low Risk | Low Risk | High Risk | Low Risk | Low Risk | Low Risk | Low Risk |
| Korfiatis P, et al. (2017) | Low Risk | Low Risk | Low Risk | Low Risk | Low Risk | Low Risk | Low Risk | Low Risk | Low Risk |
| Li ZC, et al. (2018) | High Risk | High Risk | Low Risk | Low Risk | High Risk | Low Risk | Low Risk | Low Risk | Low Risk |
| Chang P, et al. (2018) | Low Risk | Low Risk | Low Risk | Low Risk | Low Risk | Low Risk | Low Risk | Low Risk | Low Risk |
| Tang Z, et al. (2020) | Low Risk | High Risk | Low Risk | Low Risk | High Risk | Low Risk | Low Risk | Low Risk | Low Risk |
| Haubold J, et al. (2021) | Low Risk | Low Risk | Low Risk | Low Risk | Low Risk | Low Risk | Low Risk | Low Risk | Low Risk |
| Crisi G, et al. (2020) | Low Risk | High Risk | Low Risk | Low Risk | High Risk | High Risk | Low Risk | Low Risk | Low Risk |
| Yogananda CGB, et al. (2021) | Low Risk | Low Risk | Low Risk | Low Risk | Low Risk | Low Risk | Low Risk | Low Risk | Low Risk |
| Tupe-Waghmare P, et al. (2021) | Low Risk | Low Risk | Low Risk | Low Risk | Low Risk | Low Risk | Low Risk | Low Risk | Low Risk |
| Han L, et al. (2018) | Low Risk | Low Risk | Low Risk | Low Risk | Low Risk | Low Risk | Low Risk | Low Risk | Low Risk |
| Hedyehzadeh M, et al. (2020) | Low Risk | Some Concerns | Low Risk | Low Risk | Low Risk | Low Risk | Low Risk | Low Risk | Low Risk |
| Calabrese E, et al. (2020) | Low Risk | Low Risk | Low Risk | Low Risk | Low Risk | Low Risk | Low Risk | Low Risk | Low Risk |
| Capuozzo S, et al. (2022) | Low Risk | Low Risk | Low Risk | Low Risk | Low Risk | Low Risk | Low Risk | Low Risk | Low Risk |
| Chaddad A, et al. (2023) | Low Risk | Low Risk | Low Risk | Low Risk | Low Risk | High Risk | Low Risk | Low Risk | Low Risk |
| Chen H, et al. (2021) | Low Risk | Low Risk | Low Risk | Low Risk | Low Risk | Low Risk | Low Risk | Low Risk | Low Risk |
| Chen S, et al. (2022) | Low Risk | Low Risk | Low Risk | Low Risk | Low Risk | Low Risk | Low Risk | Low Risk | Low Risk |
| Faghani S, et al. (2023) | Low Risk | Low Risk | Low Risk | Low Risk | Low Risk | Low Risk | Low Risk | Low Risk | Low Risk |
| Chu W, et al. (2023) | Low Risk | Low Risk | Low Risk | Low Risk | Low Risk | Low Risk | Low Risk | Low Risk | Low Risk |
| Buz-Yalug B, et al. (2024) | Low Risk | Low Risk | Low Risk | Low Risk | Low Risk | Low Risk | Low Risk | Low Risk | Low Risk |
| Calabrese E, et al. (2022) | Low Risk | Low Risk | Low Risk | Low Risk | Low Risk | Low Risk | Low Risk | Low Risk | Low Risk |
| Chen X, et al. (2020) | Low Risk | Low Risk | Low Risk | Low Risk | Low Risk | Low Risk | Low Risk | Low Risk | Low Risk |
| Farzana W, et al. (2022) | Low Risk | Low Risk | Low Risk | Low Risk | Low Risk | Low Risk | Low Risk | Low Risk | Low Risk |
| Jonnalagedda P, et al. (2020) | Low Risk | Low Risk | Low Risk | Low Risk | Low Risk | Low Risk | Low Risk | Low Risk | Low Risk |
| Kim BH, et al. (2022) | Low Risk | Low Risk | Low Risk | Low Risk | Low Risk | Low Risk | Low Risk | Low Risk | Low Risk |
| Korfiatis P, et al. (2018) | Low Risk | Some Concerns | Low Risk | Low Risk | Low Risk | Low Risk | Low Risk | Low Risk | Low Risk |
| Lang DM, et al. (2021) | Low Risk | Low Risk | Low Risk | Low Risk | Low Risk | Low Risk | Low Risk | Low Risk | Low Risk |
| Liu Z, et al. (2024) | Low Risk | Low Risk | Low Risk | Low Risk | Low Risk | Low Risk | Low Risk | Low Risk | Low Risk |
| Nalawade SS, et al. (2022) | Low Risk | Low Risk | Low Risk | Low Risk | Low Risk | Low Risk | Low Risk | Low Risk | Low Risk |

Low Risk ✓
High Risk ✗
Some Concerns !

**Table S2.** Continued.

| | Risk of Bias | | | | | Applicability Concerns | | | |
|---|---|---|---|---|---|---|---|---|---|
| | D1 | D2 | D3 | D4 | **Overall** | D1 | D2 | D3 | **Overall** |
| Rui W, et al. (2023) | ✓ | ✓ | ✓ | ✓ | ✓ | ✓ | ✓ | ✓ | ✓ |
| Saeed N, et al. (2023) | ✓ | ✓ | ✓ | ✓ | ✓ | ✓ | ✓ | ✓ | ✓ |
| Sakly H, et al. (2023) | ✓ | ✓ | ✓ | ✓ | ✓ | ✓ | ✓ | ✓ | ✓ |
| Zhang L, et al. (2024) | ✓ | ✓ | ✓ | ✓ | ✓ | ✓ | ✓ | ✓ | ✓ |
| Zhang H, et al. (2023) | ✓ | ✓ | ✓ | ✓ | ✓ | ✓ | ✓ | ✓ | ✓ |
| Zhang H, et al. (2024) | ✓ | ✓ | ✓ | ✓ | ✓ | ✓ | ✓ | ✓ | ✓ |
| Xu Q, et al. (2022) | ✓ | ✓ | ✓ | ✓ | ✓ | ✗ | ✓ | ✓ | ✓ |
| Xiao Z, et al. (2021) | ✓ | ✓ | ✓ | ✓ | ✓ | ✗ | ✓ | ✓ | ✓ |
| Saxena S, et al. (2023) | ✓ | ✓ | ✓ | ✓ | ✓ | ✓ | ✓ | ✓ | ✓ |
| Saxena S, et al. (2023) | ✓ | ✓ | ✓ | ✓ | ✓ | ✓ | ✓ | ✓ | ✓ |
| Spoorthy KR, et al. (2022) | ✓ | ✗ | ✓ | ✓ | ✓ | ✓ | ✓ | ✓ | ✓ |
| Kihira S, et al. (2022) | ✓ | ✓ | ✓ | ✓ | ✓ | ✗ | ✓ | ✓ | ✓ |
| Sohn B, et al. (2021) | ✓ | ✓ | ✓ | ✓ | ✓ | ✓ | ✓ | ✓ | ✓ |
| Robinet, et al. (2023) | ✓ | ✓ | ✓ | ✓ | ✓ | ✓ | ✓ | ✓ | ✓ |
| Chen X, et al. (2023) | ✓ | ✓ | ✓ | ✓ | ✓ | ✓ | ✓ | ✓ | ✓ |

Low Risk ✓
High Risk ✗
Some Concerns !

# 4. Radiomics Quality Score (RQS)

## 4.1. RQS Domains

- **Domain 1**—Protocol quality and stability in image and segmentation (0–5 points)
- **Domain 2**—Feature selection and validation (−8 to 8 points)
- **Domain 3**—Biologic/clinical validation and utility (0–6 points)
- **Domain 4**—Model performance index (0–5 points)
- **Domain 5**—High level of evidence (0–8 points)
- **Domain 6**—Open science and data (0–4 points)

## 4.2. RQS Results

**Table S3.** Heatmap of Radiomics Quality Scores (RQSs) for included articles.

| Study | Domain 1 (%) | Domain 2 (%) | Domain 3 (%) | Domain 4 (%) | Domain 5 (%) | Domain 6 (%) |
|---|---|---|---|---|---|---|
| Fukuma R, et al. (2019) | | | | | | |
| Korfiatis P, et al. (2017) | | | | | | |
| Li ZC, et al. (2018) | | | | | | |
| Chang P, et al. (2018) | | | | | | |
| Tang Z, et al. (2020) | | | | | | |
| Haubold J, et al. (2021) | | | | | | |
| Crisi G, et al. (2020) | | | | | | |
| Yogananda CGB, et al. (2021) | | | | | | |
| Tupe-Waghmare P, et al. (2021) | | | | | | |
| Han L, et al. (2018) | | | | | | |
| Hedyehzadeh M, et al. (2020) | | | | | | |
| Calabrese E, et al. (2020) | | | | | | |
| Capuozzo S, et al. (2022) | | | | | | |
| Chaddad A, et al. (2023) | | | | | | |
| Chen H, et al. (2021) | | | | | | |
| Chen S, et al. (2022) | | | | | | |
| Faghani S, et al. (2023) | | | | | | |
| Chu W, et al. (2023) | | | | | | |
| Buz-Yalug B, et al. (2024) | | | | | | |
| Calabrese E, et al. (2022) | | | | | | |
| Chen X, et al. (2020) | | | | | | |
| Farzana W, et al. (2022) | | | | | | |
| Jonnalagedda P, et al. (2020) | | | | | | |
| Kim BH, et al. (2022) | | | | | | |
| Korfiatis P, et al. (2018) | | | | | | |
| Lang DM, et al. (2021) | | | | | | |
| Liu Z, et al. (2024) | | | | | | |
| Nalawade SS, et al. (2022) | | | | | | |
| Rui W, et al. (2023) | | | | | | |

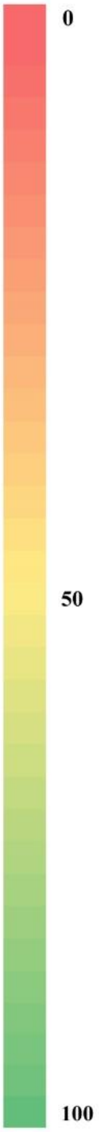

**Table S3.** Continued.

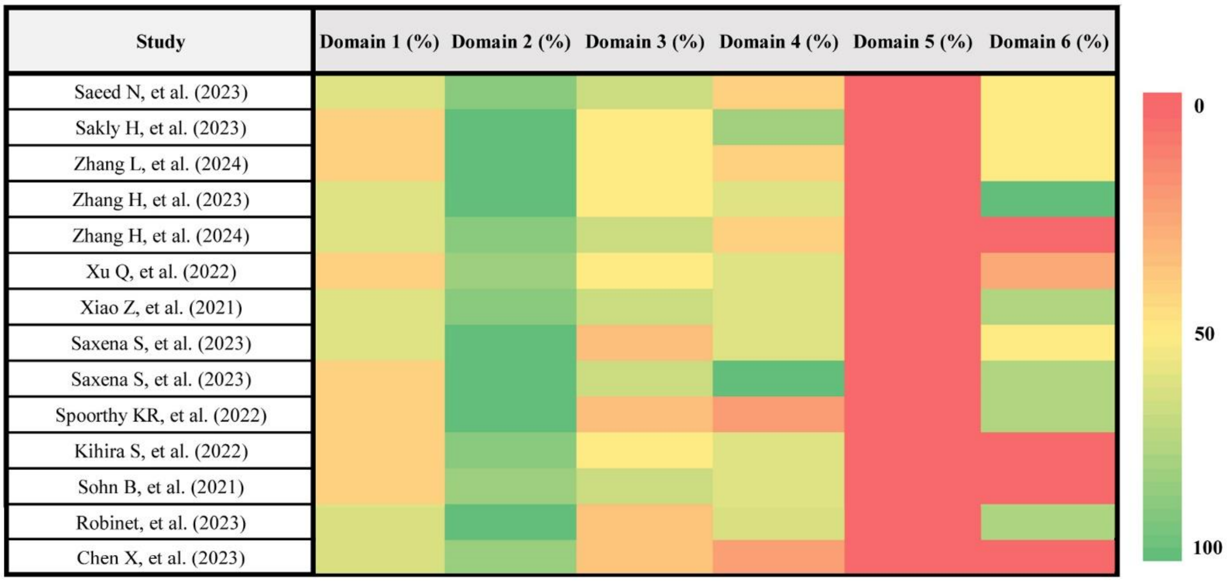

# 5. Sensitivity analysis

## 5.1. MGMT mutation Prediction in Validation Cohorts

### 5.1.1. Pooled sensitivity estimate

**Identified outliers (random-effects model):**

"Sakly H, et al. (2023)", "Yogananda CGB, et al. (2021)", "Sohn B, et al. (2021)", "Capuozzo S, et al. (2022)", "Calabrese E, et al. (2020)"

**Results with outliers removed:**

Number of studies: k = 18
Number of observations: o = 1102
Number of events: e = 766

| | **proportion** | **95%-CI** |
|---|---|---|
| Random effects model | 0.7096 | [0.6592; 0.7552] |
| Prediction interval | | [0.5415; 0.8348] |

**Quantifying heterogeneity:**
tau^2 = 0.1034 [0.0246; 0.9624]; tau = 0.3215 [0.1568; 0.9810]
I^2 = 54.3% [22.2%; 73.2%]; H = 1.48 [1.13; 1.93]

**Test of heterogeneity:**

| Q | d.f | .p-value |
|---|---|---|
| 37.23 | 17 | 0.0031 |

### 5.1.2. Pooled specificity estimate

**Identified outliers (random-effects model):**
"Sakly H, et al. (2023)", "Yogananda CGB, et al. (2021)", "Chen S, et al. (2022)", "Robinet, et al. (2023)", "Sohn B, et al. (20 21)", "Capuozzo S, et al. (2022)", "Calabrese E, et al. (2020)", "Kim BH, et al. (2022)"

**Results with outliers removed:**

Number of studies: k = 15
Number of observations: o = 793
Number of events: e = 551

| | **proportion** | **95%-CI** |
|---|---|---|
| Random effects model | 0.7475 | [0.6775; 0.8066] |
| Prediction interval | | [0.4933; 0.9000] |

**Quantifying heterogeneity:**
tau^2 = 0.2343 [0.0514; 1.2144]; tau = 0.4840 [0.2268; 1.1020]
I^2 = 63.7% [36.9%; 79.1%]; H = 1.66 [1.26; 2.19]

**Test of heterogeneity:**

| Q | d.f. | p-value |
|---|---|---|
| 38.59 | 14 | 0.0004 |

## 5.2. MGMT mutation Prediction in Training Cohorts

### 5.2.1. Pooled sensitivity estimate

**Identified outliers (random-effects model)**
"Kihira S, et al. (2022)"

**Results with outliers removed**
Number of studies: k = 6
Number of observations: o = 459
Number of events: e = 398

| | proportion | 95%-CI |
|---|---|---|
| Random effects model | 0.8547 | [0.7733; 0.9103] |
| Prediction interval | | [0.5235; 0.9692] |

**Quantifying heterogeneity:**
tau^2 = 0.2879 [0.0265; 3.9177]; tau = 0.5366 [0.1628; 1.9793]
I^2 = 68.1% [24.4%; 86.5%]; H = 1.77 [1.15; 2.72]

**Test of heterogeneity:**

| Q | d.f. | p-value |
|---|---|---|
| 15.67 | 5 | 0.0079 |

### 5.2.2. Pooled specificity estimate

**Identified outliers (random-effects model):**
No outliers detected (random-effects model).

## 5.3. ATRX Prediction in Validation Cohorts

### 5.3.1. Pooled sensitivity estimate

**Identified outliers (random-effects model):**
No outliers detected (random-effects model).

### 5.3.2. Pooled specificity estimate

**Identified outliers (random-effects model):**
No outliers detected (random-effects model).

## 5.4. TERT Prediction in Validation Cohorts

### 5.4.1. Pooled sensitivity estimate

**Identified outliers (random-effects model):**
No outliers detected (random-effects model).

### 5.4.2. Pooled specificity estimate

No outliers detected (random-effects model).

# 6. Publication Bias

## 6.1. MGMT mutation Prediction in Validation Cohorts

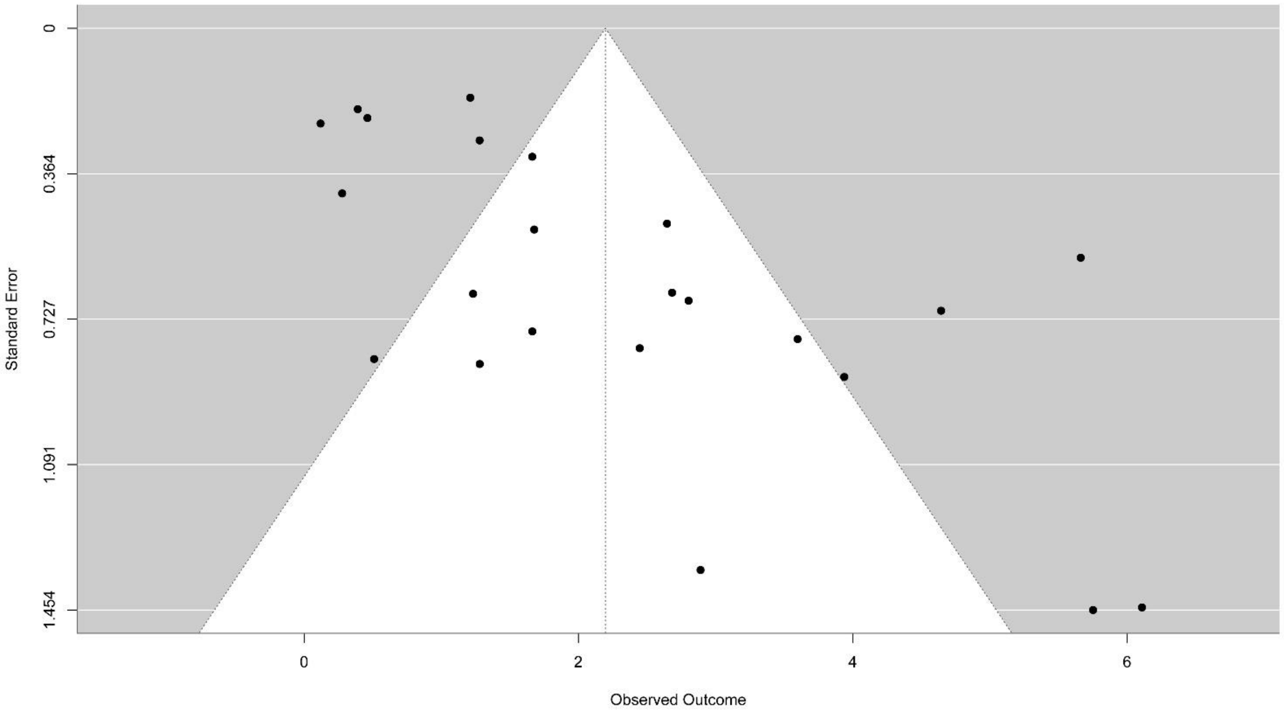


**Figure S1.** Funnel plot of the distribution of studies on MGMT methylation prediction in validation cohorts, with each point corresponding to a study.

**Regression Test for Funnel Plot Asymmetry (Egger's test)**

Model: weighted regression with multiplicative dispersion
Predictor: standard error

Test for Funnel Plot Asymmetry: t = 4.1223, df = 21, p = 0.0005
Limit Estimate (as sei -> 0): b = 0.0138 (CI: -0.6922, 0.7198)

### 6.1.1. MGMT mutation Prediction in Validation Cohorts after applying Trim and Fill method

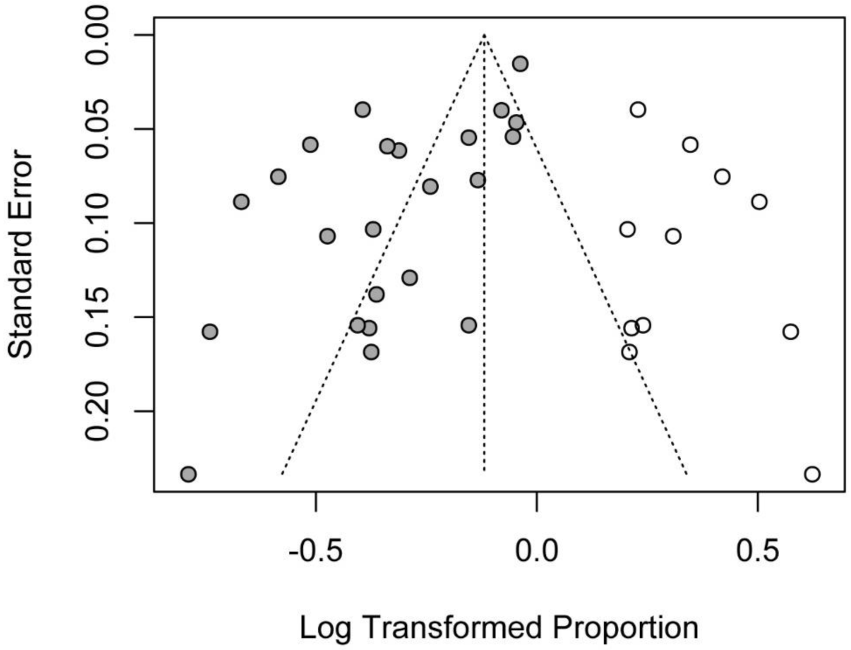


**Figure S2.** Funnel plot showing the distribution of studies on the sensitivity of MGMT methylation status prediction in validation cohort after applying the Trim and Fill method, with adjustments accounting for 11 imputed studies

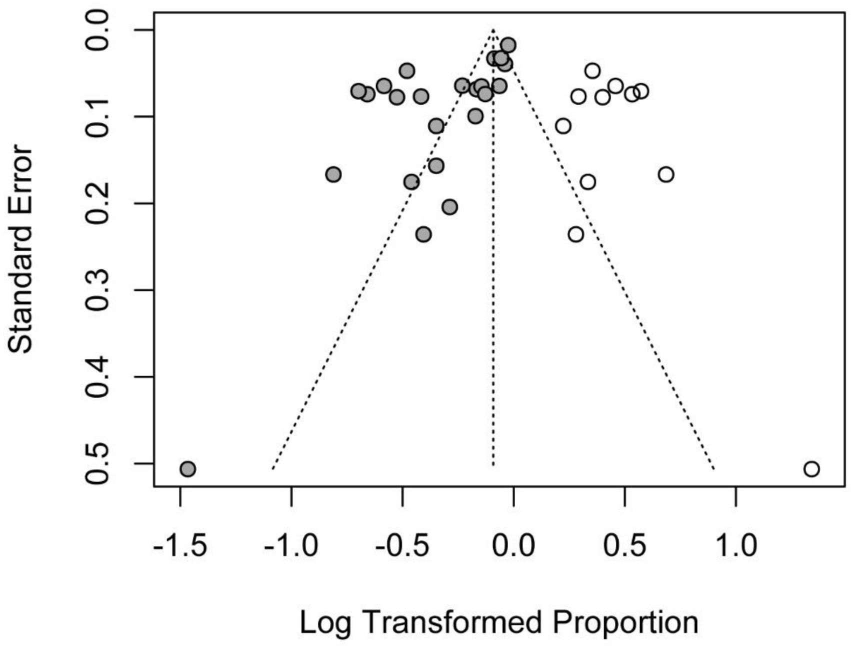


**Figure S3.** Funnel plot showing the distribution of studies on the specificity of MGMT methylation status prediction in validation cohort after applying the Trim and Fill method, with adjustments accounting for 11 imputed studies

## 6.2. MGMT mutation Prediction in Training Cohorts

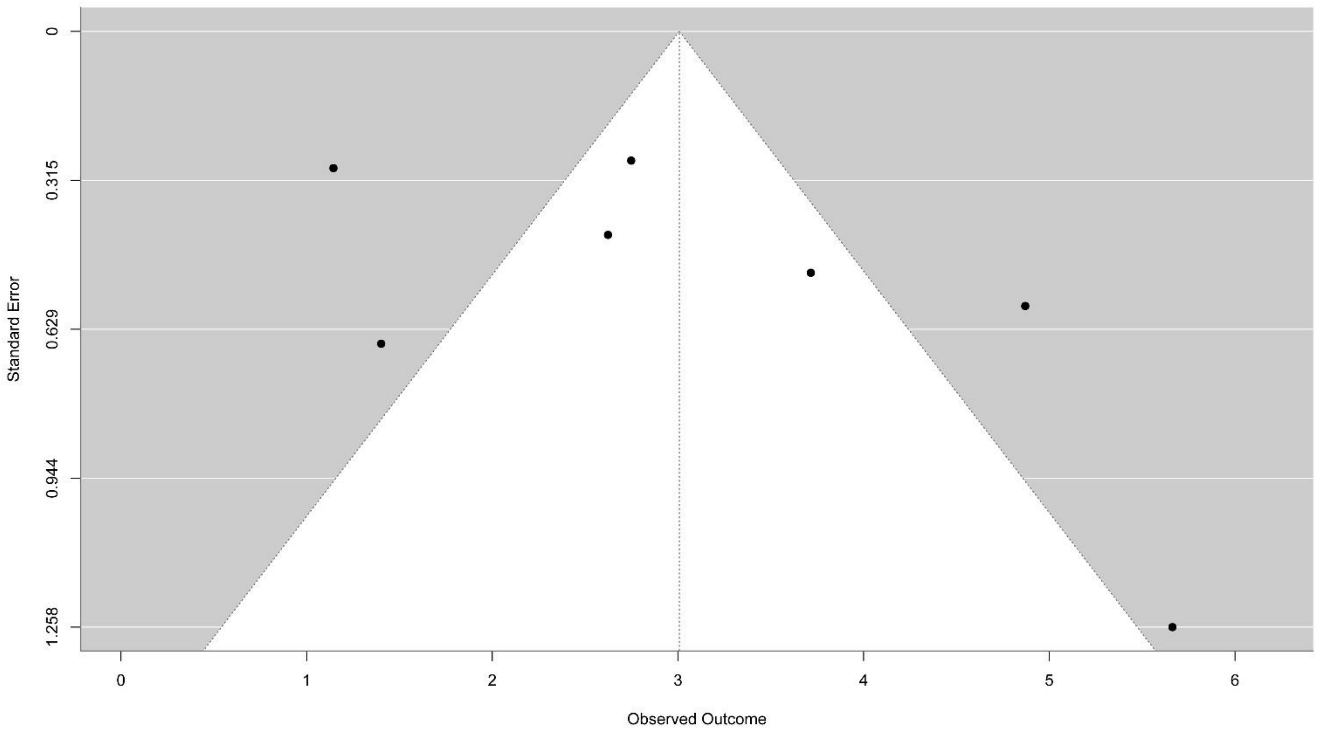


**Figure S4.** Funnel plot of the distribution of studies on MGMT methylation prediction in training cohorts, with each point corresponding to a study.

**Regression Test for Funnel Plot Asymmetry (Egger's test)**

Model: weighted regression with multiplicative dispersion
Predictor: standard error

Test for Funnel Plot Asymmetry: t = 1.4413, df = 5, p = 0.2091
Limit Estimate (as sei -> 0): b = 1.0352 (CI: -1.7644, 3.8348)

## 6.3. ATRX Prediction in Validation Cohorts

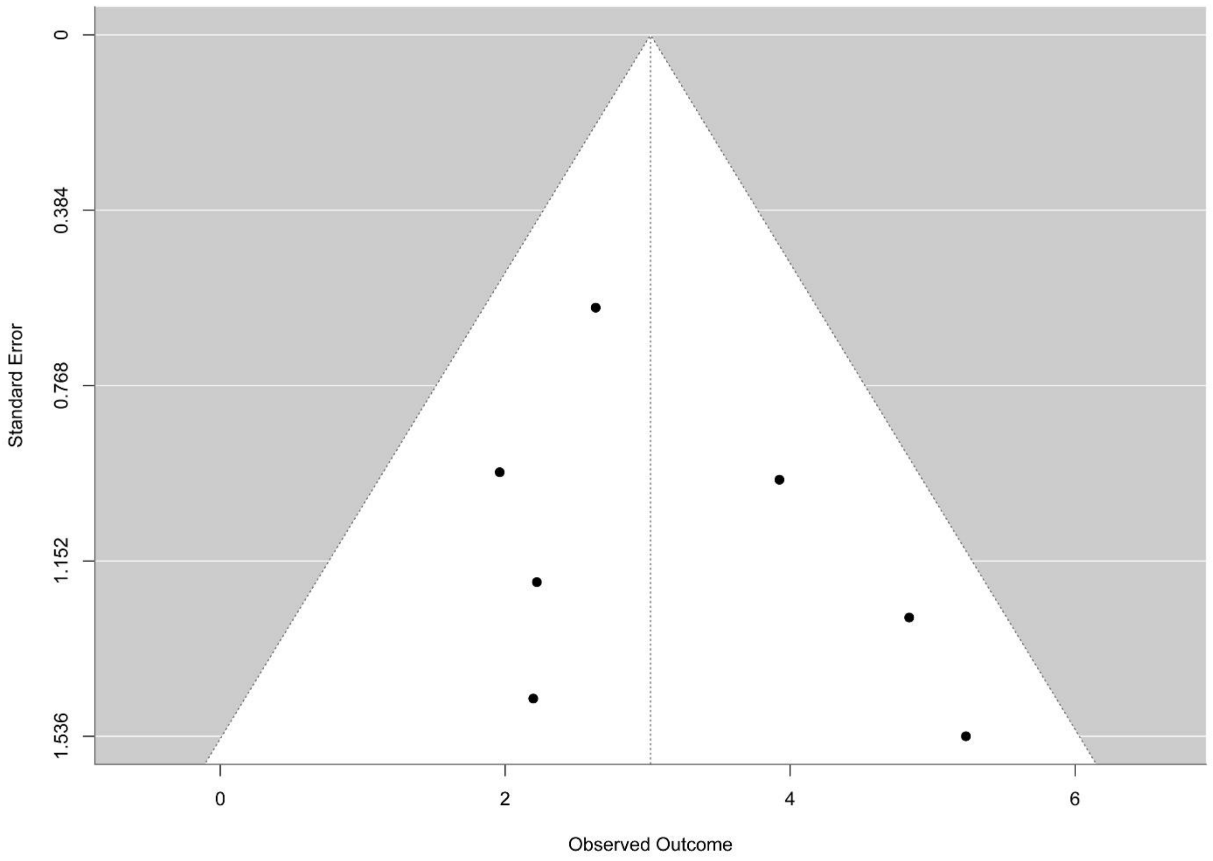


**Figure S5.** Funnel plot of the distribution of studies on ATRX mutation status prediction in validation cohorts, with each point corresponding to a study.

**Regression Test for Funnel Plot Asymmetry (Egger's test)**

Model: weighted regression with multiplicative dispersion
Predictor: standard error

Test for Funnel Plot Asymmetry: t = 1.0393, df = 5, p = 0.3463
Limit Estimate (as sei -> 0): b = 1.7354 (CI: -1.5583, 5.0291)

## 6.4. TERT Prediction in Validation Cohorts

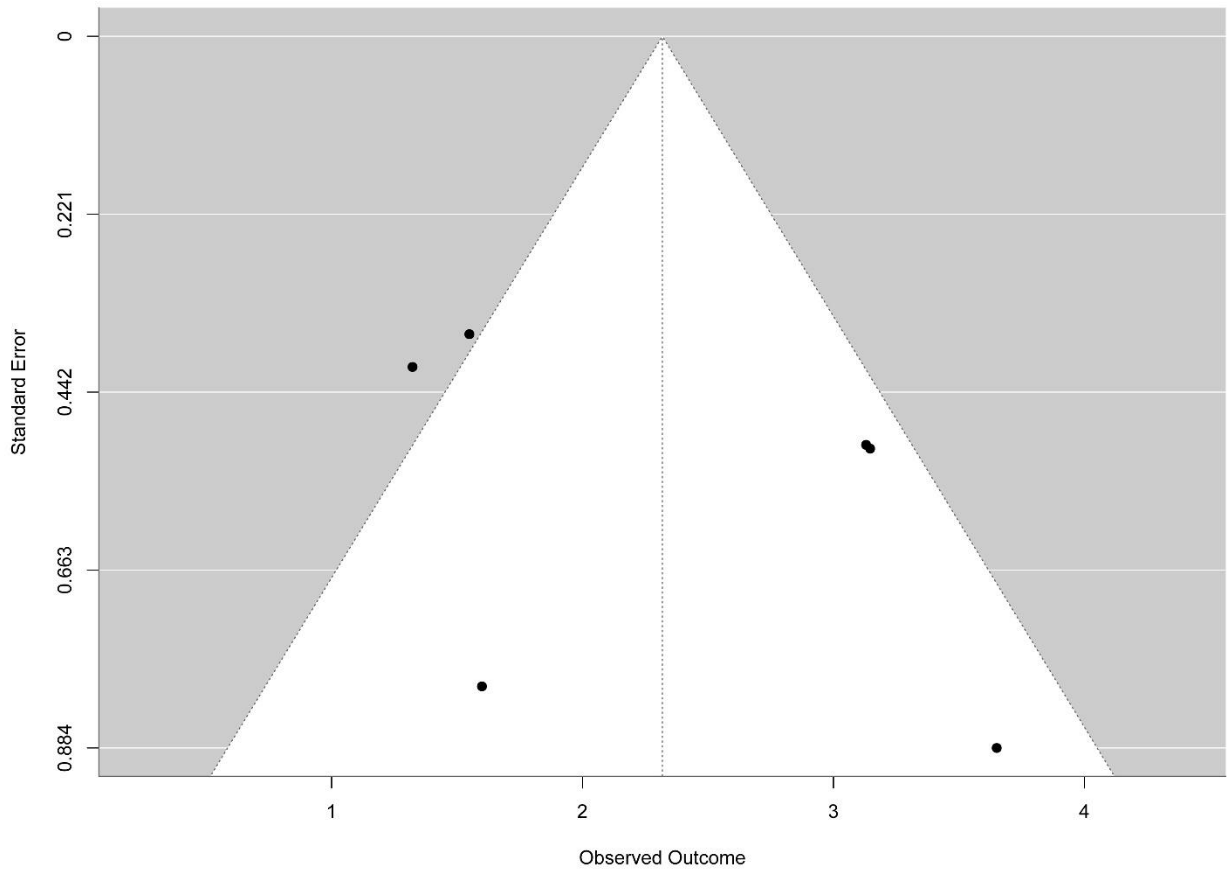


**Figure S6.** Funnel plot of the distribution of studies on TERT mutation prediction in training cohorts, with each point corresponding to a study.

**Regression Test for Funnel Plot Asymmetry (Egger's test)**

Model: weighted regression with multiplicative dispersion
Predictor: standard error

Test for Funnel Plot Asymmetry: t = 1.2333, df = 4, p = 0.2850
Limit Estimate (as sei -> 0): b = 0.6229 (CI: -2.9059, 4.1518)

# 7. Forest plot

## 7.1. MGM Prediction in Training Cohorts: Pooled sensitivity estimate

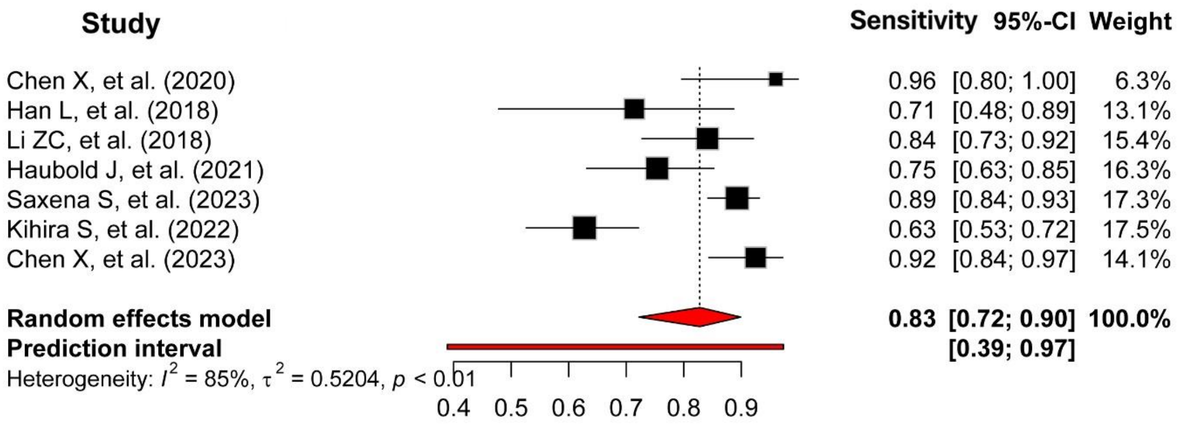


**Figure S7.** Forest plot visualization of training cohorts for sensitivity of MGMT methylation status prediction. The pooled estimate and prediction intervals under a random effects model are depicted at the bottom of the plot. Abbreviations, CI for confidence interval.

## 7.2. MGM Prediction in Training Cohorts: Pooled specificity estimate

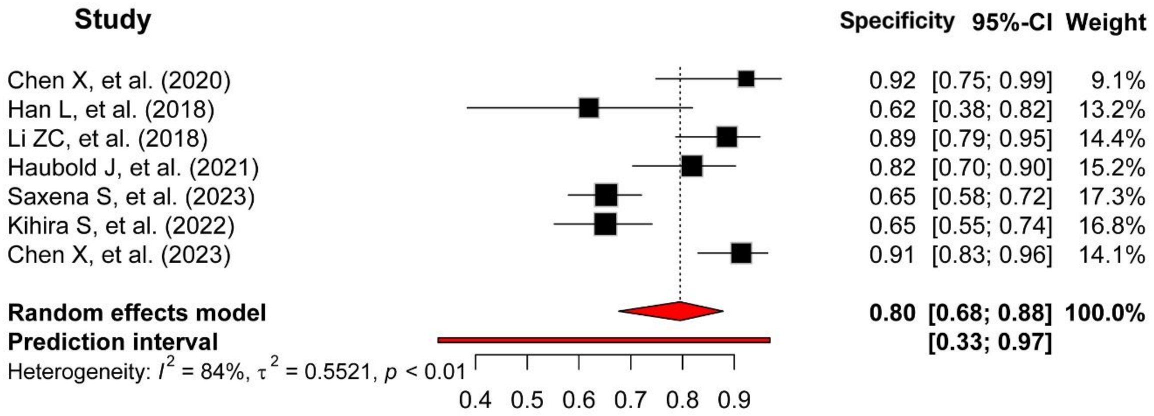


**Figure S8.** Forest plot visualization of training cohorts for specificity of MGMT methylation status prediction. The pooled estimate and prediction intervals under a random effects model are depicted at the bottom of the plot. Abbreviations: CI for confidence interval.

# 8. Cross-Hair Plot

## 8.1. MGMT Prediction in Validation Cohorts

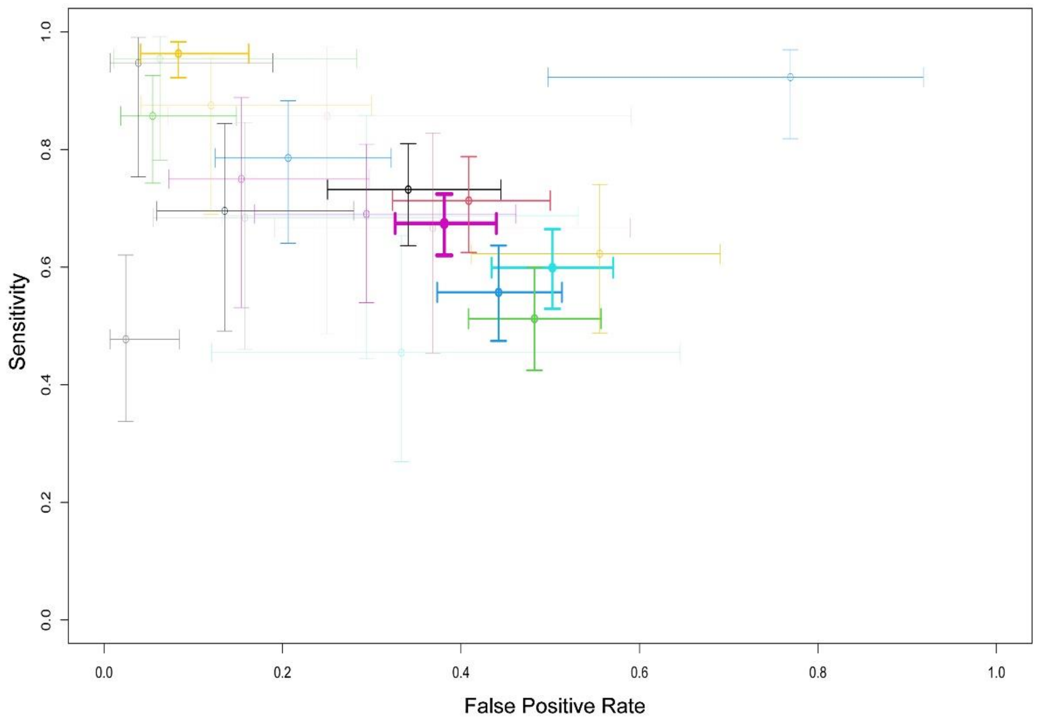


**Figure S9.** Cross-Hair Plot of MGMT Methylation Status Prediction in validation cohorts: The plot displays individual studies in ROC space, with cross-hairs representing the confidence intervals for sensitivity and specificity.

## 8.2. MGMT Prediction in Training Cohorts

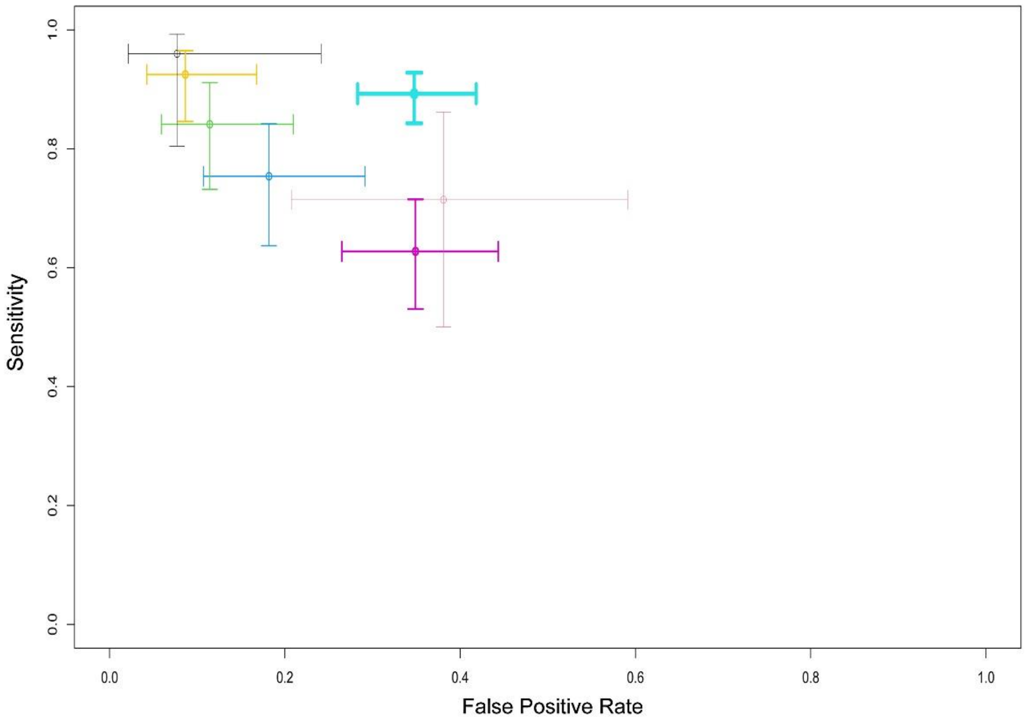


**Figure S10.** Cross-Hair Plot of MGMT Methylation Status Prediction in training cohorts: The plot displays individual studies in ROC space, with cross-hairs representing the confidence intervals for sensitivity and specificity.

## 8.3. ATRX Prediction in Validation Cohorts

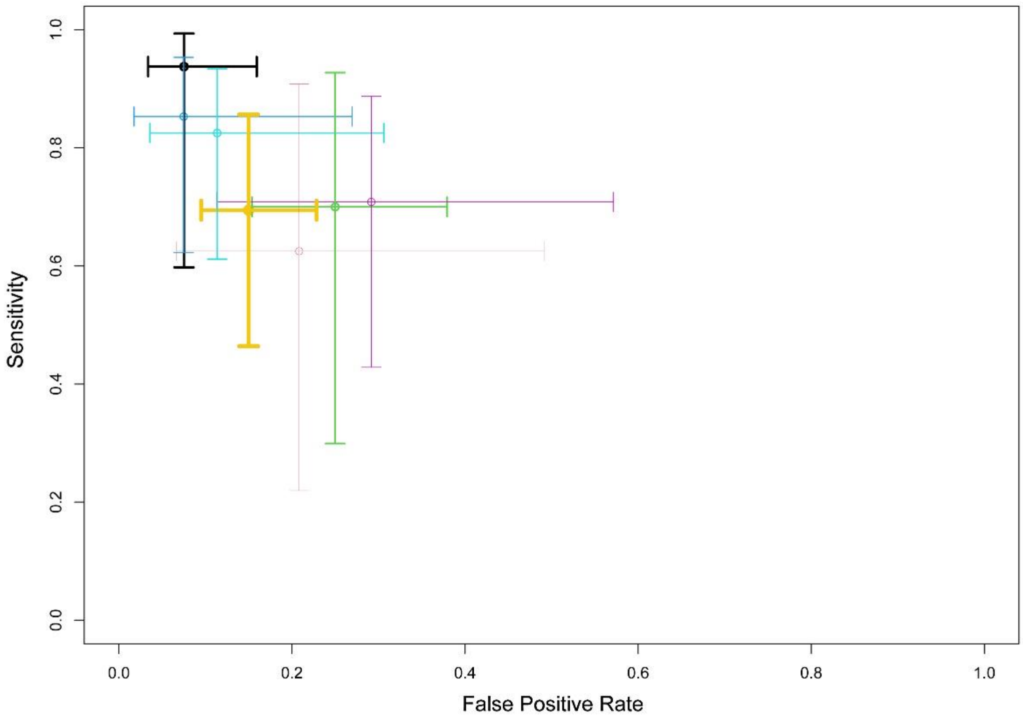


**Figure S11.** Cross-Hair Plot of ATRX Mutation Prediction in Validation cohorts: The plot displays individual studies in ROC space, with cross-hairs representing the confidence intervals for sensitivity and specificity.

## 8.4. TERT Prediction in Validation Cohorts

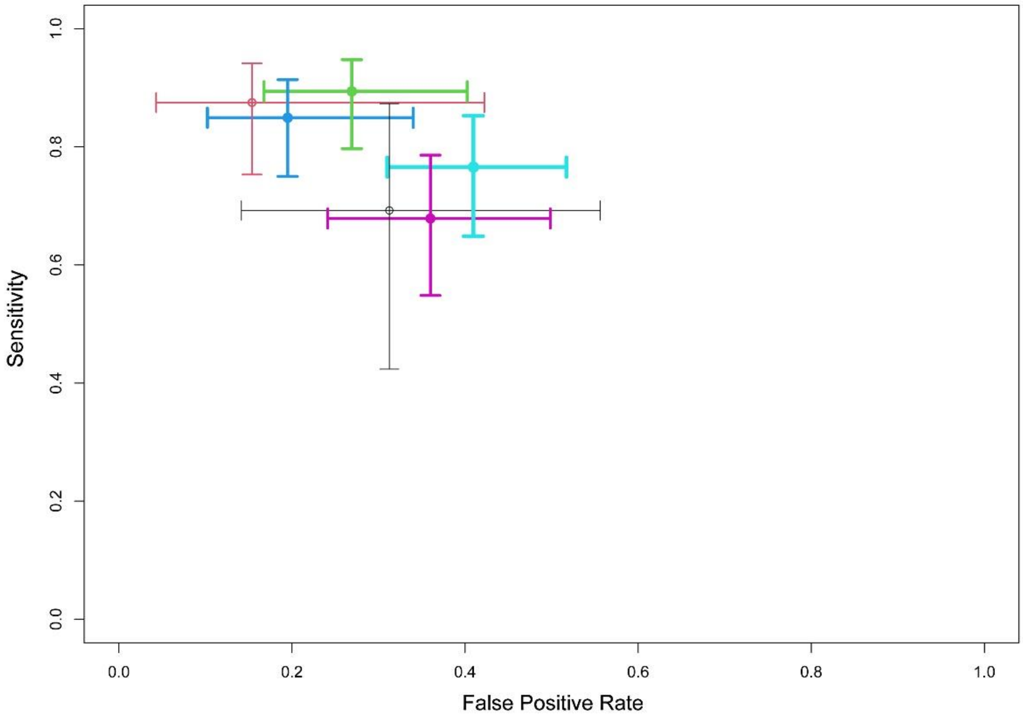


**Figure S12.** Cross-Hair Plot of TERT Mutation Prediction in Validation cohorts: The plot displays individual studies in ROC space, with cross-hairs representing the confidence intervals for sensitivity and specificity.

# 9. Statistical Power

## 9.1. MGMT Prediction in Validation Cohorts

### 9.1.1. Pooled sensitivity estimate

The power analysis of studies included in the meta-analysis on MGMT methylation status prediction reveals variability in their ability to detect changes in sensitivity. Studies like Chen X, et al. (2020) and Haubold J, et al. (2021) show low power (below 80%) for detecting small changes in sensitivity (e.g., 0.1). Conversely, most studies exhibit high power (close to or above 80%) for detecting larger changes in sensitivity (e.g., 0.2), suggesting robust detection capabilities for substantial clinical improvements. Despite significant heterogeneity across the studies ($I^2$ = 80.90%, PI = [0.35; 0.94], p-value < 0.05), the statistical power of observed effect size suggests that the studies had sufficient power to detect the pooled sensitivity of 0.74.

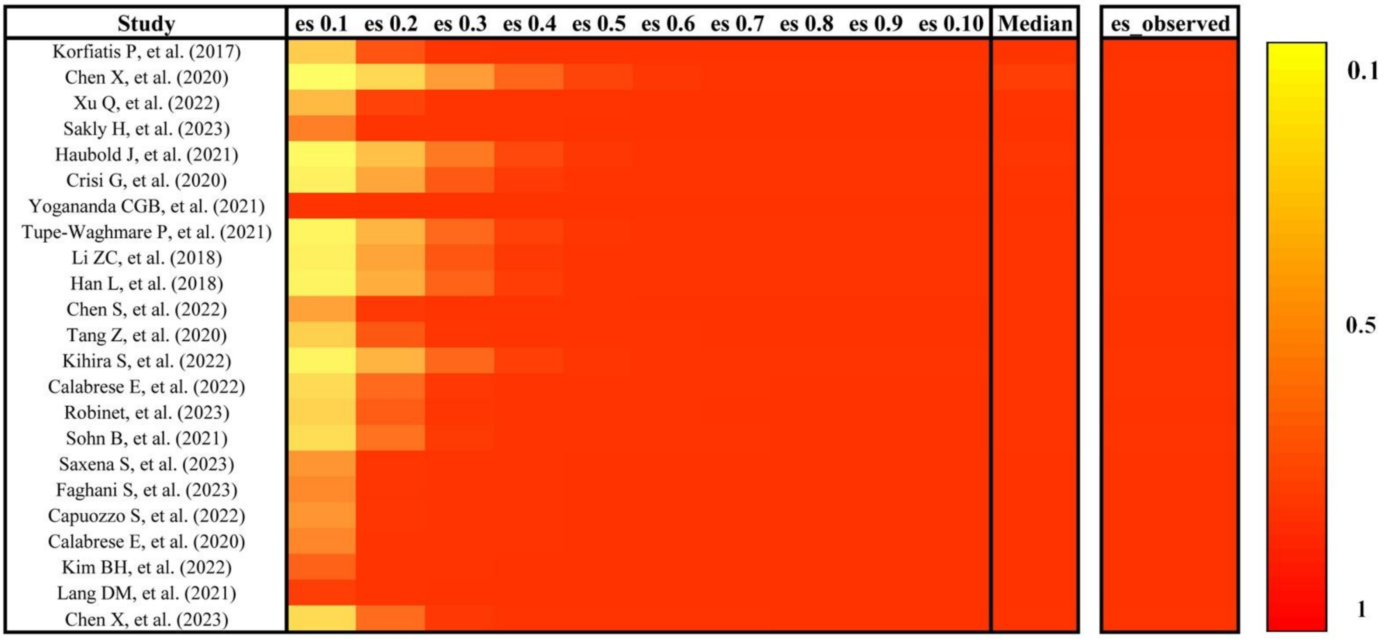


**Figure S13.** Statistical power of studies on MGMT methylation prediction across different hypothetical effect sizes (es) in validation group. Each column (es 0.1, es 0.2, etc.) represents the power to detect effect sizes (sensitivities) ranging from 0.1 to 1.0. The es_observed column shows the power for the observed effect sizes, highlighting each study's ability to reliably detect the reported effects.

### 9.1.2. Pooled specificity estimate

The power analysis of studies on MGMT methylation status prediction in validation cohorts, using specificity as the metric, indicates substantial variability in their ability to detect changes. Studies like Korfiatis P, et al. (2017) and Xu Q, et al. (2022) exhibit high power (close to or above 80%) across a range of effect sizes, indicating robust detection capabilities for clinical meaningful changes in specificity. However, studies such as Chen X, et al. (2020) and Sakly H, et al. (2023) show low power for detecting smaller effect sizes (below 80% for effect sizes of 0.2 and smaller). Despite significant heterogeneity across the studies ($I^2$ = 84.5%, PI = [0.27; 0.96], p-value < 0.05), the statistical power of observed effect size shows that the studies were sufficiently powered to detect the pooled specificity of 0.75.

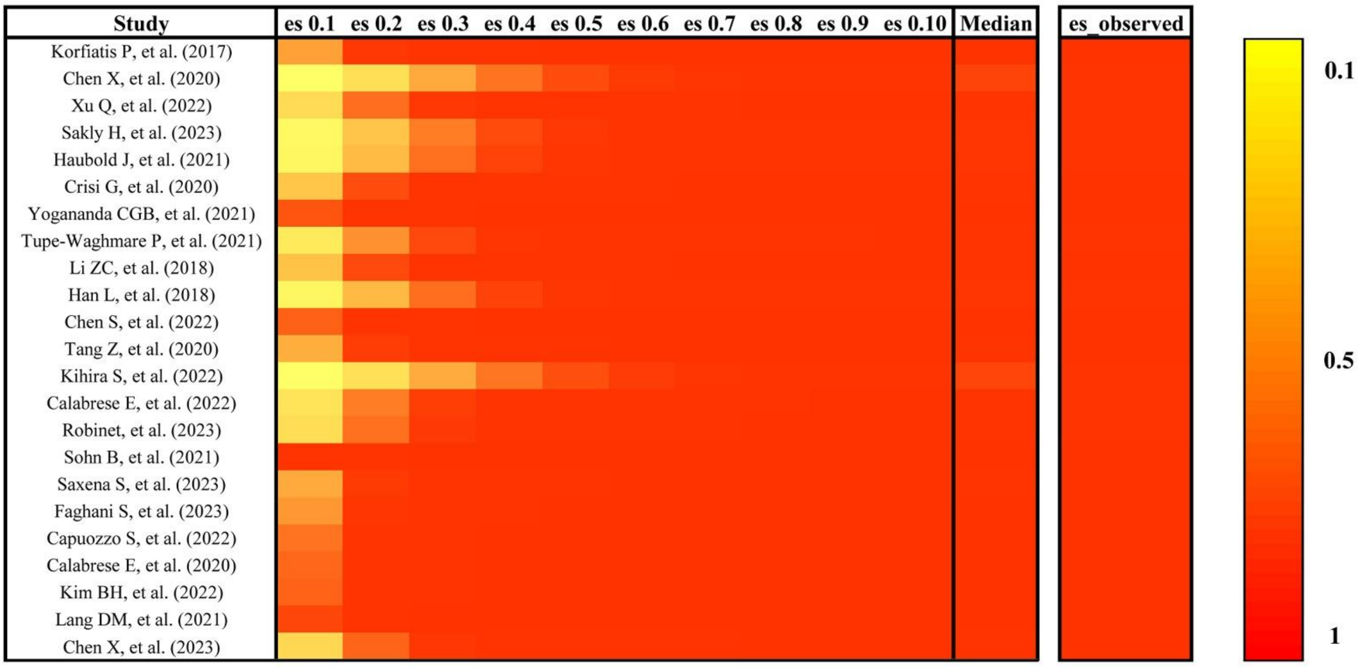


**Figure S14.** Statistical power of studies on MGMTmethylation prediction across different hypothetical effect sizes (es) in validation group. Each column (es 0.1, es 0.2, etc.) represents the power to detect effect sizes (specificities) ranging from 0.1 to 1.0. The es_observed column shows the power for the observed effect sizes, highlighting each study's ability to reliably detect the reported effects.

## 9.2. MGMT Prediction in Training Cohorts

### 9.2.1. Pooled sensitivity estimate

The power analysis of studies on MGMT methylation status prediction in training cohorts, using sensitivity as the evaluation metric, demonstrates high variability in their ability to detect meaningful changes. Studies like Chen X, et al. (2020) and Li ZC, et al. (2018) show high power (close to or above 80%) for detecting a range of effect sizes, indicating robust detection capabilities. Conversely, Han L, et al. (2018) displays lower power (below 80%) for smaller effect sizes (e.g., 0.2). Although there is significant heterogeneity across the studies ($I^2$ = 85.30%, PI = [0.39; 0.97], p-value < 0.05), the statistical power of observed effect size indicates that the studies had sufficient power to detect the pooled sensitivity of 0.83.

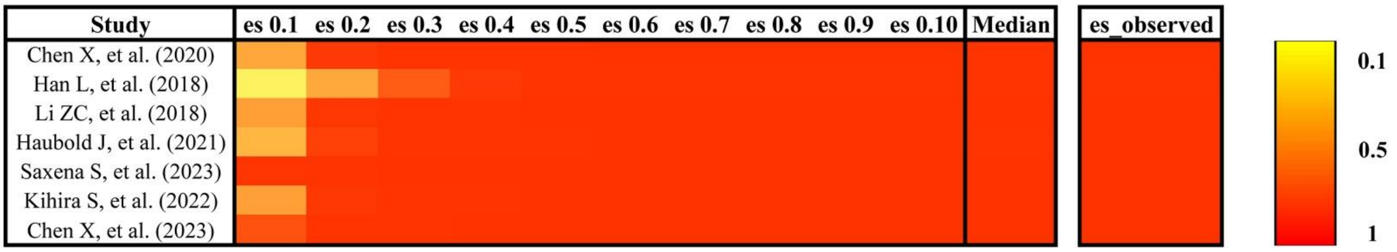


**Figure S15.** Statistical power of studies on MGMTmethylation prediction across different hypothetical effect sizes (es) in training group. Each column (es 0.1, es 0.2, etc.) represents the power to detect effect sizes (sensitivities) ranging from 0.1 to 1.0. The es_observed column shows the power for the observed effect sizes, highlighting each study's ability to reliably detect the reported effects.

### 9.2.2. Pooled specificity estimate

The power analysis of studies on MGMT methylation status prediction in training cohorts, using specificity as the evaluation metric, reveals significant variability in their ability to detect changes. Studies like Chen X, et al. (2020) and Li ZC, et al. (2018) show high power (close to or above 80%) across a range of effect sizes, indicating robust detection capabilities. Conversely, Han L, et al. (2018) displays lower power (below 80%) for smaller effect sizes (e.g., 0.2). Despite significant heterogeneity ($I^2$ = 83.6%, PI = [0.33; 0.97], p-value < 0.05), the statistical power of observed effect size shows that the studies were adequately powered to detect the pooled specificity of 0.79.

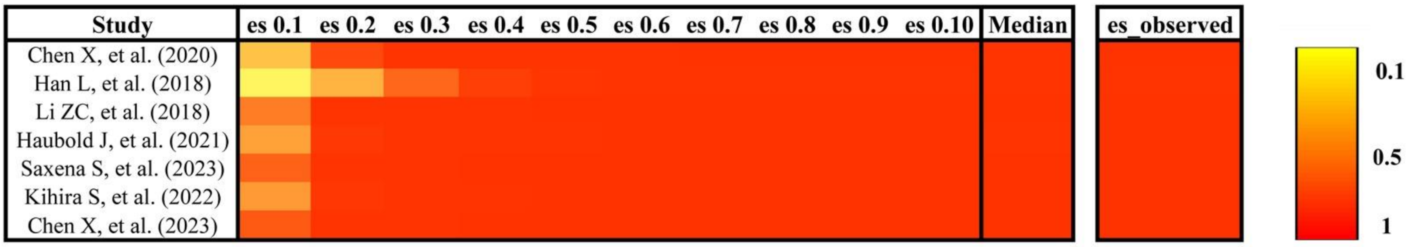


**Figure S16.** Statistical power of studies on MGMT methylation prediction across different hypothetical effect sizes (es) in training group. Each column (es 0.1, es 0.2, etc.) represents the power to detect effect sizes (specificities) ranging from 0.1 to 1.0. The es_observed column shows the power for the observed effect sizes, highlighting each study's ability to reliably detect the reported effects.

### 9.3. ATRX Prediction in Validation Cohorts

### 9.3.1. Pooled sensitivity estimate

The power analysis of studies on ATRX mutation status prediction in validation cohorts, using sensitivity as the evaluation metric, shows notable variability in their ability to detect meaningful changes. Studies like Calabrese E, et al. (2022) and Liu Z, et al. (2024) exhibit high power (close to or above 80%) across a range of effect sizes, indicating strong detection capabilities. Conversely, Haubold J, et al. (2021) demonstrates lower power for smaller effect sizes (e.g., 0.2). The validation dataset shows no significant heterogeneity (p-value = 0.79), and the statistical power of observed effect size confirms that the studies were well-powered to detect the pooled sensitivity of 0.79.

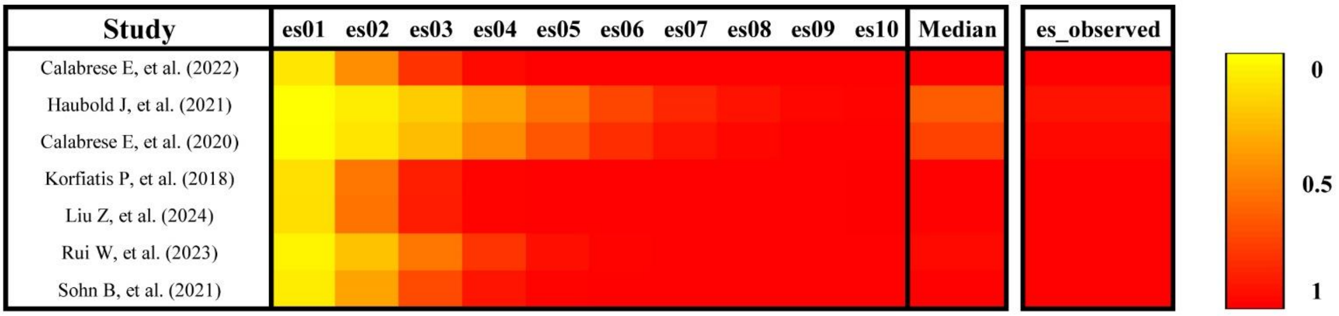


**Figure S17.** Statistical power of studies on ATRX mutation prediction across different hypothetical effect sizes (es) in validation group. Each column (es 0.1, es 0.2, etc.) represents the power to detect effect sizes (sensitivities) ranging from 0.1 to 1.0. The es_observed column shows the power for the observed effect sizes, highlighting each study's ability to reliably detect the reported effects.

### 9.3.2. Pooled specificity estimate

The power analysis of studies on ATRX mutation status prediction in validation cohorts, using specificity as the evaluation metric, reveals notable variability in their ability to detect meaningful changes. While most studies show robust power for clinically meaningful effect sizes, studies like Haubold J, et al. (2021) demonstrates lower power for smaller effect sizes (e.g., 0.2). Despite moderate heterogeneity ($I^2$ = 40.7%, PI = [0.62; 0.95], p-value = 0.12), the statistical power of observed effect size suggests that the studies had adequate power to detect the pooled specificity of 0.85.

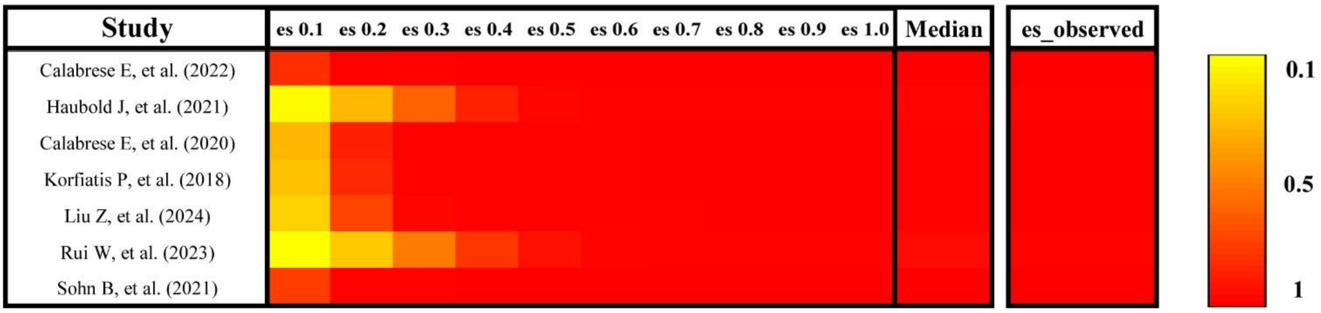


**Figure S18.** Statistical power of studies on ATRX prediction across different hypothetical effect sizes (es) in validation group. Each column (es 0.1, es 0.2, etc.) represents the power to detect effect sizes (specificities) ranging from 0.1 to 1.0. The es_observed column shows the power for the observed effect sizes, highlighting each study's ability to reliably detect the reported effects.

## 9.4. TERT Prediction in Validation Cohorts

### 9.4.1. Pooled sensitivity estimate

The power analysis of studies on TERT mutation status prediction in validation cohorts, using sensitivity as the evaluation metric, shows significant variability in their ability to detect meaningful changes. Studies like Calabrese E, et al. (2022) and Zhang H, et al. (2023) exhibit high power (close to or above 80%) across a range of effect sizes, indicating robust detection capabilities. Conversely, Buz-Yalug B, et al. (2024) demonstrates lower power for smaller effect sizes (e.g., 0.2). Despite significant heterogeneity across the studies ($I^2 = 60.2\%$, PI = [0.51; 0.94], p-value = 0.03), the statistical power of observed effect size indicates that the studies had sufficient power to detect the pooled sensitivity of 0.81.

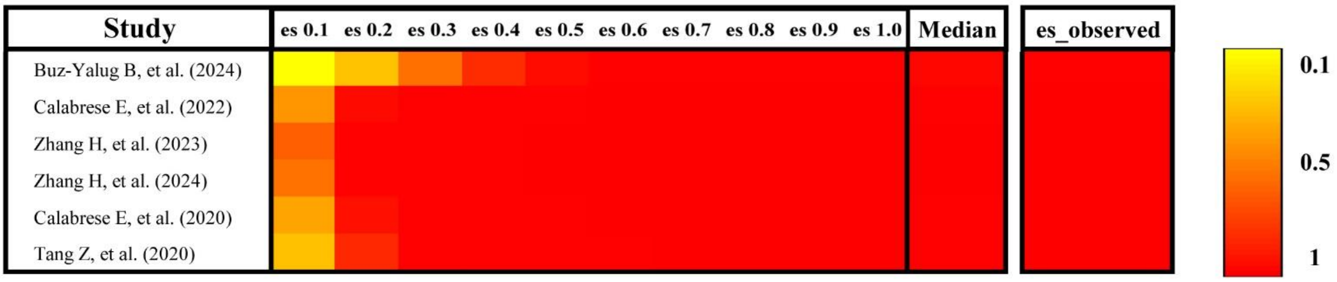


**Figure S19.** Statistical power of studies on TERT mutation prediction across different hypothetical effect sizes (es) in validation group. Each column (es 0.1, es 0.2, etc.) represents the power to detect effect sizes (sensitivities) ranging from 0.1 to 1.0. The es_observed column shows the power for the observed effect sizes, highlighting each study's ability to reliably detect the reported effects.

### 9.4.2. Pooled specificity estimate

The power analysis of studies on TERT mutation status prediction in validation cohorts, using specificity as the evaluation metric, shows significant variability in their ability to detect meaningful changes. While most studies show strong power for larger effect sizes, studies like Buz-Yalug B, et al. (2024) and Calabrese E, et al. (2022) demonstrate lower power for smaller effect sizes (e.g., 0.2). Despite moderate heterogeneity ($I^2 = 40.0\%$, PI = [0.46; 0.86], p-value = 0.14), the statistical power of observed effect size shows that the studies were adequately powered to detect the pooled specificity of 0.69.

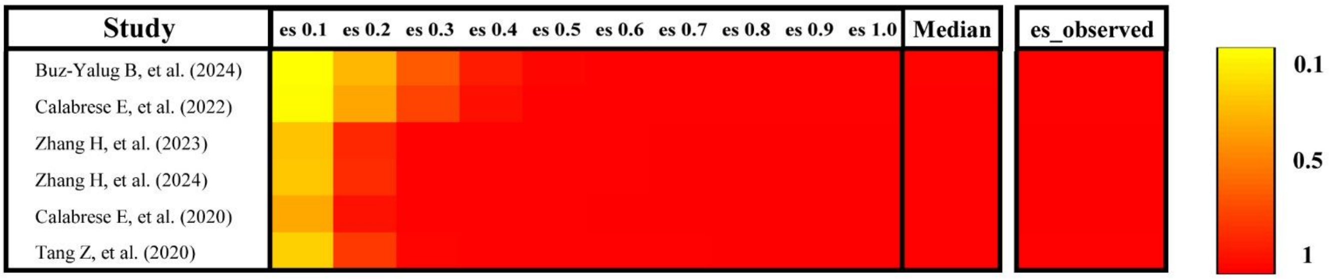


**Figure S20.** Statistical power of studies on TERT mutation prediction across different hypothetical effect sizes (es) in validation group. Each column (es 0.1, es 0.2, etc.) represents the power to detect effect sizes (specificities) ranging from 0.1 to 1.0. The es_observed column shows the power for the observed effect sizes, highlighting each study's ability to reliably detect the reported effects.

# 10. Meta-Regression Analysis of MGMT Prediction in Validation Cohorts

## 10.1. Tumor Grade

### 10.1.1. SROC curves

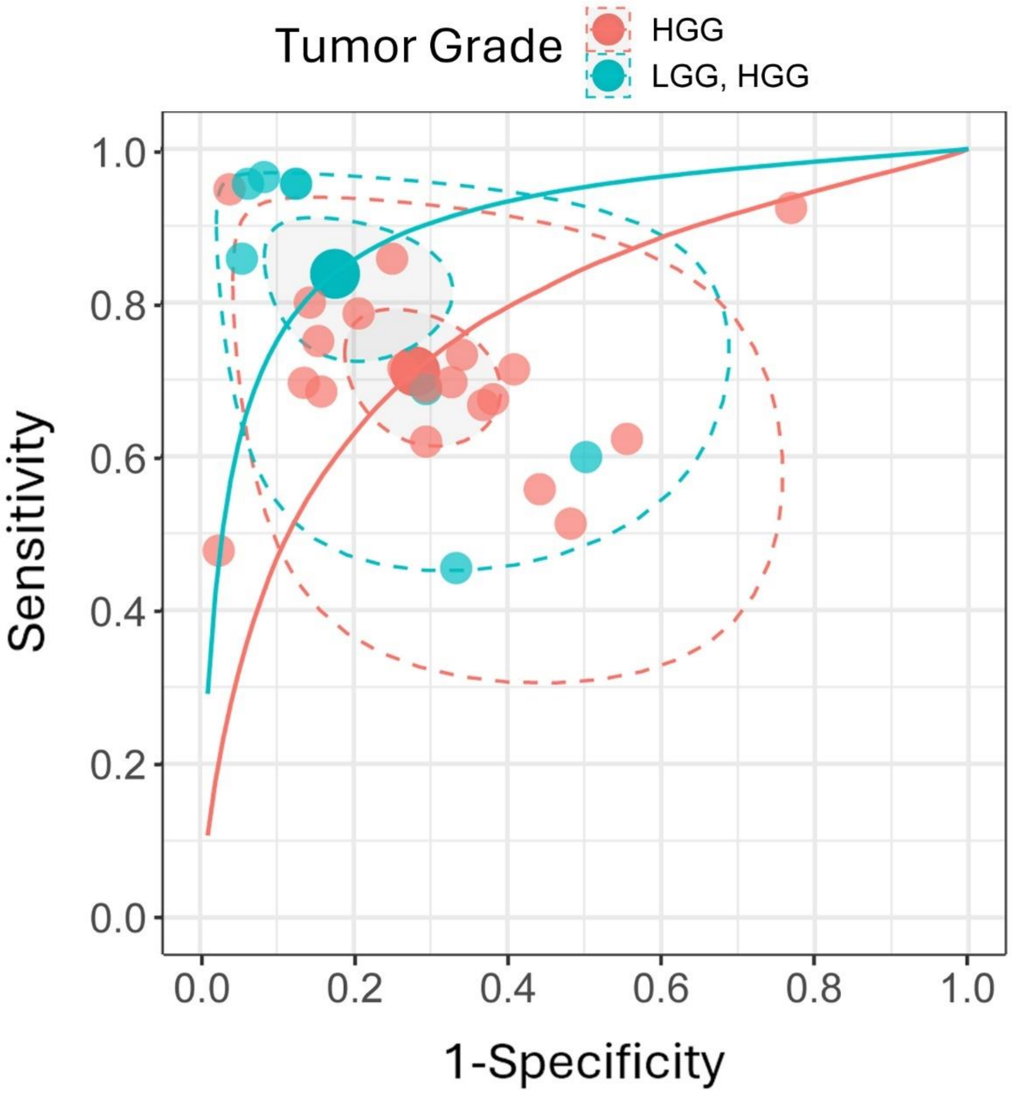


**Figure S21.** MGMT Prediction Comparison in Glioma Grades. The SROC plot shows sensitivity and 1-specificity for high-grade gliomas (HGG, red curve) and combined high and low-grade gliomas (blue curve). Dotted lines indicate the 95% prediction region, and shaded areas represent the 95% credible region from the bivariate model.

### 10.1.2. Accuracy vs. Covariate Plot

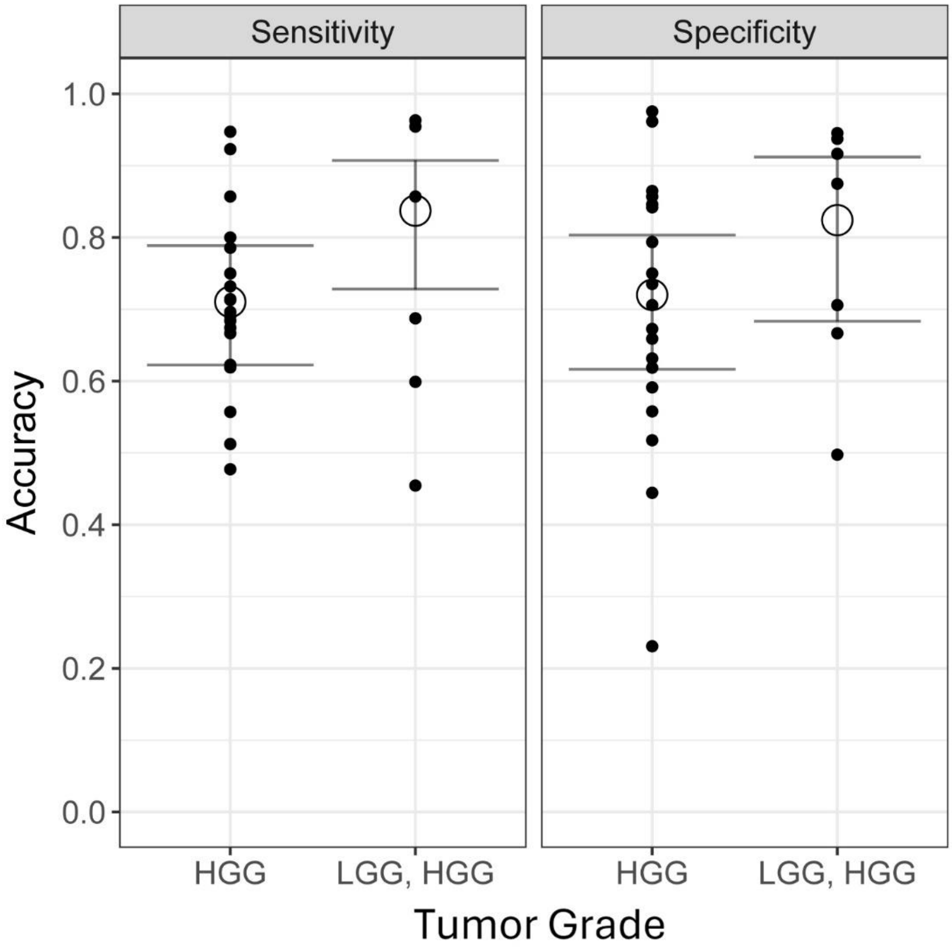


**Figure S22.** Accuracy vs. Glioma Grade Plot for MGMT Prediction. The left panel shows sensitivity, and the right panel shows specificity for different glioma grades (HGG and combined LGG, HGG). Vertical bars represent the 95% credible intervals from the bivariate model, while black points denote study-specific data points for each grade level.

## 10.2. Clinical Information

### 10.2.1. SROC curves

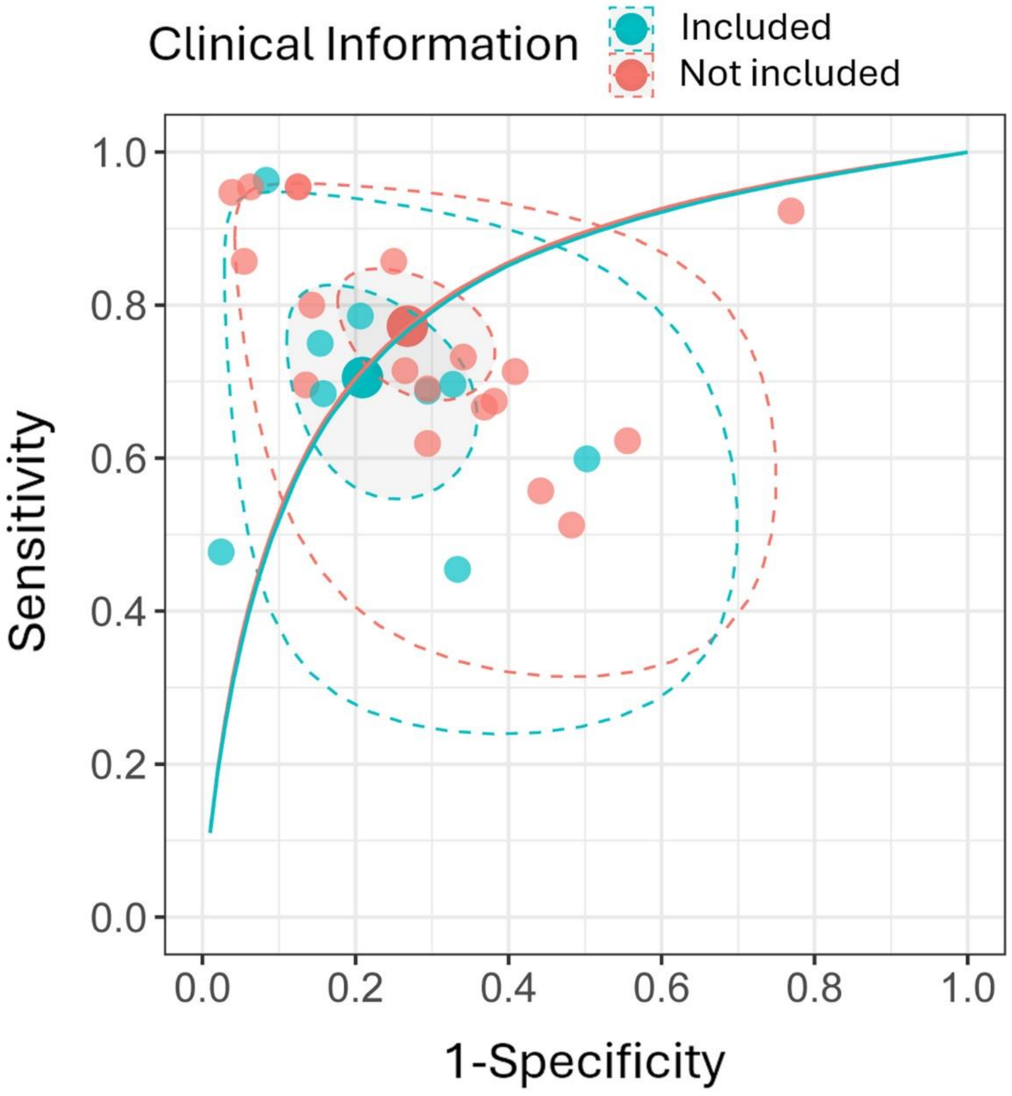


**Figure S23.** MGMT Prediction Comparison in Clinical Information. The SROC plot illustrates sensitivity and 1-specificity for the inclusion (blue curve) and exclusion (red curve) of non-radiomics information such as age and sex. Dotted lines indicate the 95% prediction region, while shaded areas represent the 95% credible region from the bivariate model.

### 10.2.2. Accuracy vs. Covariate Plot

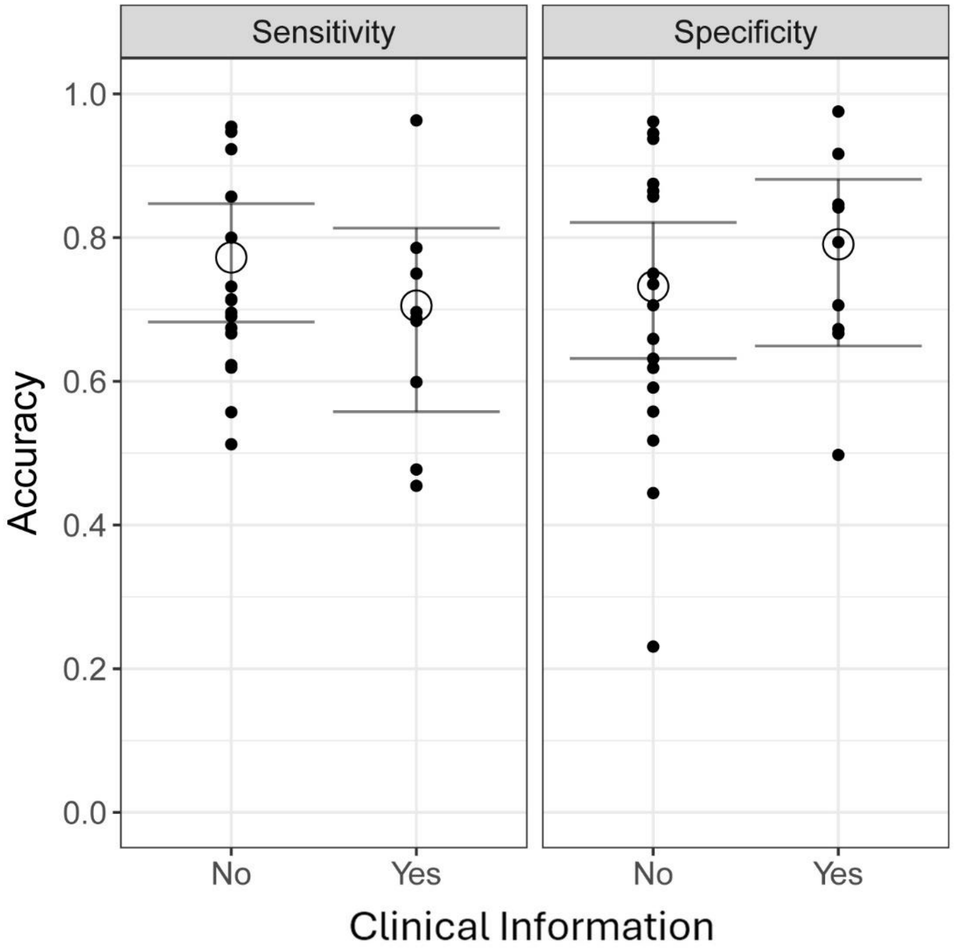


**Figure S24.** Accuracy vs. Clinical Information Plot for MGMT Prediction. The left panel shows sensitivity, and the right panel shows specificity for models with and without the inclusion of non-radiomics information. Vertical bars represent the 95% credible intervals from the bivariate model, while black points denote study-specific data points for each level.

## 10.3. Data Augmentation

### 10.3.1. SROC curves

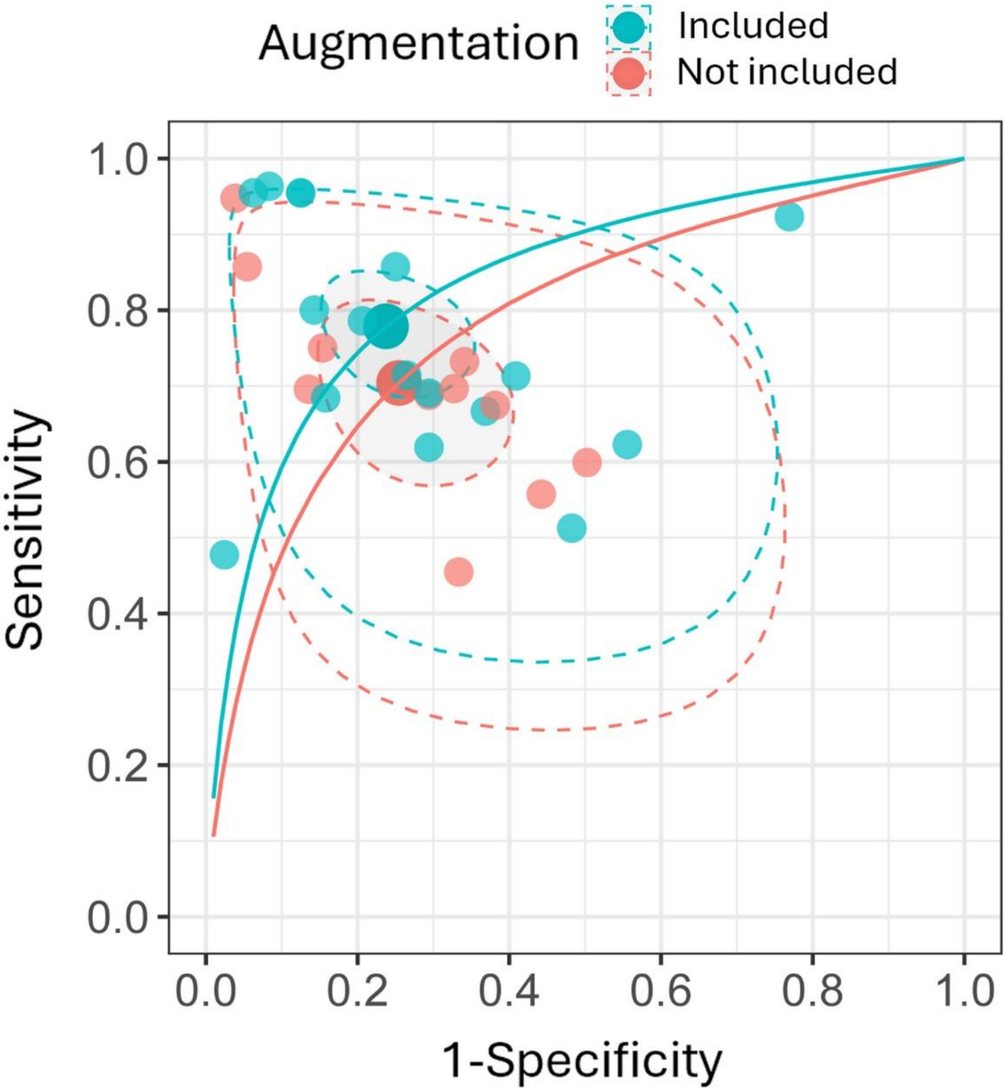


**Figure S25.** MGMT Prediction Comparison in Data Augmentation: The plot illustrates sensitivity and 1-specificity for models with (blue curve) and without (red curve) the use of data augmentation, including classical and deep learning methods. Dotted lines indicate the 95% prediction region, while shaded areas represent the 95% credible region from the bivariate model.

### 10.3.2. Accuracy vs. Covariate Plot

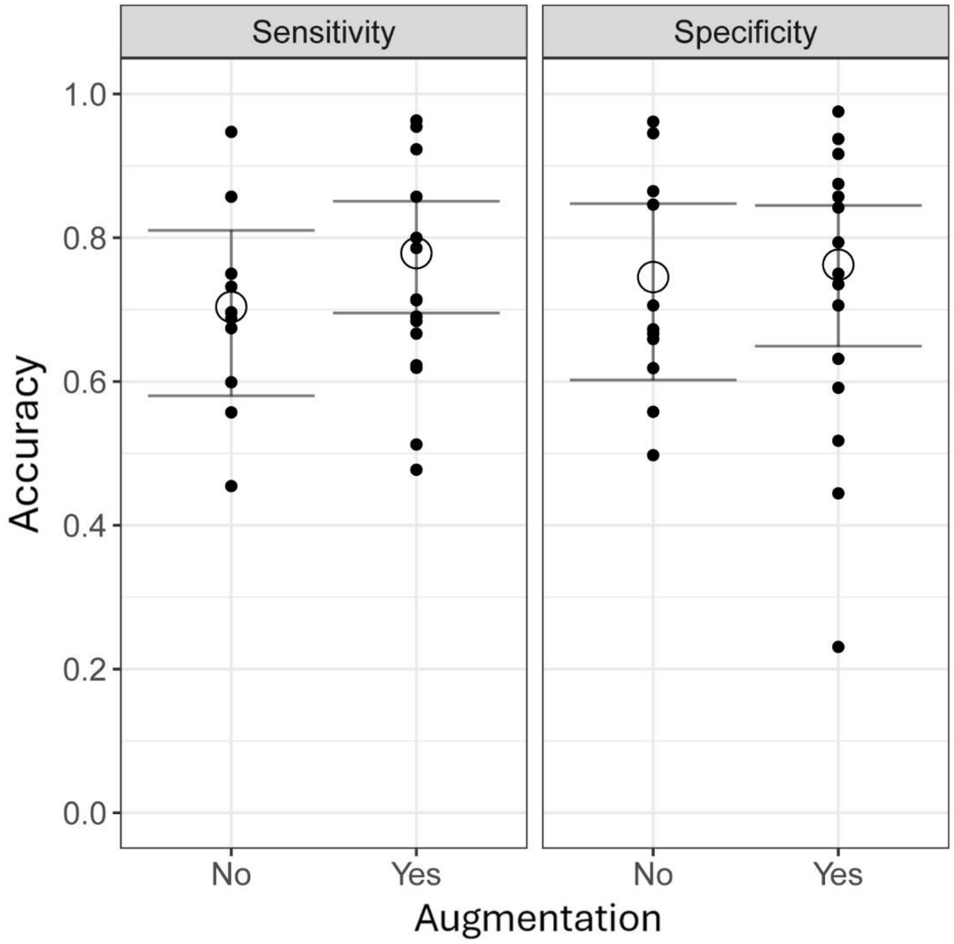


**Figure S26.** Accuracy vs. Data Augmentation Plot for MGMT Prediction. The left panel shows sensitivity, and the right panel shows specificity for models with and without data augmentation. Vertical bars represent the 95% credible intervals from the bivariate model, while black points denote study-specific data points for each model type.

## 10.4. Dataset

### 10.4.1. SROC curves

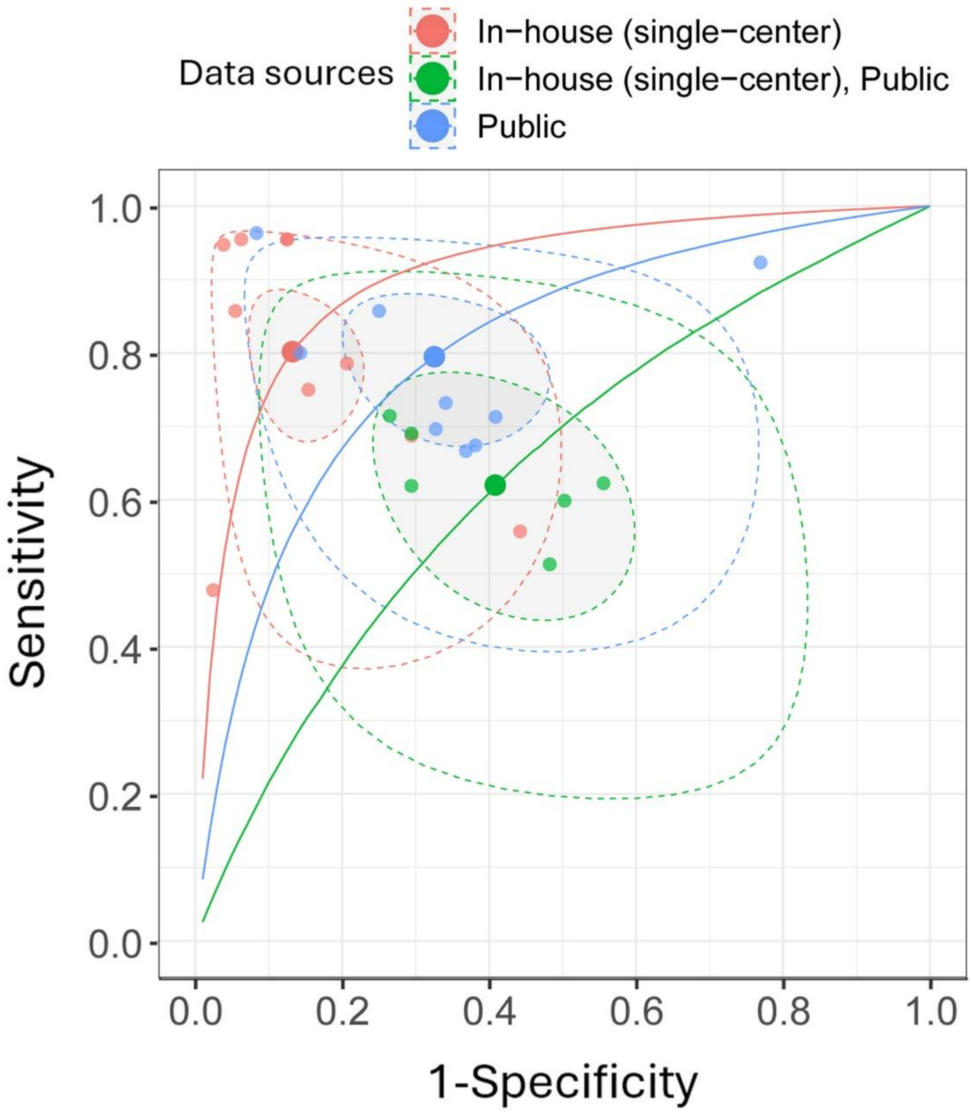


**Figure S27.** MGMT Prediction Comparison in Data Sources. The plot shows sensitivity and 1-specificity for different data sources: In-house (single-center) (red curve), a combination of In-house (single-center) and Public datasets (green curve), and Public data sources (blue curve). Dotted lines indicate the 95% prediction region, while shaded areas represent the 95% credible region from the bivariate model.

### 10.4.2. Accuracy vs. Covariate Plot

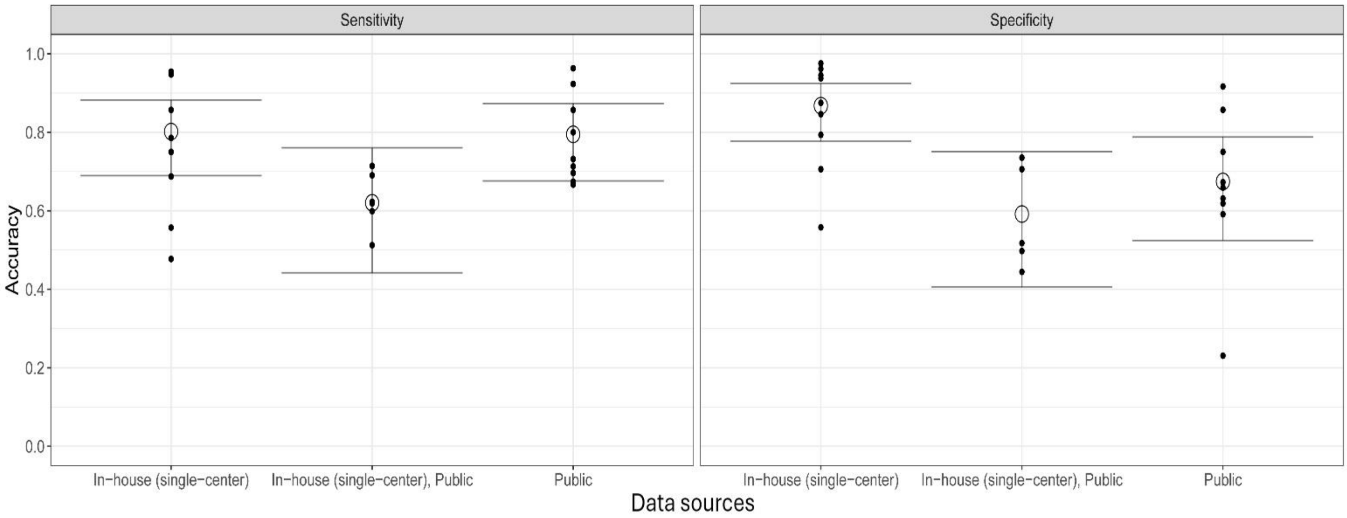


**Figure S28.** Accuracy vs. Data Sources Plot for MGMT Prediction. The left panel shows sensitivity, and the right panel shows specificity for different data sources: In-house (single-center), a combination of In-house (single-center) and Public datasets, and Public data sources. Vertical bars represent the 95% credible intervals from the bivariate model, while black points denote study-specific data points for each data source.

## 10.5. Segmentation Method

### 10.5.1. SROC curves

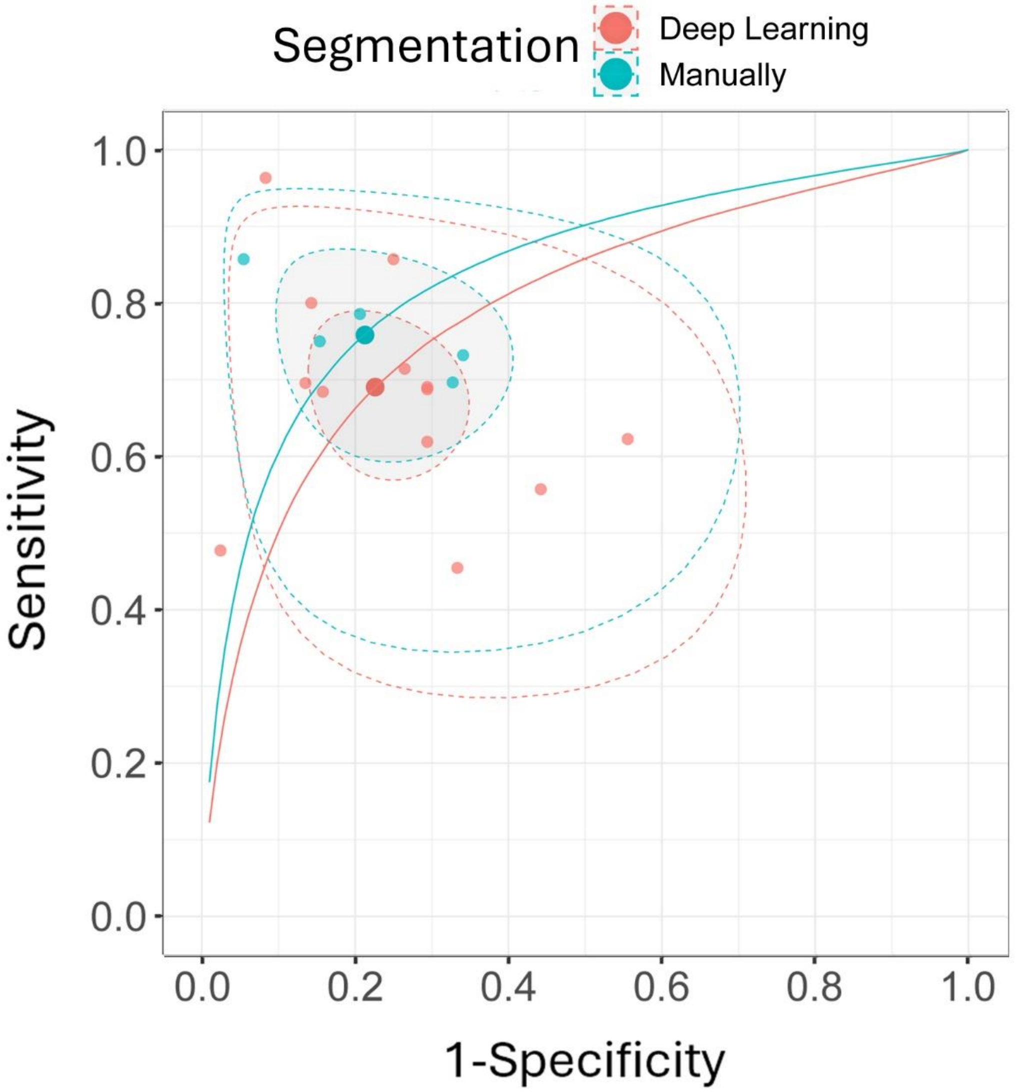


**Figure S29.** MGMT Prediction Comparison in Segmentation Methods. The plot shows sensitivity and 1-specificity for different segmentation methods: deep learning approach (red curve) and manual tumor delineation (blue curve). Dotted lines indicate the 95% prediction region, while shaded areas represent the 95% credible region from the bivariate model.

### 10.5.2. Accuracy vs. Covariate Plot

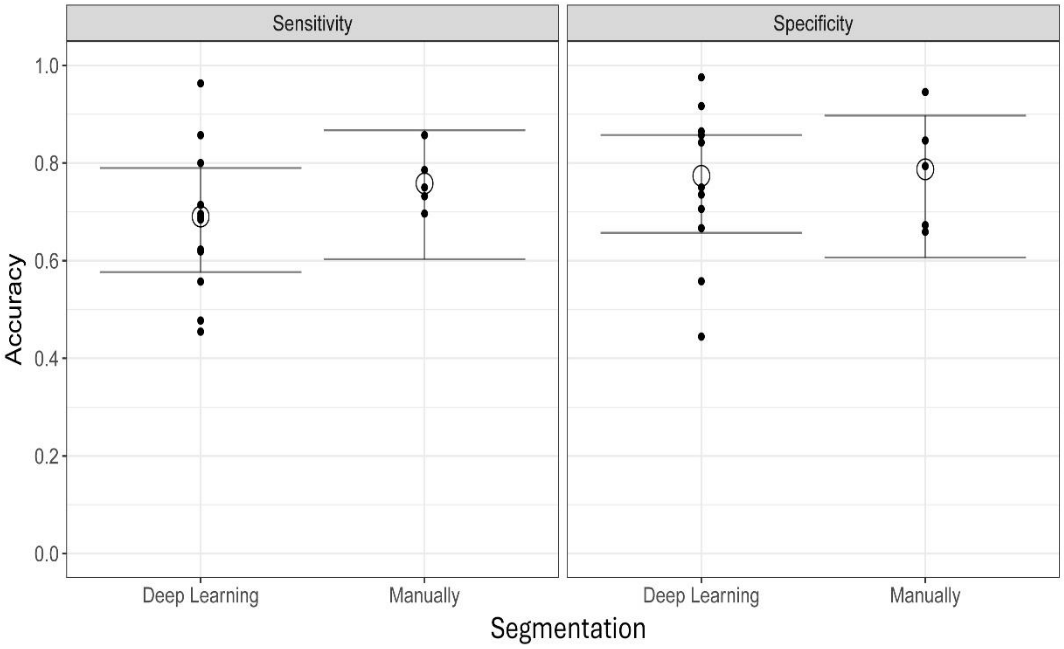


**Figure S30.** Accuracy vs. Segmentation Method Plot for MGMT Prediction. The left panel shows sensitivity, and the right panel shows specificity for different segmentation methods: deep learning and manual tumor delineation. Vertical bars represent the 95% credible intervals from the bivariate model, while black points denote study-specific data points for each method.

## 10.6. Feature Extraction

### 10.6.1. SROC curves

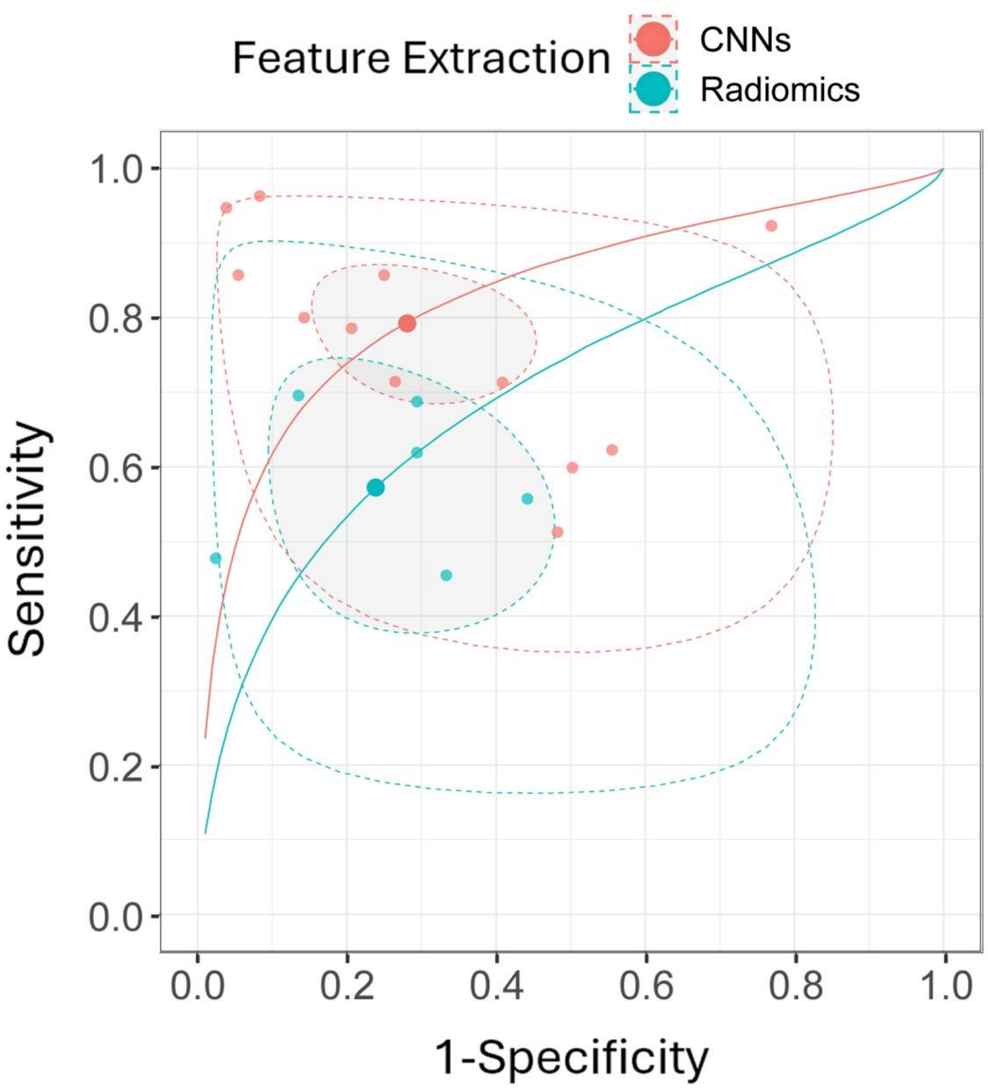


**Figure S31.** MGMT Prediction Comparison in Feature Extraction Methods. The plot shows sensitivity and 1-specificity for different feature extraction methods: CNNs (red curve) and conventional radiomics methods (blue curve). Dotted lines indicate the 95% prediction region, while shaded areas represent the 95% credible region from the bivariate model.

### 10.6.2. Accuracy vs. Covariate Plot

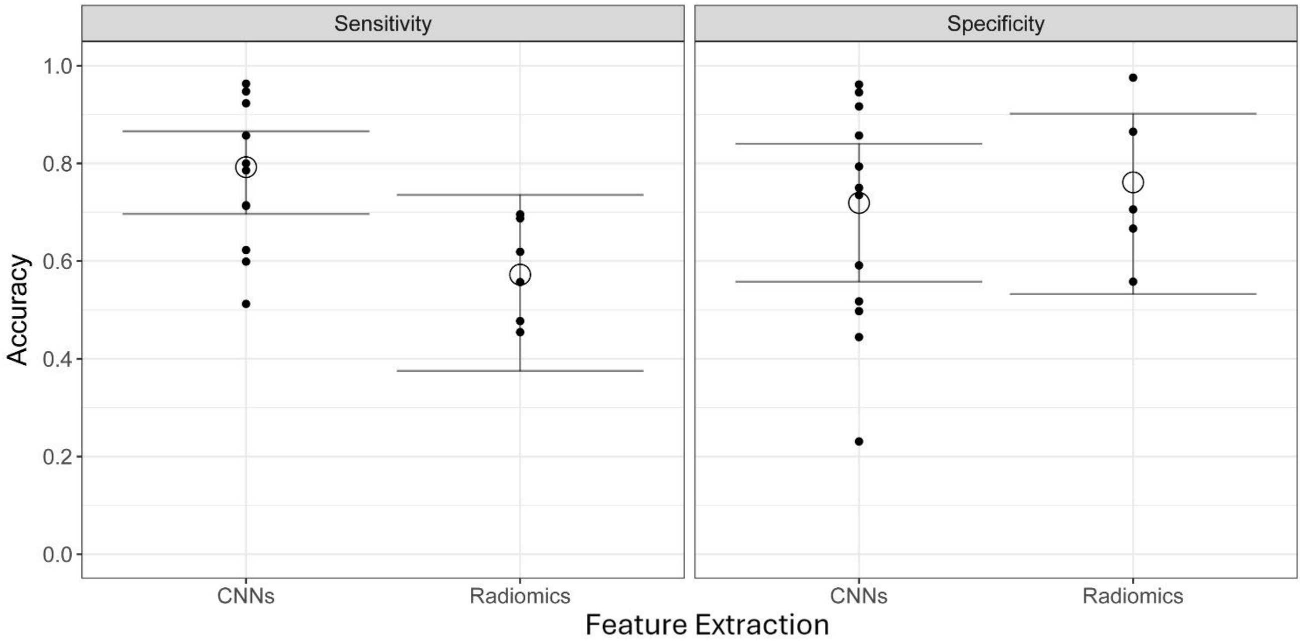


**Figure S32.** Accuracy vs. Feature Extraction Method Plot for MGMT Prediction. The left panel shows sensitivity, and the right panel shows specificity for feature extraction methods (CNNs and conventional radiomics). Vertical bars represent the 95% credible intervals from the bivariate model, while black points denote study-specific data points for each method.

## 10.7. Pretrained Model

### 10.7.1. SROC curves

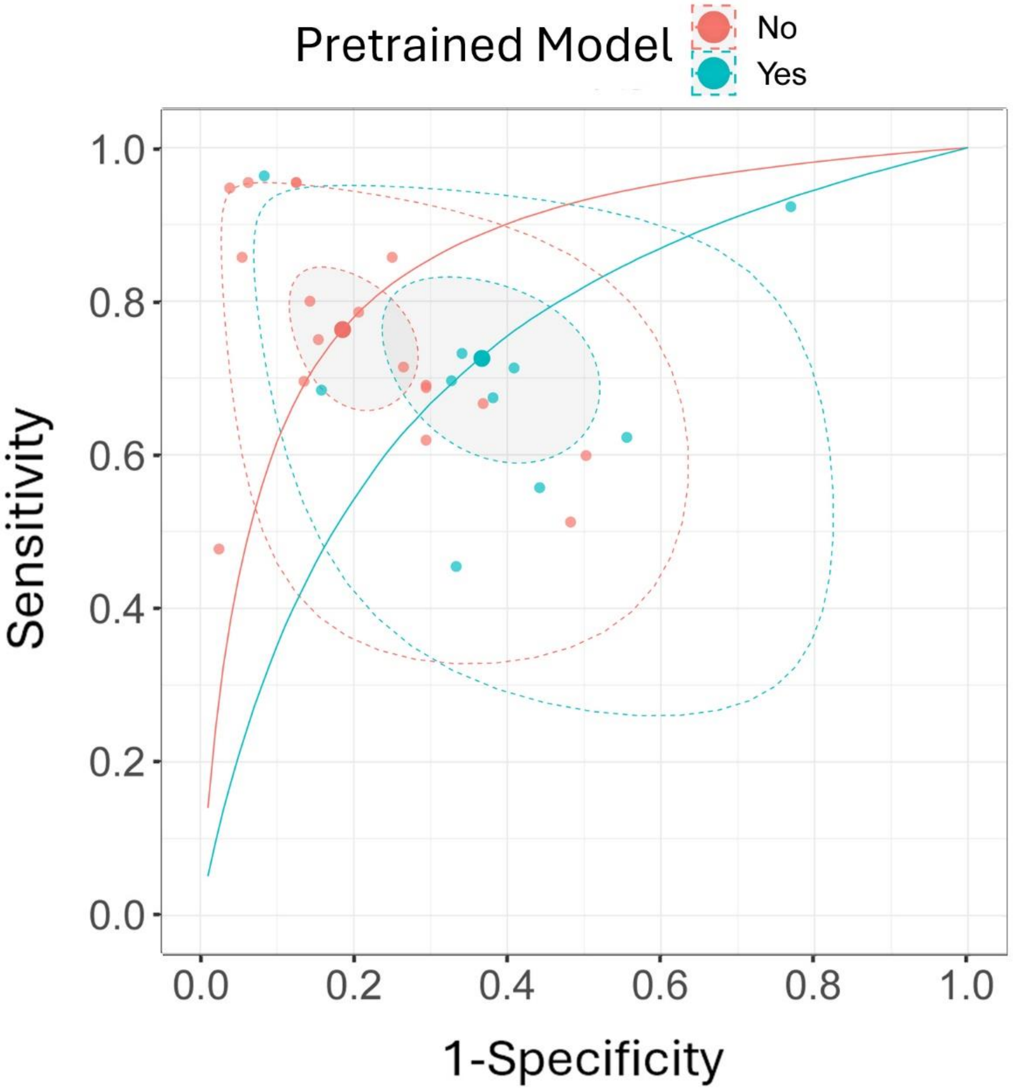


**Figure S33.** MGMT Prediction Comparison in Employing Pretrained Models. The plot shows sensitivity and 1-specificity for using transfer learning (blue curve) versus using models developed from scratch (red curve). Dotted lines indicate the 95% prediction region, while shaded areas represent the 95% credible region from the bivariate model.

### 10.7.2. Accuracy vs. Covariate Plot

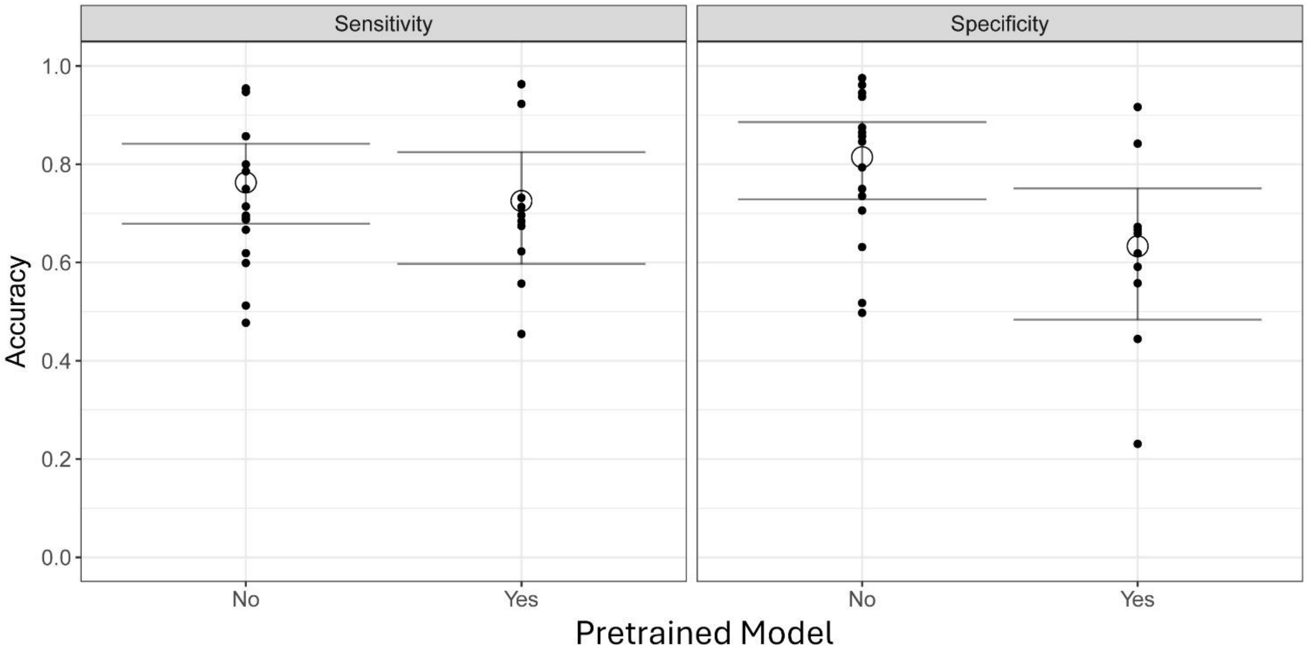


**Figure S34.** Accuracy vs. Pretrained Model Plot for MGMT Prediction. The left panel shows sensitivity, and the right panel shows specificity for using transfer learning versus models developed from scratch. Vertical bars represent the 95% credible intervals from the bivariate model, while black points denote study-specific data points for each method.

## 10.8. Deep Learning Integration Level

### 10.8.1. SROC curves

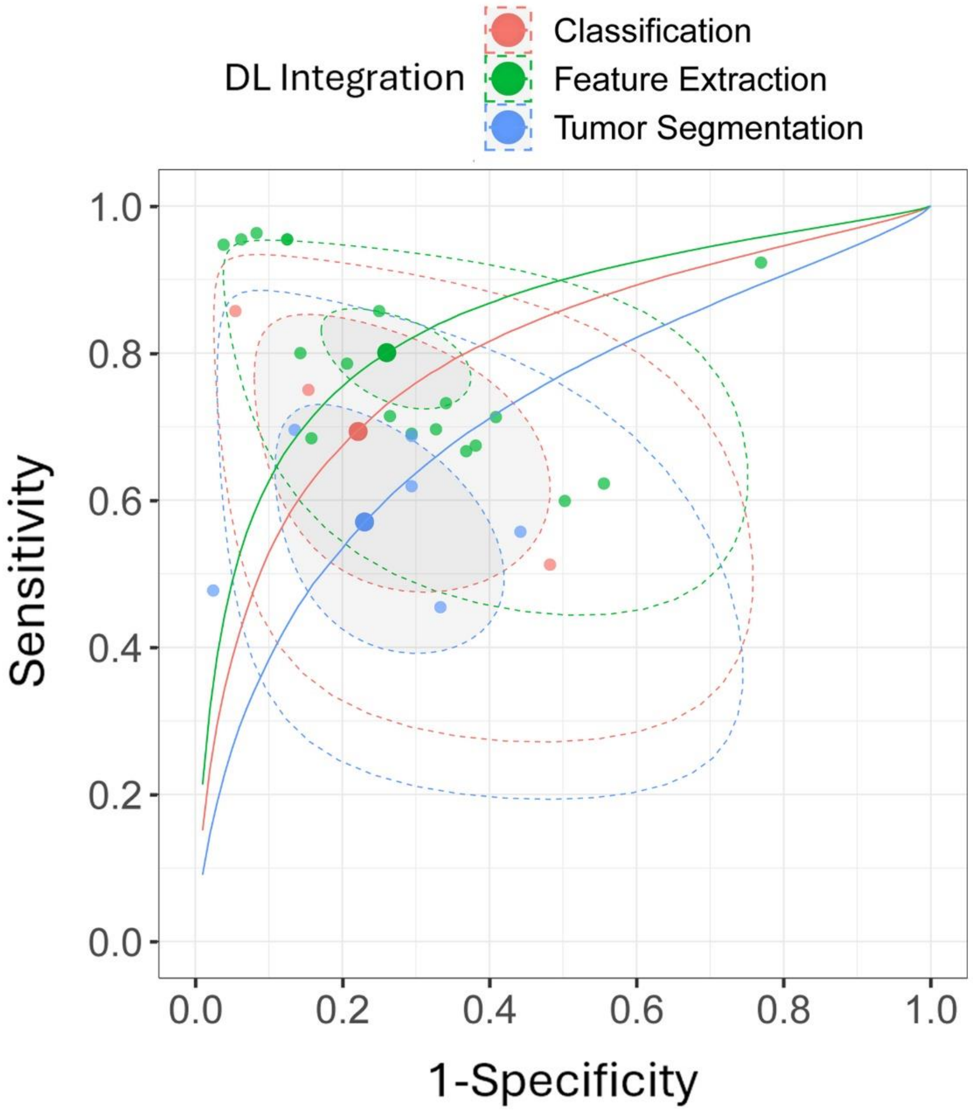


**Figure S35.** MGMT Prediction Comparison in Deep Learning Integration Level. The SROC plot shows sensitivity and 1-specificity for different levels of deep learning integration: classification (red curve), feature extraction (green curve), and tumor segmentation (blue curve). Dotted lines indicate the 95% prediction region, while shaded areas represent the 95% credible region from the bivariate model.

### 10.8.2. Accuracy vs. Covariate Plot

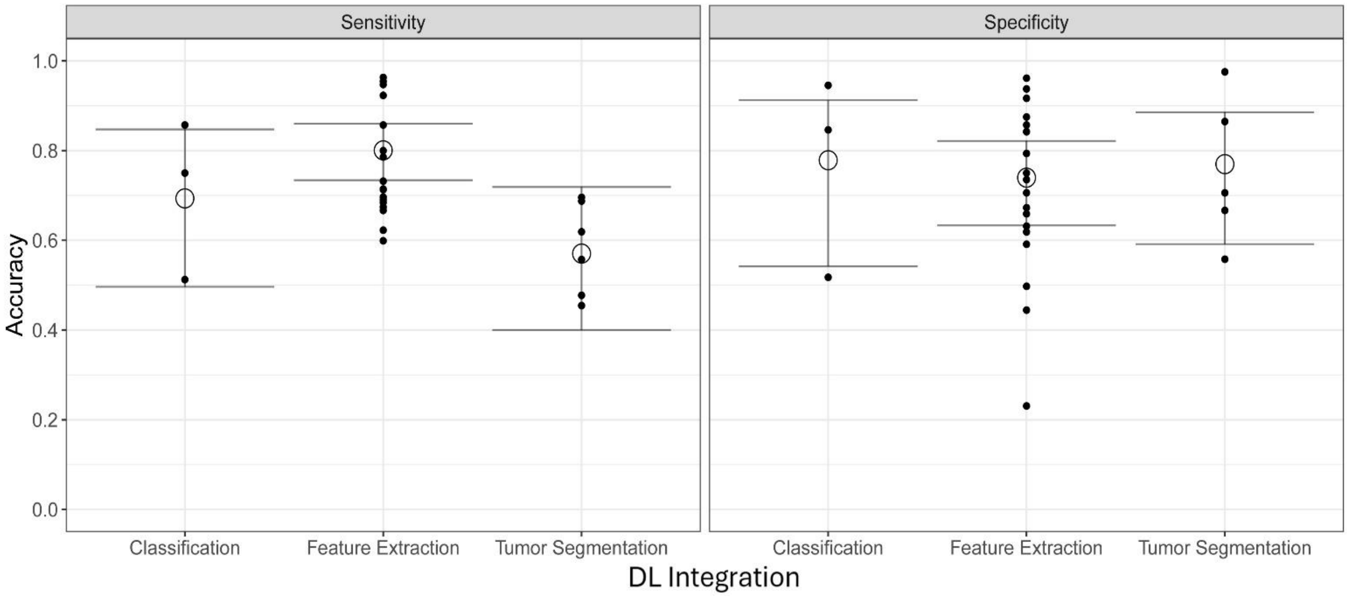


**Figure S36.** Accuracy vs. Deep Learning Integration Level Plot for MGMT Prediction. The left panel shows sensitivity, and the right panel shows specificity for different levels of deep learning methods in the radiomics workflow (tumor segmentation, feature extraction, and classification). Vertical bars represent the 95% credible intervals from the bivariate model, while black points denote study-specific data points for each method.

## 10.9. MRI

### 10.9.1. SROC curves

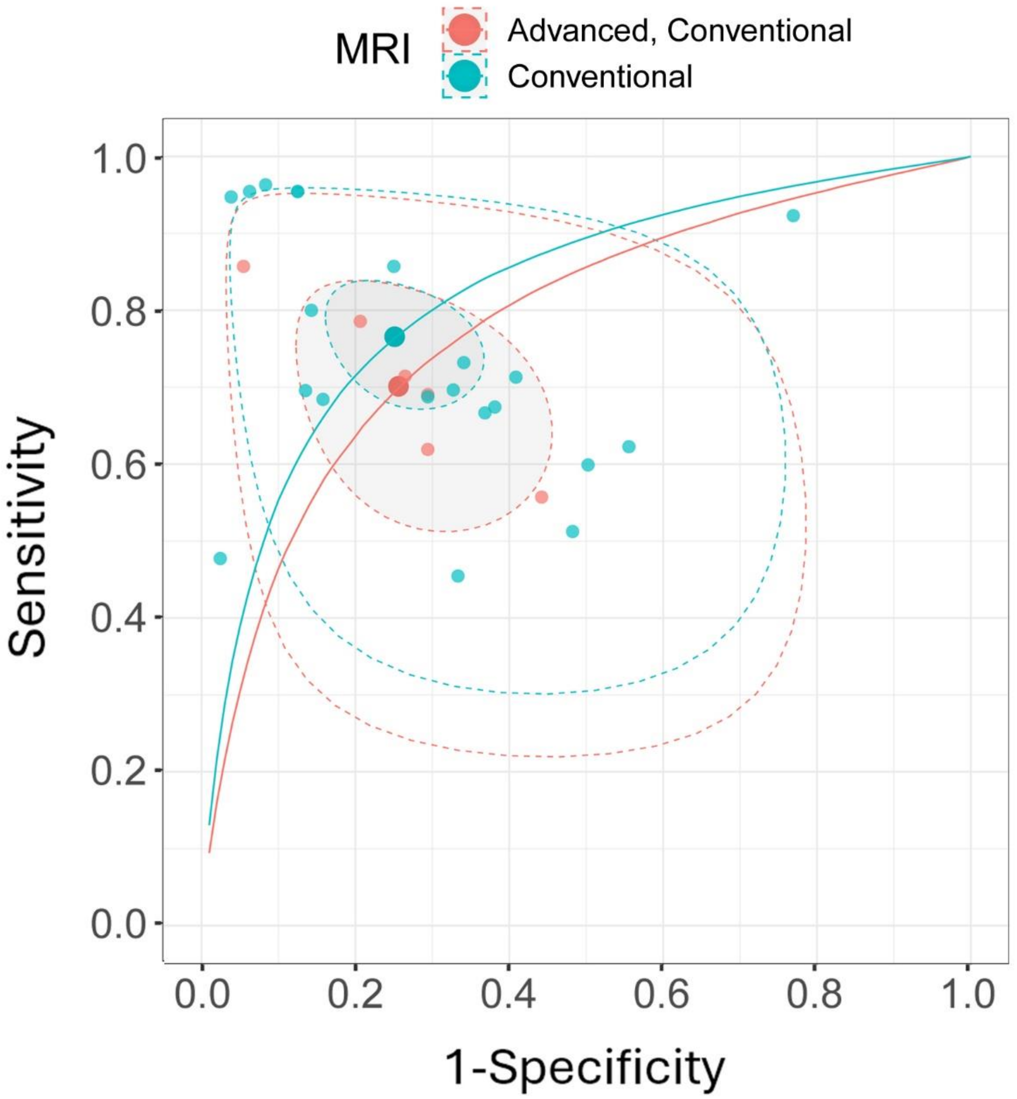


**Figure S37.** MGMT Prediction Comparison in MRI Techniques. The plot shows sensitivity and 1-specificity for conventional MRI sequences (T1, T2, and T2-FLAIR) and a combination of advanced (ADC, DSC, and SWI) and conventional techniques. Dotted lines indicate the 95% prediction region, while shaded areas represent the 95% credible region from the bivariate model.

### 10.9.2. Accuracy vs. Covariate Plot

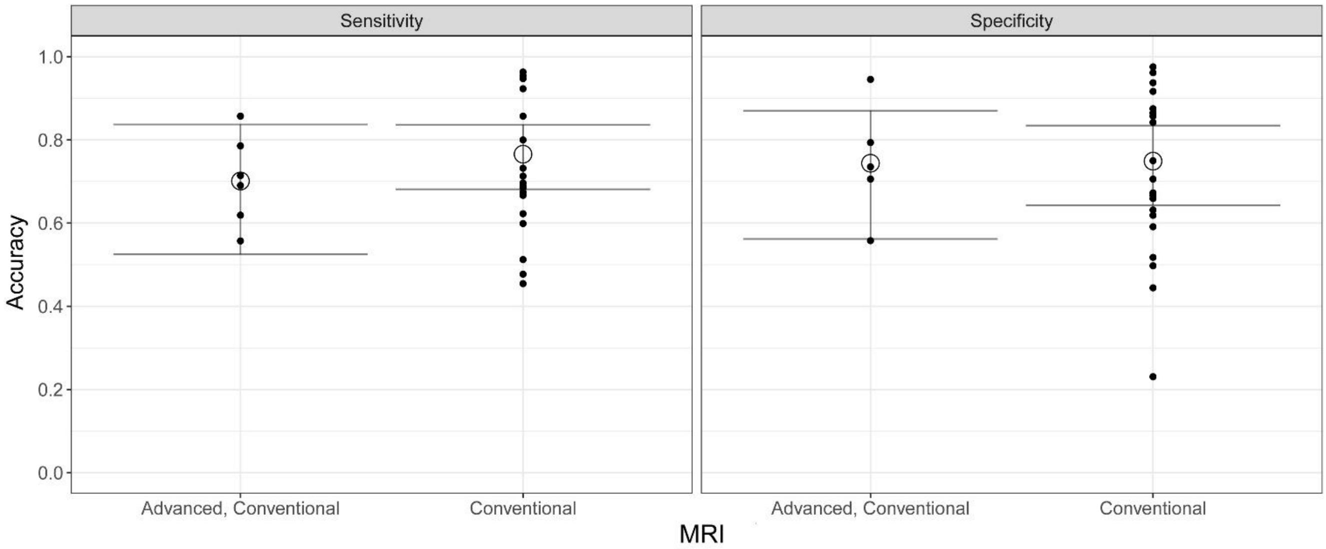


**Figure S38.** Accuracy vs. MRI Technique Plot for MGMT Prediction. The left panel shows sensitivity, and the right panel shows specificity for different MRI techniques. Vertical bars represent the 95% credible intervals from the bivariate model, while black points denote study-specific data points for each method.

## 10.10. MRI Sequences

### 10.10.1. SROC curves

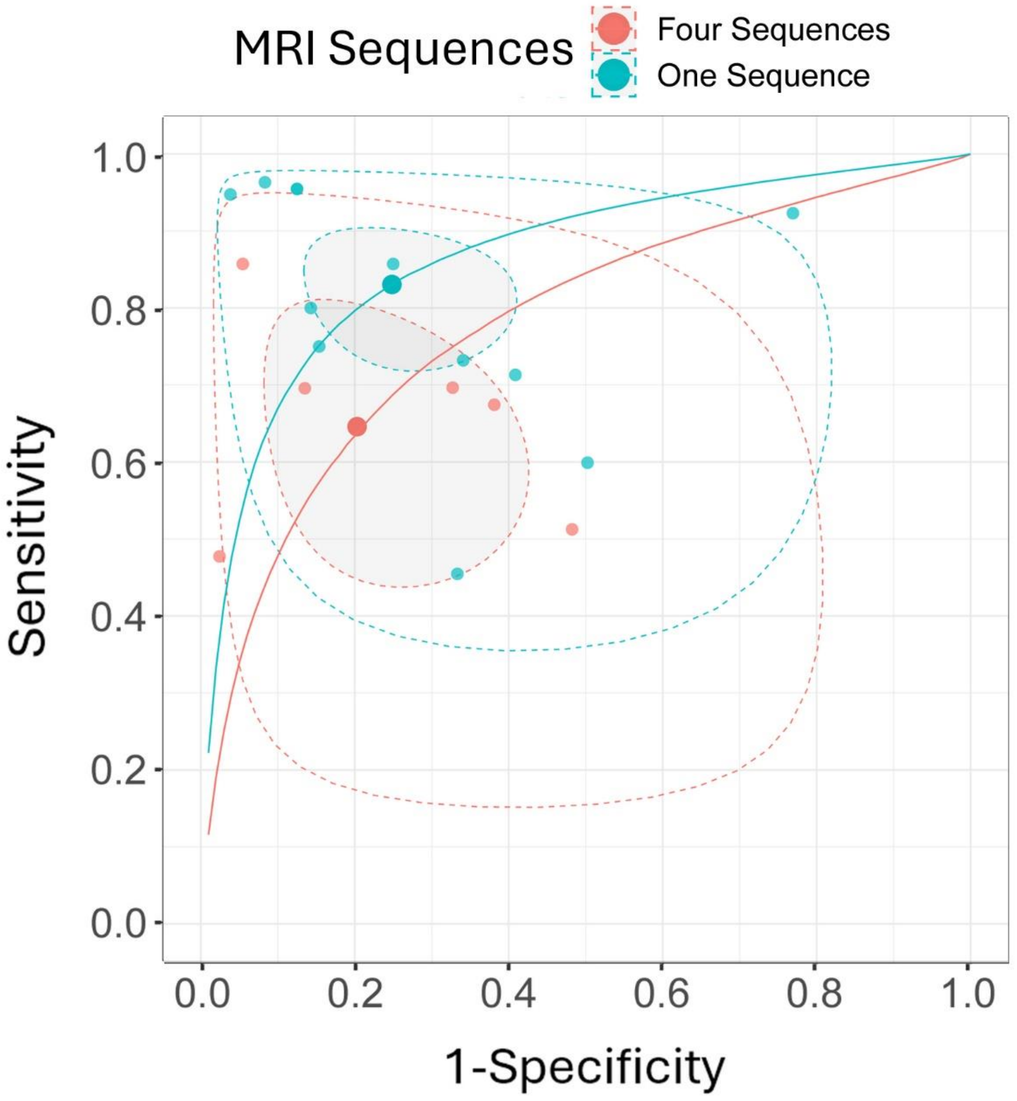


**Figure S39.** MGMT Prediction Comparison in Number of MRI Sequences: The SROC plot shows sensitivity and 1-specificity for using four MRI sequences (red curve) versus one MRI sequence (blue curve). Dotted lines indicate the 95% prediction region, while shaded areas represent the 95% credible region from the bivariate model.

### 10.10.2. Accuracy vs. Covariate Plot

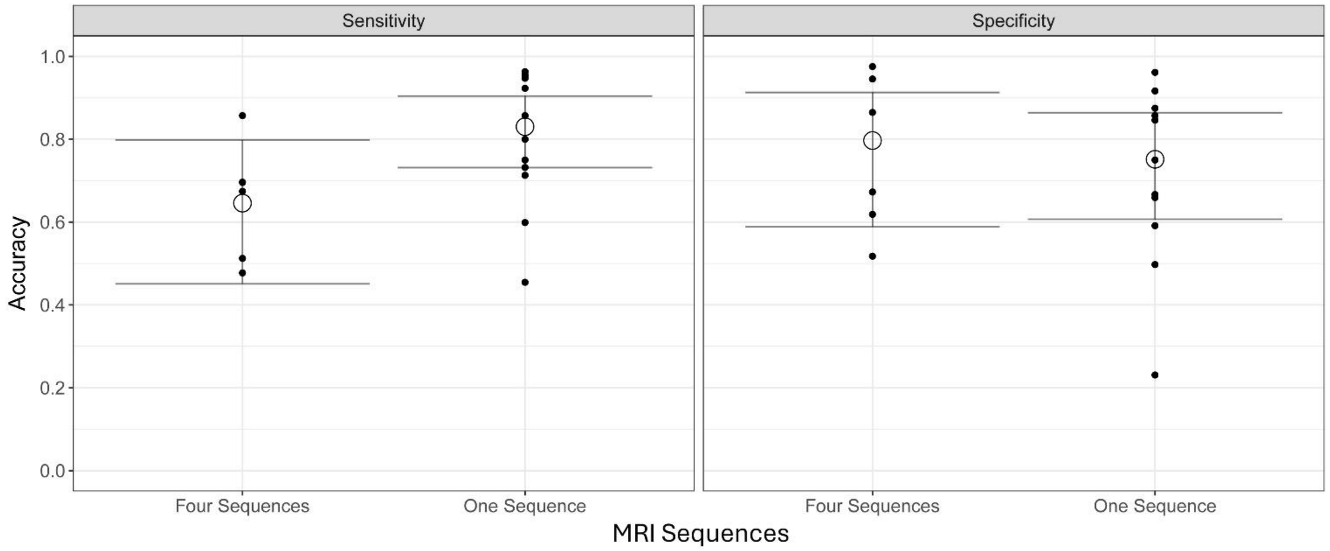


**Figure S40.** Accuracy vs. Number of MRI Sequences Plot for MGMT Prediction. The left panel shows sensitivity, and the right panel shows specificity for different numbers of MRI sequences used (four sequences and one sequence). Vertical bars represent the 95% credible intervals from the bivariate model, while black points denote study-specific data points for each method.

## 10.11. Validation Method

### 10.11.1. SROC curves

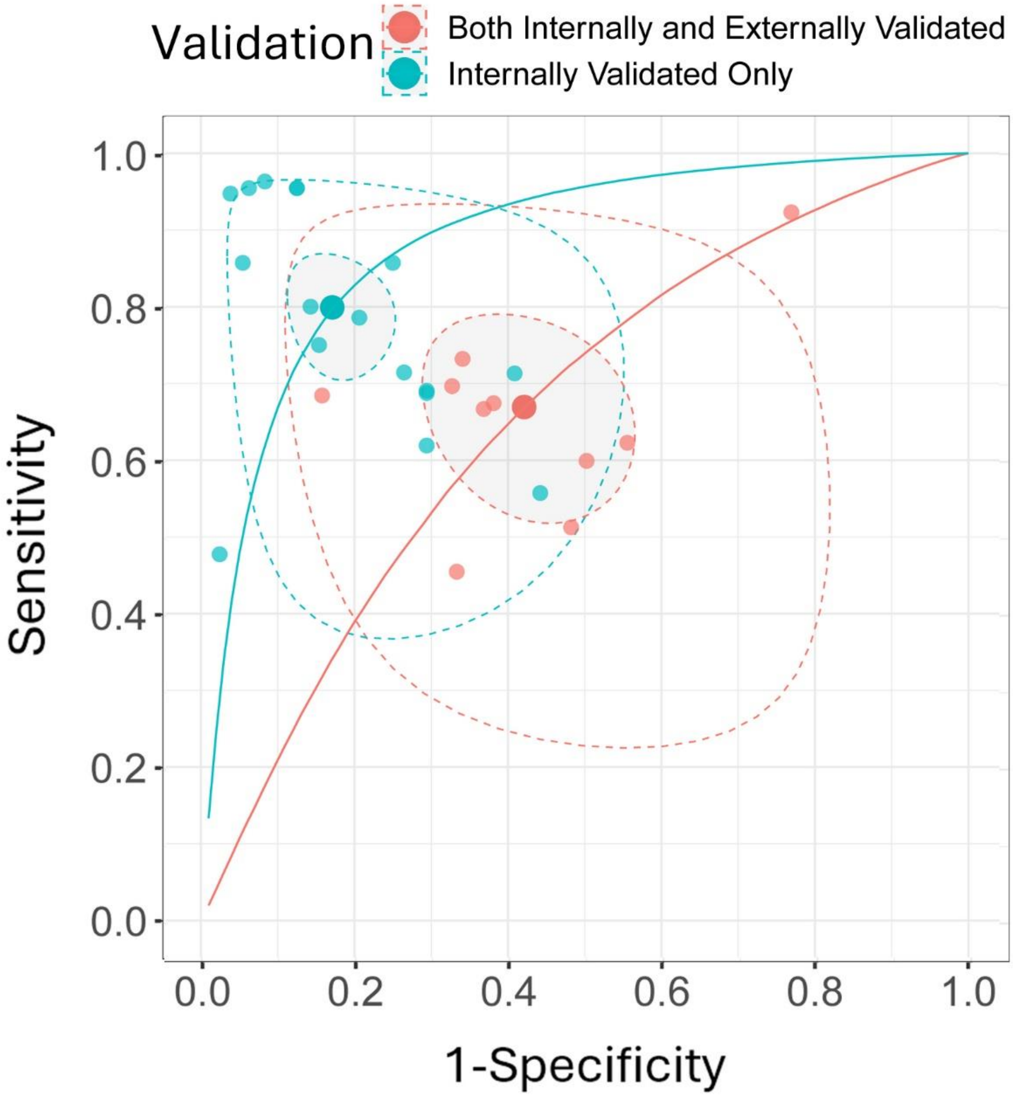


**Figure S41.** MGMT Prediction Comparison in Validation Methods. The SROC plot shows sensitivity and 1-specificity for models with both internal and external validation (red curve) versus internal validation only (blue curve). Dotted lines indicate the 95% prediction region, while shaded areas represent the 95% credible region from the bivariate model.

### 10.11.2. Accuracy vs. Covariate Plot

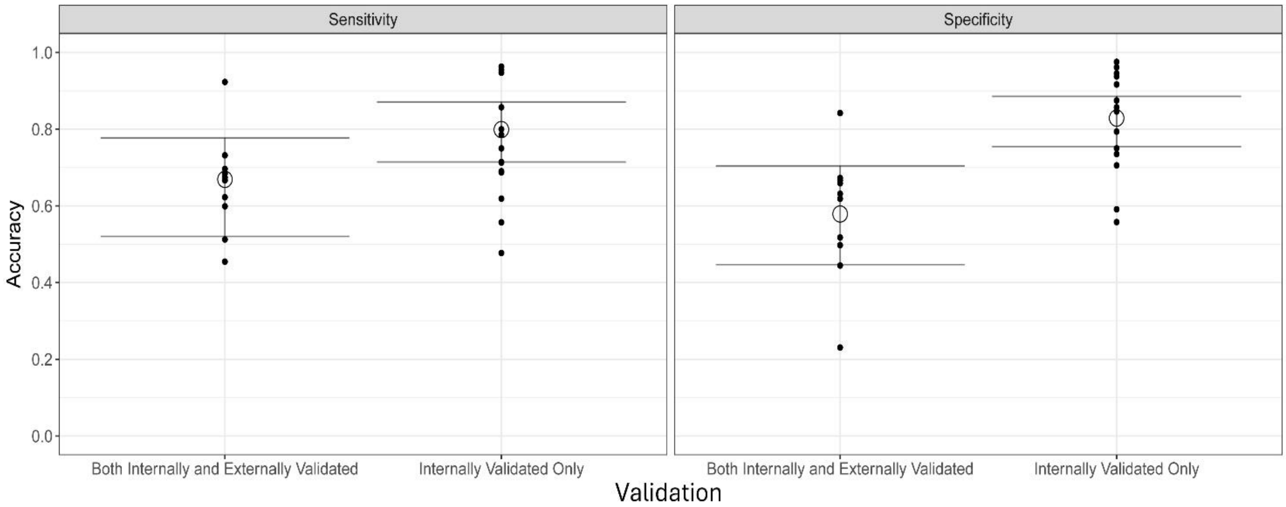


**Figure S42.** Accuracy vs. Validation Method Plot for MGMT Prediction. The left panel shows sensitivity, and the right panel shows specificity for different validation approaches (internally validated only and both internally and externally validated models). Vertical bars represent the 95% credible intervals from the bivariate model, while black points denote study-specific data points for each method.

## 10.12. Internal Validation

### 10.12.1. SROC curves

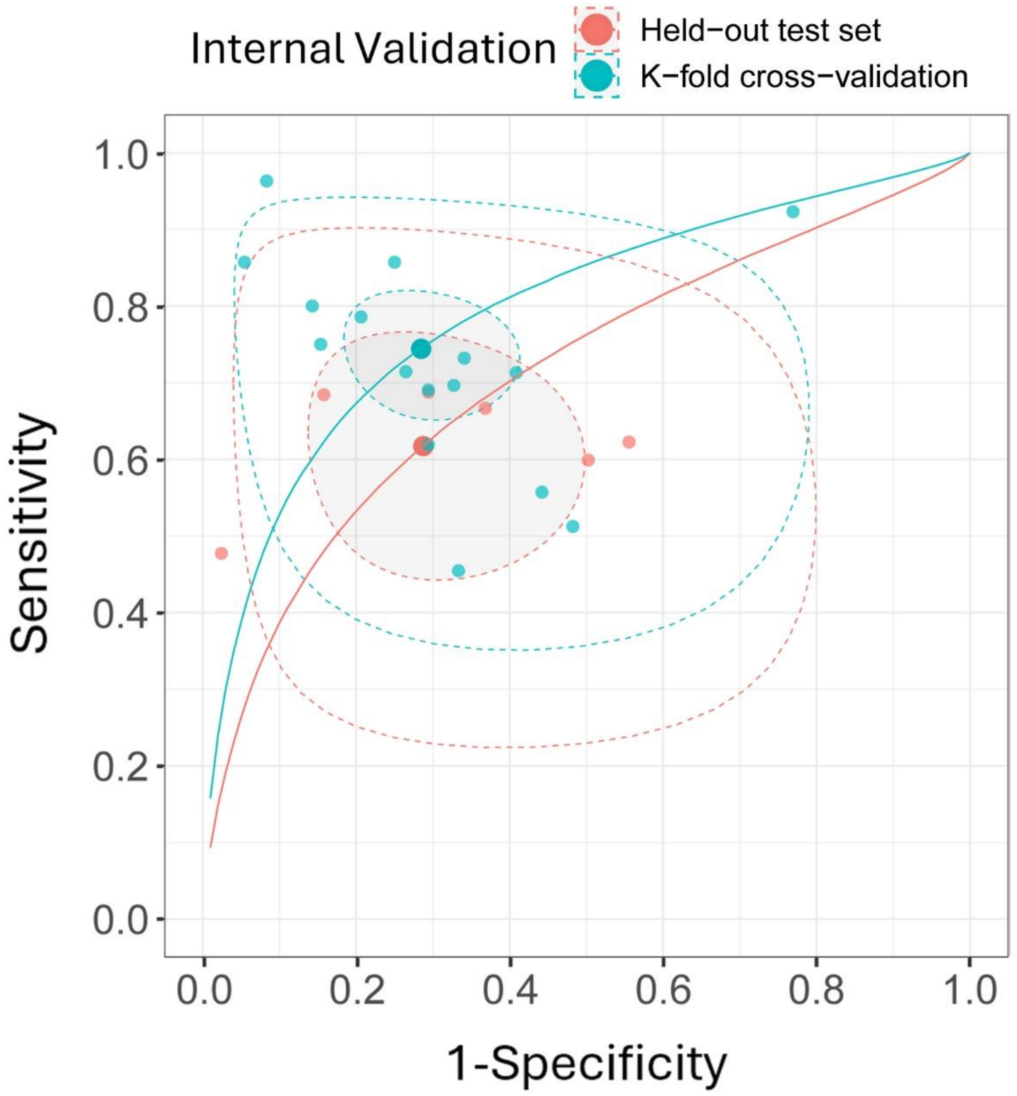


**Figure S43.** MGMT Prediction Comparison in Internal Validation Methods. The SROC plot shows sensitivity and 1-specificity for held-out test set (red curve) and k-fold cross-validation (blue curve). Dotted lines indicate the 95% prediction region, while shaded areas represent the 95% credible region from the bivariate model.

### 10.12.2. Accuracy vs. Covariate Plot

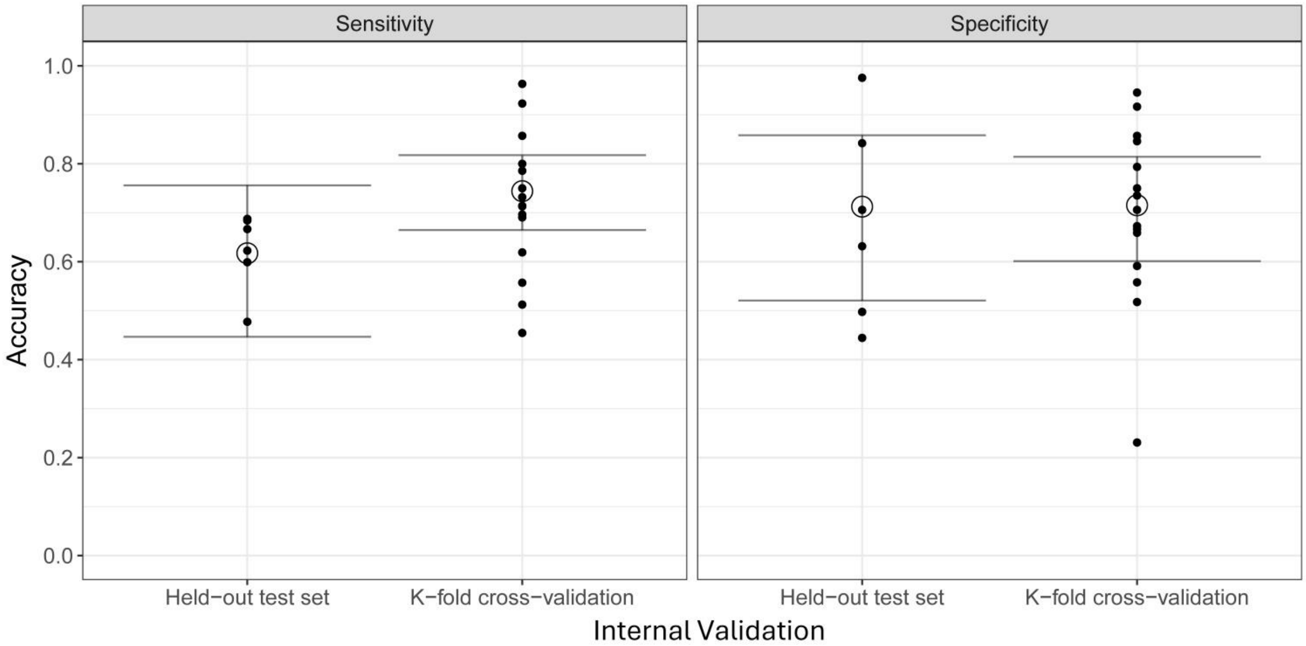


**Figure S44.** Accuracy vs. Internal Validation Method Plot for MGMT Prediction. The left panel shows sensitivity, and the right panel shows specificity for different internal validation methods (held-out test set and k-fold cross-validation). Vertical bars represent the 95% credible intervals from the bivariate model, while black points denote study-specific data points for each method.

## 10.13. RQS

### 10.13.1. SROC curves

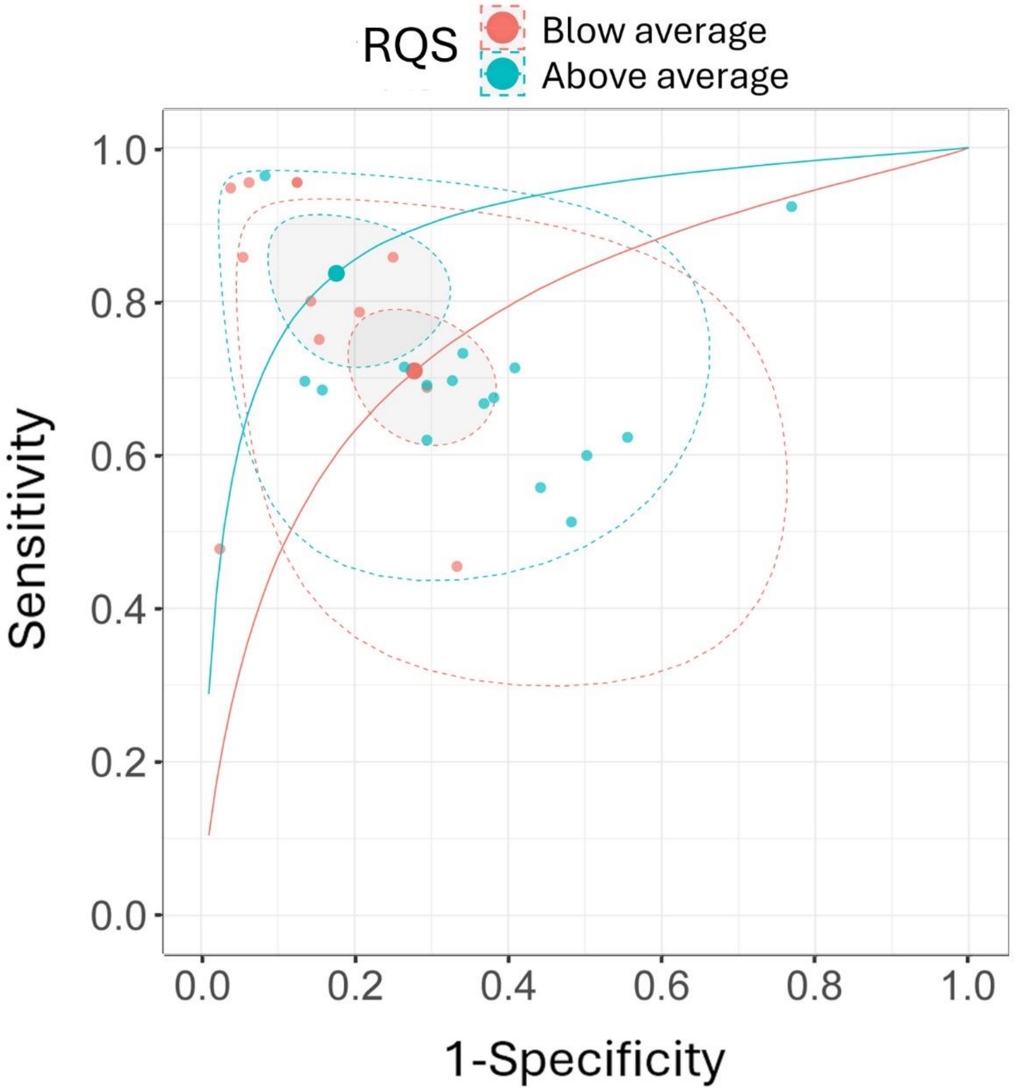


**Figure S45.** MGMT Prediction Comparison in Radiomics Quality Scores (RQSs). The plot shows sensitivity and 1-specificity for below the average score (average = 37%, red curve) and above the average (blue curve). Dotted lines indicate the 95% prediction region, while shaded areas represent the 95% credible region from the bivariate model.

### 10.13.2. Accuracy vs. Covariate Plot

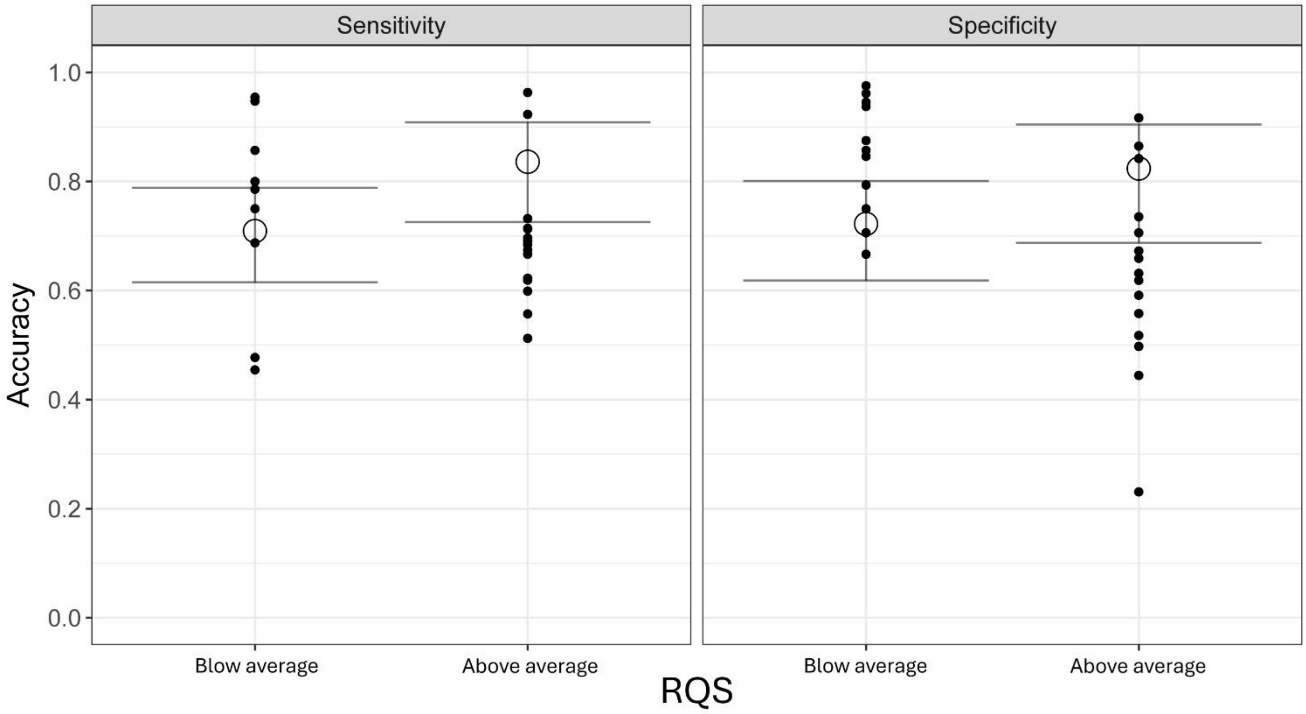


**Figure S46.** Accuracy vs. Radiomics Quality Score (RQS) Plot for MGMT Prediction. The left panel shows sensitivity, and the right panel shows specificity for below and above the average score (average = 37%). Vertical bars represent the 95% credible intervals from the bivariate model, while black points denote study-specific data points for each score level.

## 11. MRI Sequences

The contribution of MRI sequences in the reviewed studies was categorized based on the number of sequences used, ranging from one to four. The results, depicted in Figure 45, show a diverse range of sequence applications. Most studies (31.0%) utilized four sequences. Single-sequence studies primarily employed T2-weighted imaging and T2-Fluid Attenuated Inversion Recovery (T2-FLAIR). For two-sequence studies, T1-weighted contrast-enhanced imaging (T1CE) was commonly paired with T2-FLAIR, while three-sequence studies frequently included T1-weighted imaging (T1), T1CE, and T2-weighted imaging (T2).

Our analysis also reveals that advanced MRI sequences, specifically Dynamic Susceptibility Contrast (DSC), were used exclusively in one study. The combination of advanced and conventional sequences was present in 14.29% of the studies, whereas conventional sequences alone accounted for 83.33%. Advanced sequences, such as DSC, Diffusion-Weighted Imaging (DWI), Apparent Diffusion Coefficient (ADC), and Quantitative Susceptibility Mapping (QSM), were employed in several studies. Among two-sequence studies, combinations like T1CE with ADC and T1CE with DWI were each used in 12.5% of the cases. In three-sequence studies, DSC was combined with T1 and T1CE in 11.1% of the cases. Studies with more than four sequences featured complex combinations, including Susceptibility Weighted Imaging (SWI), Arterial Spin Labeling (ASL), and 2D 55-direction High Angular Resolution Diffusion Imaging (HARDI), along with T1, T1CE, T2, and T2-FLAIR. These advanced sequences are essential for improving MRI's diagnostic capabilities in glioma studies by providing valuable functional and structural information that complements standard imaging sequences.

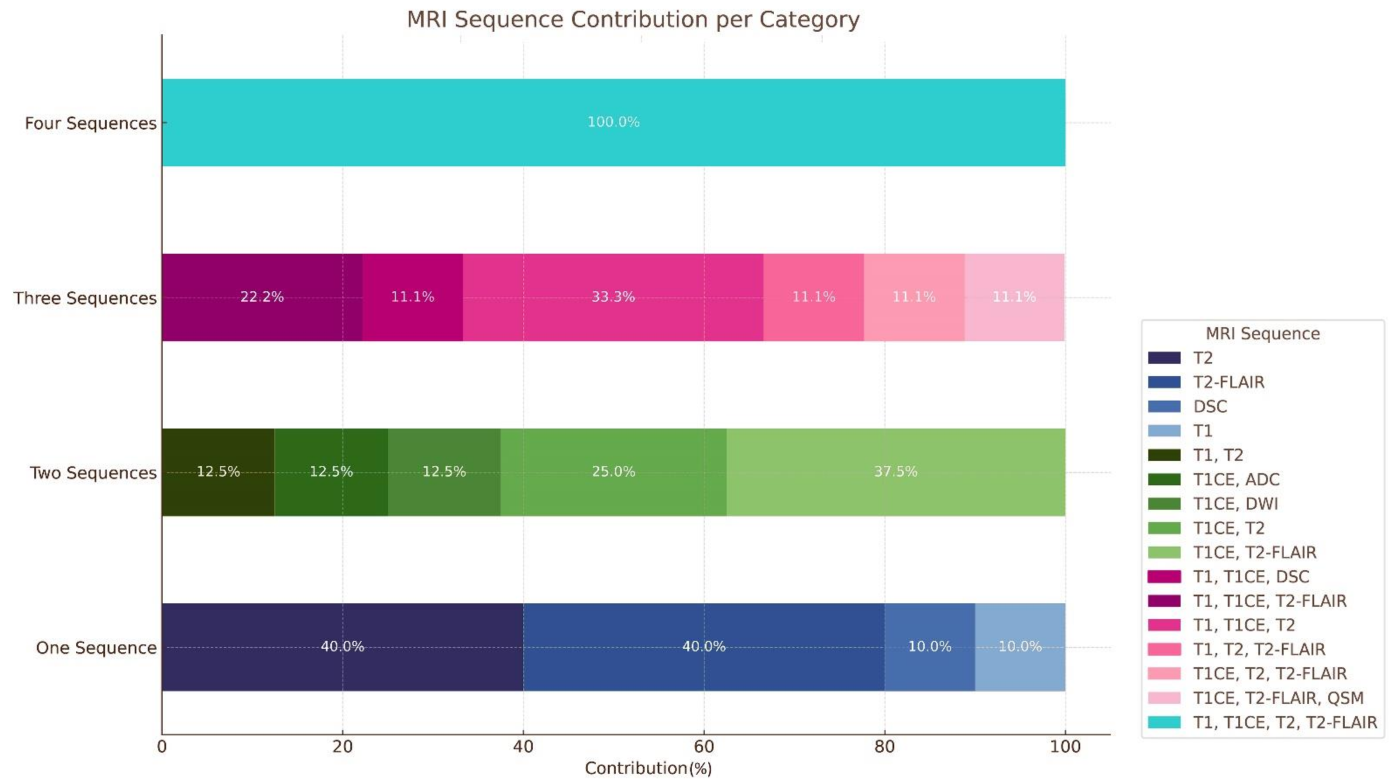


**Figure S47.** Contribution of MRI sequences in studies grouped by the number of sequences used—from single to four sequences—highlighting the specific sequence combinations and their prevalence in each category.

# 13. References


1. Korfiatis P, Kline TL, Lachance DH, et al (2017) Residual Deep Convolutional Neural Network Predicts MGMT Methylation Status. J Digit Imaging 30:622–628. https://doi.org/10.1007/s10278-017-0009-z

2. Li Z-C, Bai H, Sun Q, et al (2018) Multiregional radiomics features from multiparametric MRI for prediction of MGMT methylation status in glioblastoma multiforme: A multicentre study. Eur Radiol 28:3640–3650. https://doi.org/10.1007/s00330-017-5302-1

3. Chang P, Grinband J, Weinberg BD, et al (2018) Deep-Learning Convolutional Neural Networks Accurately Classify Genetic Mutations in Gliomas. Am J Neuroradiol 39:1201–1207. https://doi.org/10.3174/ajnr.A5667

4. Han L, Kamdar MR (2018) MRI to MGMT: predicting methylation status in glioblastoma patients using convolutional recurrent neural networks. In: Altman RB, Dunker AK, Hunter L, et al (eds) PACIFIC SYMPOSIUM ON BIOCOMPUTING 2018 (PSB). World Scientific Publ Co Pte Ltd, Singapore, pp 331–342

5. Korfiatis P, Kline TL, Erickson BJ (2018) Evaluation of a deep learning architecture for MR imaging prediction of ATRX in glioma patients. In: Petrick N, Mori K (eds) MEDICAL IMAGING 2018: COMPUTER-AIDED DIAGNOSIS. Spie-Int Soc Optical Engineering, Bellingham, p UNSP 105752G

6. Fukuma R, Yanagisawa T, Kinoshita M, et al (2019) Prediction of IDH and TERT promoter mutations in low-grade glioma from magnetic resonance images using a convolutional neural network. Sci Rep 9:20311. https://doi.org/10.1038/s41598-019-56767-3

7. Tang Z, Xu Y, Jin L, et al (2020) Deep Learning of Imaging Phenotype and Genotype for Predicting Overall Survival Time of Glioblastoma Patients. IEEE Trans Med IMAGING 39:2100–2109. https://doi.org/10.1109/TMI.2020.2964310

8. Crisi G, Filice S (2020) Predicting MGMT Promoter Methylation of Glioblastoma from Dynamic Susceptibility Contrast Perfusion: A Radiomic Approach. J NEUROIMAGING 30:458–462. https://doi.org/10.1111/jon.12724

9. Hedyehzadeh M, Maghooli K, MomenGharibvand M, Pistorius S (2020) A Comparison of the Efficiency of Using a Deep CNN Approach with Other Common Regression Methods for the Prediction of EGFR Expression in Glioblastoma Patients. J Digit Imaging 33:391–398. https://doi.org/10.1007/s10278-019-00290-4

10. Calabrese E, Villanueva-Meyer JE, Cha S (2020) A fully automated artificial intelligence method for non-invasive, imaging-based identification of genetic alterations in glioblastomas. Sci Rep 10:11852. https://doi.org/10.1038/s41598-020-68857-8

11. Chen X, Zeng M, Tong Y, et al (2020) Automatic Prediction of MGMT Status in Glioblastoma via Deep Learning-Based MR Image Analysis. BioMed Res Int 2020:9258649. https://doi.org/10.1155/2020/9258649

12. Jonnalagedda P, Weinberg B, Allen J, Bhanu B (2020) Feature Disentanglement to Aid Imaging Biomarker Characterization for Genetic Mutations. pp 349–364

13. Haubold J, Hosch R, Parmar V, et al (2021) Fully Automated MR Based Virtual Biopsy of Cerebral Gliomas. Cancers 13:6186. https://doi.org/10.3390/cancers13246186

14. Yogananda CGB, Shah BR, Nalawade SS, et al (2021) MRI-Based Deep-Learning Method for Determining Glioma *MGMT* Promoter Methylation Status. Am J Neuroradiol 42:845–852. https://doi.org/10.3174/ajnr.A7029

15. Tupe-Waghmare P, Malpure P, Kotecha K, et al (2021) Comprehensive Genomic Subtyping of Glioma Using Semi-Supervised Multi-Task Deep Learning on Multimodal MRI. IEEE ACCESS 9:167900–167910. https://doi.org/10.1109/ACCESS.2021.3136293

16. Chen H., Lin F., Zhang J., et al (2021) Deep Learning Radiomics to Predict PTEN Mutation Status From Magnetic Resonance Imaging in Patients With Glioma. Front Oncol 11:734433. https://doi.org/10.3389/fonc.2021.734433

17. Lang DM, Peeken JC, Combs SE, et al (2022) A Video Data Based Transfer Learning Approach for Classification of MGMT Status in Brain Tumor MR Images. In: Crimi A, Bakas S (eds) BRAINLESION: GLIOMA, MULTIPLE SCLEROSIS, STROKE AND TRAUMATIC BRAIN INJURIES, BRAINLES 2021, PT I. Springer International Publishing Ag, Cham, pp 306–314

18. Xiao Z, Yao S, Wang Z, et al (2021) Multiparametric MRI Features Predict the SYP Gene Expression in Low-Grade Glioma Patients: A Machine Learning-Based Radiomics Analysis. Front Oncol 11:. https://doi.org/10.3389/fonc.2021.663451

19. Sohn B, An C, Kim D, et al (2021) Radiomics-based prediction of multiple gene alteration incorporating mutual genetic information in glioblastoma and grade 4 astrocytoma, IDH-mutant. J Neurooncol 155:267–276. https://doi.org/10.1007/s11060-021-03870-z

20. Capuozzo S, Gravina M, Gatta G, et al (2022) A Multimodal Knowledge-Based Deep Learning Approach for MGMT Promoter Methylation Identification. J IMAGING 8:321. https://doi.org/10.3390/jimaging8120321

21. Chen S., Xu Y., Ye M., et al (2022) Predicting MGMT Promoter Methylation in Diffuse Gliomas Using Deep Learning with Radiomics. J Clin Med 11:3445. https://doi.org/10.3390/jcm11123445

22. Calabrese E, Rudie JD, Rauschecker AM, et al (2022) Combining radiomics and deep convolutional neural network features from preoperative MRI for predicting clinically relevant genetic biomarkers in glioblastoma. Neuro-Oncol Adv 4:. https://doi.org/10.1093/noajnl/vdac060

23. Farzana W, Temtam AG, Shboul ZA, et al (2022) Radiogenomic Prediction of MGMT Using Deep Learning with Bayesian Optimized Hyperparameters. In: Crimi A, Bakas S (eds) BRAINLESION: GLIOMA, MULTIPLE SCLEROSIS, STROKE AND TRAUMATIC BRAIN INJURIES, BRAINLES 2021, PT II. Springer International Publishing Ag, Cham, pp 357–366

24. Kim B-H, Lee H, Choi KS, et al (2022) Validation of MRI-Based Models to Predict MGMT Promoter Methylation in Gliomas: BraTS 2021 Radiogenomics Challenge. CANCERS 14:4827. https://doi.org/10.3390/cancers14194827

25. Nalawade SS, Yu FF, Bangalore Yogananda CG, et al (2022) Brain tumor IDH, 1p/19q, and MGMT molecular classification using MRI-based deep learning: an initial study on the effect of motion and motion correction. J Med IMAGING 9:016001. https://doi.org/10.1117/1.JMI.9.1.016001

26. Xu Q, Xu QQ, Shi N, et al (2022) A multitask classification framework based on vision transformer for predicting molecular expressions of glioma. Eur J Radiol 157:110560. https://doi.org/10.1016/j.ejrad.2022.110560

27. K R S, Mahdev AR, B V, J SML (2022) Deep Learning Approach for Radiogenomic Classification of Brain Tumor. In: 2022 IEEE 19th India Council International Conference (INDICON). pp 1–6

28. Kihira S, Mei X, Mahmoudi K, et al (2022) U-Net Based Segmentation and Characterization of Gliomas. CANCERS 14:4457. https://doi.org/10.3390/cancers14184457

29. Chaddad A, Hassan L, Katib Y (2023) A texture-based method for predicting molecular markers and survival outcome in lower grade glioma. Appl Intell 53:24724–24738. https://doi.org/10.1007/s10489-023-04844-6

30. Faghani S, Khosravi B, Moassefi M, et al (2023) A Comparison of Three Different Deep Learning-Based Models to Predict the MGMT Promoter Methylation Status in Glioblastoma Using Brain MRI. J Digit Imaging 36:837–846. https://doi.org/10.1007/s10278-022-00757-x

31. Chu W, Zhou Y, Cai S, et al (2024) A Comprehensive Multi-modal Domain Adaptative Aid Framework for Brain Tumor Diagnosis

32. Rui W, Zhang S, Shi H, et al (2023) Deep Learning-Assisted Quantitative Susceptibility Mapping as a Tool for Grading and Molecular Subtyping of Gliomas. PHENOMICS 3:243–254. https://doi.org/10.1007/s43657-022-00087-6

33. Saeed N, Ridzuan M, Alasmawi H, et al (2023) MGMT promoter methylation status prediction using MRI scans? An extensive experimental evaluation of deep learning models. Med Image Anal 90:102989. https://doi.org/10.1016/j.media.2023.102989

34. Sakly H, Said M, Seekins J, et al (2023) Brain Tumor Radiogenomic Classification of O6-Methylguanine-DNA Methyltransferase Promoter Methylation in Malignant Gliomas-Based Transfer Learning. Cancer Control J Moffitt Cancer Cent 30:10732748231169149. https://doi.org/10.1177/10732748231169149

35. Zhang H, Zhang H, Zhang Y, et al (2023) Deep Learning Radiomics for the Assessment of Telomerase Reverse Transcriptase Promoter Mutation Status in Patients With Glioblastoma Using Multiparametric MRI. J Magn Reson IMAGING 58:1441–1451. https://doi.org/10.1002/jmri.28671

36. Saxena S, Jena B, Mohapatra B, et al (2023) Fused deep learning paradigm for the prediction of o6-methylguanine-DNA methyltransferase genotype in glioblastoma patients: A neuro-oncological investigation. Comput Biol Med 153:106492. https://doi.org/10.1016/j.compbiomed.2022.106492

37. Saxena S, Agrawal A, Dash P, et al (2023) Prediction of O-6-methylguanine-DNA methyltransferase and overall survival of the patients suffering from glioblastoma using MRI-based hybrid radiomics signatures in machine and deep learning framework. NEURAL Comput Appl 35:13647–13663. https://doi.org/10.1007/s00521-023-08405-3

38. Robinet L, Siegfried A, Roques M, et al (2023) MRI-Based Deep Learning Tools for MGMT Promoter Methylation Detection: A Thorough Evaluation. Cancers 15:. https://doi.org/10.3390/cancers15082253

39. Buz-Yalug B, Turhan G, Cetin AI, et al (2024) Identification of IDH and TERTp mutations using dynamic susceptibility contrast MRI with deep learning in 162 gliomas. Eur J Radiol 170:111257. https://doi.org/10.1016/j.ejrad.2023.111257

40. Liu Z, Xu X, Zhang W, et al (2024) A fusion model integrating magnetic resonance imaging radiomics and deep learning features for predicting alpha-thalassemia X-linked intellectual disability mutation status in isocitrate dehydrogenase–mutant high-grade astrocytoma: a multicenter study. Quant Imaging Med Surg 14:251–263. https://doi.org/10.21037/qims-23-807

41. Zhang L, Wang R, Gao J, et al (2024) A novel MRI-based deep learning networks combined with attention mechanism for predicting CDKN2A/B homozygous deletion status in IDH-mutant astrocytoma. Eur Radiol 34:391–399. https://doi.org/10.1007/s00330-023-09944-y

42. Zhang H, Zhou B, Zhang H, et al (2024) Peritumoural Radiomics for Identification of Telomerase Reverse Transcriptase Promoter Mutation in Patients With Glioblastoma Based on Preoperative MRI. Can Assoc Radiol J 75:143–152. https://doi.org/10.1177/08465371231183309

43. Chen Xiaohua, Zhang Ruodi, Zhou Yunshu, et al (2023) Multi-sequence MRI-based convolutional neural network predicts the methylation status of MGMT promoter in glioma. Chin J Magn Reson Imaging 14:34-39,78
